# EGTA reduces the inflorescence stem mechanical strength of herbaceous peony by modifying secondary wall biosynthesis

**DOI:** 10.1038/s41438-019-0117-7

**Published:** 2019-03-01

**Authors:** Yuhan Tang, Daqiu Zhao, Jiasong Meng, Jun Tao

**Affiliations:** Jiangsu Key Laboratory of Crop Genetics and Physiology, College of Horticulture and Plant Protection, Yangzhou University, Yangzhou, 225009 Jiangsu China

**Keywords:** Plant physiology, Proteomics, Cell wall

## Abstract

The mechanical strength of inflorescence stems is an important trait in cut flowers. Calcium ions (Ca^2+^) play a pivotal role in maintaining stem strength, but little is known about the underlying molecular mechanisms. In this study, we treated herbaceous peony (*Paeonia lactiflora* Pall.) with ethyl glycol tetraacetic acid (EGTA), an effective Ca^2+^ chelator, and used morphology indicators, spectroscopic analysis, histochemical staining, electron microscopy, and proteomic techniques to investigate the role of Ca^2+^ in inflorescence stem mechanical strength. The EGTA treatment reduced the mechanical strength of inflorescence stems, triggered the loss of Ca^2+^ from cell walls, and reduced lignin in thickened secondary walls in xylem cells as determined by spectroscopic analysis and histochemical staining. Electron microscopy showed that the EGTA treatment also resulted in significantly fewer xylem cell layers with thickened secondary walls as well as in reducing the thickness of these secondary walls. The proteomic analysis showed 1065 differentially expressed proteins (DEPs) at the full-flowering stage (S4). By overlapping the Kyoto encyclopedia of genes and genomes (KEGG) and gene ontology (GO) analysis results, we identified 43 DEPs involved in signal transduction, transport, energy metabolism, carbohydrate metabolism, and secondary metabolite biosynthesis. Using quantitative real-time polymerase chain reaction (qRT-PCR) analysis, we showed that EGTA treatment inhibited Ca^2+^ sensors and secondary wall biosynthesis-related genes. Our findings revealed that EGTA treatment reduced the inflorescence stem mechanical strength by reducing lignin deposition in xylem cells through altering the expression of genes involved in Ca^2+^ binding and secondary wall biosynthesis.

## Cell biology: calcium makes peonies stand tall

Calcium may hold the key to growing peonies with stronger, straighter stems, by enabling the construction of reinforced cell walls, which impart structural support. Peonies are a popular kind of cut flowers, and those with straight stems are more sought after. Although recent studies have hinted at the importance of calcium ions, the mechanism was unclear. To investigate, Jun Tao at Yangzhou University in Jiangsu, China, and colleagues treated herbaceous peonies with ethyl glycol tetraacetic acid (EGTA), a chemical that binds to and removes calcium ions. This resulted in the reduced deposition of a structural protein called lignin in the walls of xylem cells and more pliable stems. Further analysis revealed altered expression of 43 proteins, including those involved in calcium-ion sensing and construction of ‘secondary’ walls in xylem cells within the stem.

## Introduction

Calcium ions (Ca^2+^) play a key role in regulating plant growth and development, including cell wall formation^1^, osmotic regulation^2^, cell division^3^, and resistance to biotic and abiotic stresses^4–6^. In plants, Ca^2+^ signals transient increases in cytosolic free Ca^2+^. Stimulus-induced increases in the concentration of free Ca^2+^ in the cytosol often occur as repetitive oscillations or spiking of cytosolic-free Ca^2+^. Ca^2+^ encodes stimulus-specific information within a so-called Ca^2+^ signature, and thus defines the nature and magnitude of the response^7^. Recently, Ca^2+^ has received increasing attention due to its positive correlation with the straightness of herbaceous peony (*P. lactiflora* Pall.) inflorescence stems, which is a very important quality-assessment parameter for cut flowers^8^. Inflorescence stem straightness is mainly determined by its mechanical strength^9^. After analyzing the Ca^2+^ concentration of inflorescence stems in 76 different *P. lactiflora* cultivars, Li et al.^10^ found a significant positive correlation between Ca^2+^ content and mechanical strength in inflorescence stems. Spraying stems with exogenous 4% calcium chloride (CaCl_2_) also enhanced inflorescence stem mechanical strength^8,11^. However, although the exogenous Ca^2+^ treatment enhances inflorescence stem mechanical strength, no studies have reported the effects of Ca^2+^ deprivation on *P. lactiflora* inflorescence stem mechanical strength.

The mechanical strength in *P. lactiflora* inflorescence stem is related to wall thickness^12^. In plants, wall thickening happens after cell growth is arrested as protoplasts continue to secrete cellulose and other substances into the inner wall. Thickened cell walls are called secondary walls, and they are distributed around the vascular tissues and under the epidermal layer in stems, providing a major mechanical strength to plants^13,14^. Deposition of lignin in xylem elements and sclerenchyma cell walls is important for mechanical strength^9^. Perik et al.^9^ reported that in cut gerbera (*Gerbera jamesonii* cv. Tamara) flowers, inflorescence stem bending was associated with the absence of lignin deposition in sclerenchyma cells. This has also been verified in rice (*Oryza sativa* L.) *brittle culm 3* mutants^15^. In addition to lignin, cellulose synthesis is also essential for proper secondary wall construction^16^.

Ca^2+^ plays a positive role in pollen tube cell wall formation in apple (*Malus pumila* Mill.)^17^ and in the fruit cell walls of jujube (*Zizyphus jujuba* Mill. cv. Dongzao)^18^. However, studies on the effects of Ca^2+^ on cell wall formation and mechanical strength have focused on the physiological level, and the underlying molecular mechanisms remain unclear. Here, we investigated the hypothesis that a lack of Ca^2+^ causes inflorescence stem bending due to reduced mechanical strength. Ethyl glycol tetraacetic acid (EGTA), a Ca^2+^ chelator that binds Ca^2+^^19,20^, was used for Ca^2+^ deprivation treatment. The relationship with mechanical strength was explored by studying the morphological indices of inflorescence stems and flowers, the extension of inflorescence stem secondary walls, inflorescence stem cell wall composition, protein changes in the inflorescence stem, and expression changes of secondary wall biosynthesis-related genes in the inflorescence stem.

## Results

### Morphological indices and photosynthetic characteristics

**Fig. 1:**
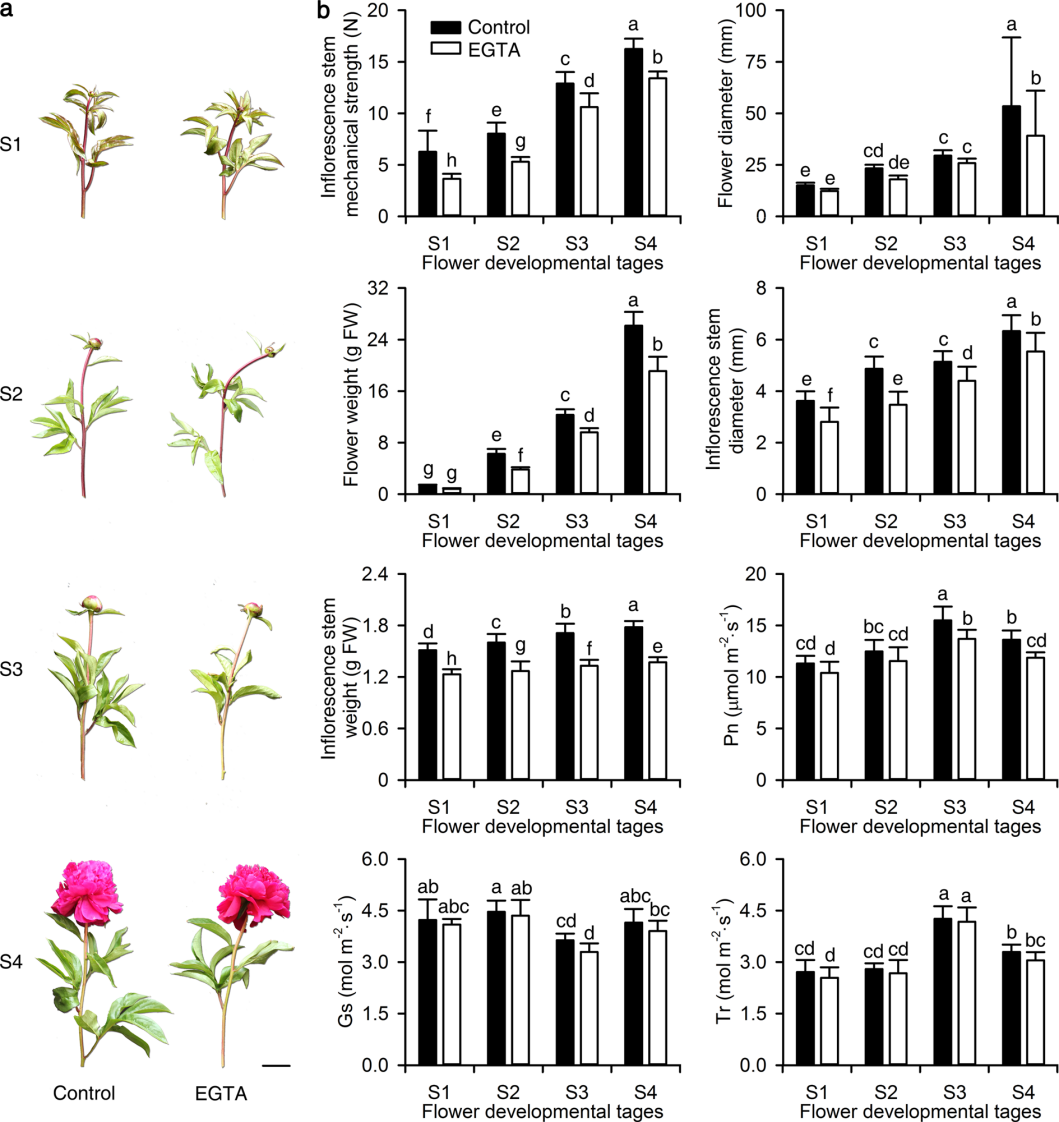
Effects of EGTA treatment on morphological indices and photosynthetic characteristics of *P. lactiflora* at four developmental stages. **a** Photographs of inflorescence stems. Bar = 5 cm. **b** Morphological indices and photosynthetic characteristics. The values represent the means ± SD, and different letters indicate significant differences (*P* < 0.05). Pn, photosynthesis rate; Gs, stomatal conductance; Tr, transpiration rate; S1, flower-bud stage; S2, pigmented stage; S3, unfold-petal stage; S4, full-flowering stage

EGTA treatment significantly affected *P. lactiflora* growth and development. Specifically, the upper part of the *P. lactiflora* inflorescence stems was less straight after EGTA treatment compared to the control at all four flower-development stages (flower-bud stage (S1), pigmented stage (S2), unfold-petal stage (S3), and full-flowering stage (S4); Fig. 1a). To further investigate *P. lactiflora* morphological changes induced by EGTA treatment, five morphological indices, including the upper inflorescence stem mechanical strength, diameter and weight, and the flower diameter and weight at these four flower developmental stages were measured. Values for all five morphological indices were significantly lower in plants treated with EGTA compared to controls. The upper inflorescence stem mechanical strength, which was an average of 28% lower than the control, was consistent with the results observed by the naked eye (Fig. 1b). Additionally, values for these five morphological indices increased from S1 to S4 in both control and EGTA-treated plants. The effects of EGTA treatment on photosynthetic characteristics were also investigated, and the photosynthesis rate (Pn) was significantly lower with EGTA treatment at S3 and S4 compared to the control (Fig. 1b). However, stomatal conductance (Gs) and transpiration rate (Tr) showed no significant differences between the control and the EGTA treatment. Additionally, the values for Pn and Tr increased from S1 to S3 and then decreased from S3 to S4 in the control and EGTA-treated plants, whereas Gs remained the same from S1 to S2, decreased from S2 to S3, and increased from S3 to S4.

### Cell wall-composition analysis

To address whether EGTA treatment altered the cell wall composition, *X*-ray photoelectron spectroscopy (XPS) and Fourier-transform infrared spectroscopy (FTIR) were performed. Peaks for carbon_1s_ (C_1s_), nitrogen_1s_ (N_1s_), oxygen_1s_ (O_1s_), and calcium_2p_ (Ca_2p_) were identified in both control and EGTA-treated cell walls (Supplementary Table S1). The relative proportion of N within the cell wall decreased as the developmental stage progressed, whereas the relative proportions of C and O within the cell wall remained constant. The relative proportion of Ca within the cell wall increased during development. With EGTA treatment, no significant difference was observed in the relative proportion of these cell wall elements, but the relative proportions of N and Ca within the cell wall were significantly lower in EGTA-treated plants compared to the controls.

**Fig. 2:**
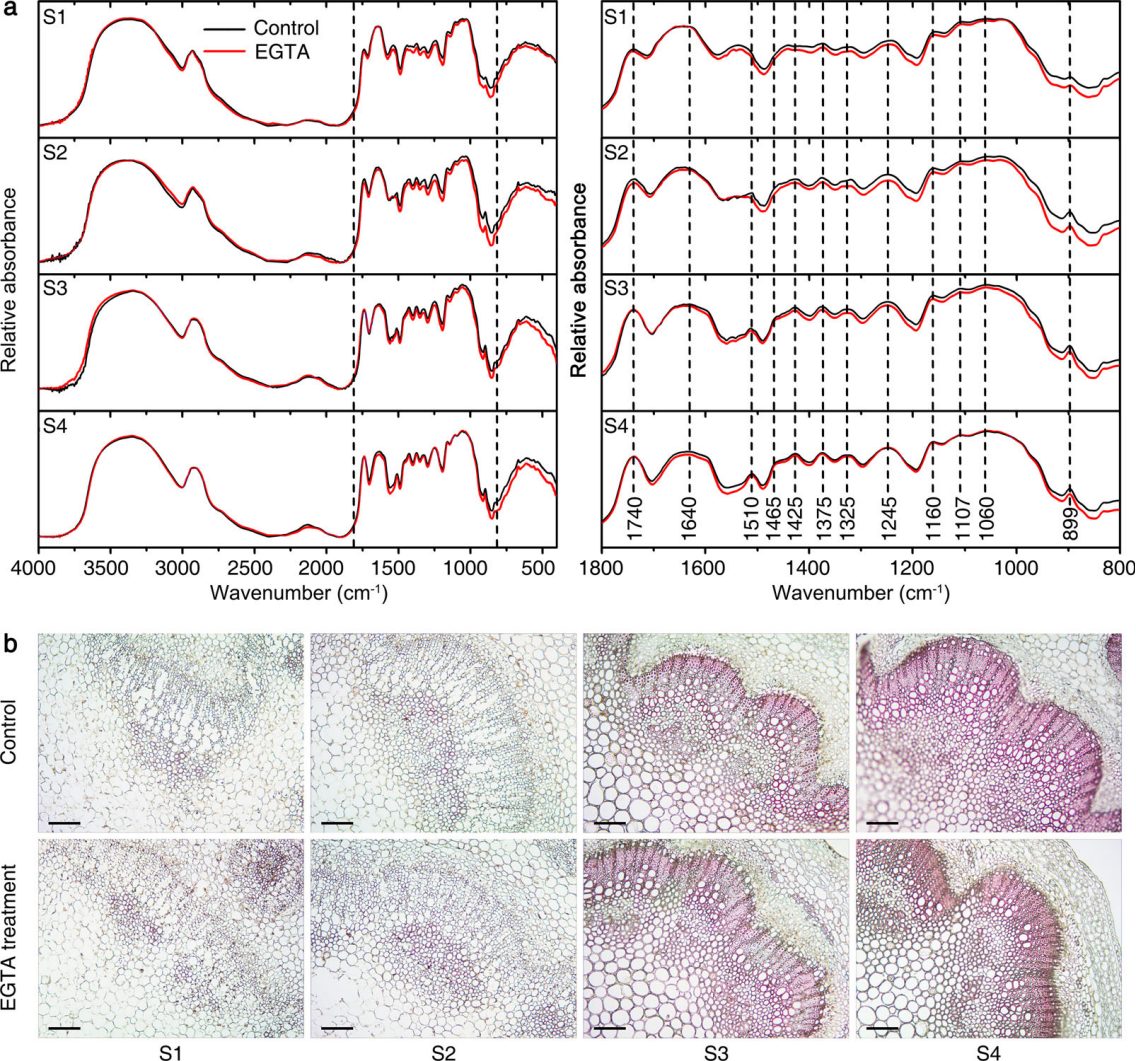
Effects of EGTA treatment on cell wall compositions of *P. lactiflora* inflorescence stems. **a** Absorption FTIR spectra of the cell wall in the 4000–400 cm^-1^ and 1800–800 cm^-1^ regions. **b** Transverse sections of 8 µm thickness were stained with phloroglucinol-HCl, which selectively stains lignified cell walls. Bars, 100μm. FTIR, Fourier-transform infrared spectroscopy; S1, flower-bud stage; S2, pigmented stage; S3, unfold-petal stage; S4, full-flowering stage

The characteristic spectra of the most cell wall chemical groups lie between 1800 and 800 cm^-1^. Functional groups were assigned to the spectral peaks as follows: 1740 (carbonyl C = O), 1640 (amide I C = O), 1510 (aromatic skeletal vibrations), 1465, 1425, 1325 (lignin), 1375 (CH band), 1245 (amide III in protein), and 1160, 1107, 1060 and 899 cm^-1^ (CHO)^21,22^. Although the FTIR spectra of cell walls in control and EGTA-treated plants were essentially similar, the relative absorbances of the peaks were different with EGTA treatment compared to the control (Fig. 2a). For example, the relative absorbances at 1325, 1425, 1465, 1510, and 1640cm^-1^, which correspond to lignin or lignin-like structures, were lower in EGTA-treated plants, as were the relative absorbances at 899, 1060, 1107, 1160, 1245, and 1375 cm^-1^, which correspond to polysaccharide-like cellulose structures.

Histochemical staining was used to verify the deposition of lignin on the secondary wall. Specifically, inflorescence stem transverse sections were stained with phloroglucinol-hydrochloric acid, which selectively stains lignified cell walls red, and were observed by light microscopy. The xylem cell walls at S1 and S2 did not stain red, whereas the secondary wall in the xylem, which contains high levels of lignin, stained red at S3 and S4 (Fig. 2b). The staining intensity was evaluated by comparing the brightness of the stain color. There was less secondary wall staining in EGTA-treated inflorescence stems at S3 and S4 compared to the control, but the difference was not significant. There was significantly less stained area in EGTA-treated inflorescence stems at S3 and S4 compared to the control stems.

### Microstructure observations

**Fig. 3:**
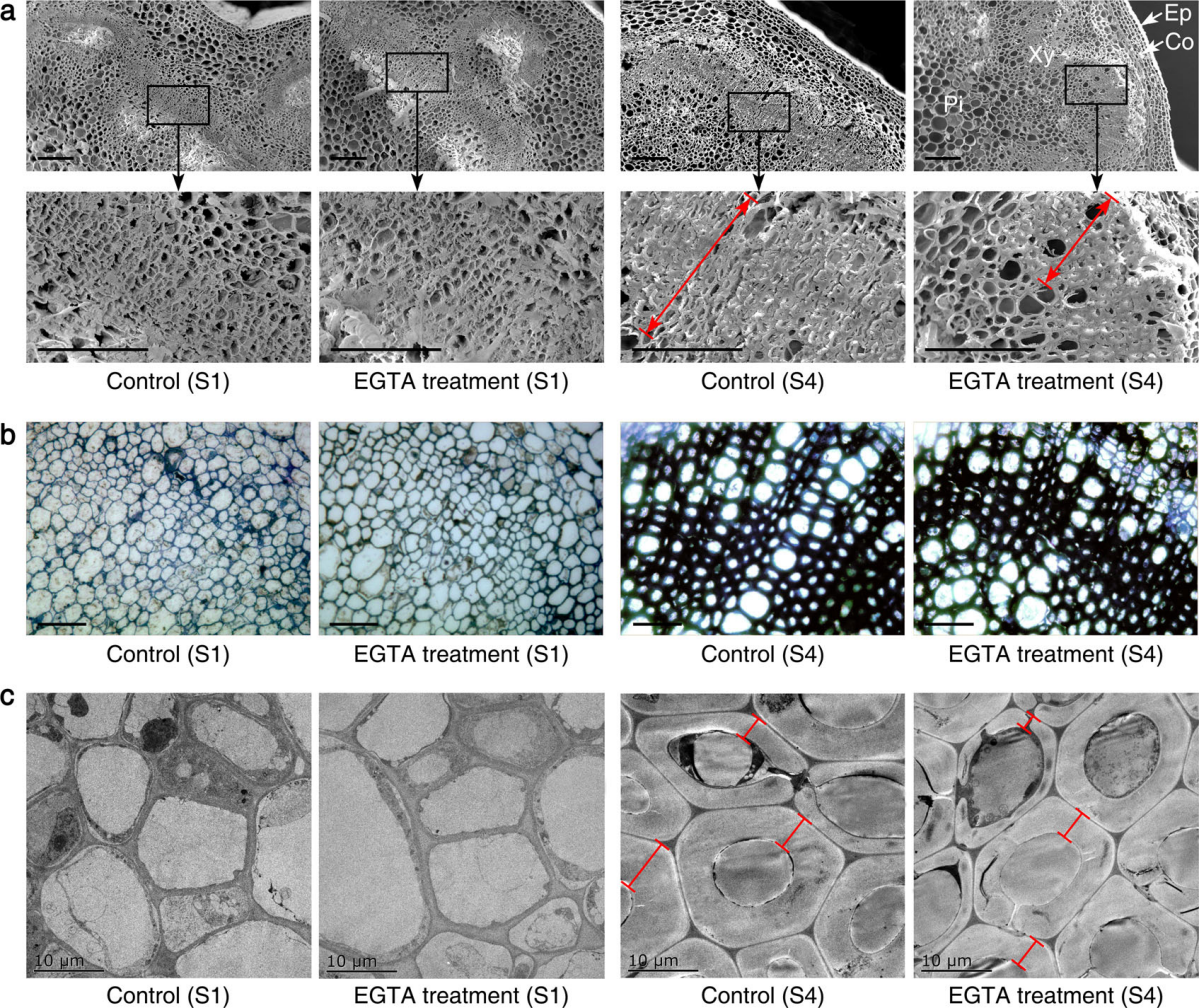
Effects of EGTA treatment on the microstructures of *P. lactiflora* inflorescence stems at S1 and S4. **a** Scanning electron microscopy. Micrographs of partial enlargement of the regions are marked by the arrow. Bars, 100μm. **b** Optical microscope. Bars, 50μm. **c** Transmission electron microscopy. Bars, 10μm. Ep, epidermis; Co, cortex; Xy, xylem; Pi, pith; S1, flower bud stage; S4, full flowering stage

We examined the inflorescence stem transverse sections by scanning electron microscopy (SEM). The walls in the xylem cells thickened from S2 to S4 (Fig. 3a). With EGTA treatment, there were significantly fewer xylem cells with thickened secondary walls at S4 compared to that in the same area of the untreated control. To verify the difference in the number of xylem cell layers with thickened secondary walls in inflorescence stems after EGTA treatment, transverse sections were observed under an optical microscope, which revealed the same result (Fig. 3b). The alteration of the thickened secondary walls in xylem cells was further analyzed by transmission electron microscopy (TEM). TEM verified the secondary wall formation in the xylem cells during flower-development stages (Fig. 3c). EGTA treatment caused fewer thickened secondary walls in the xylem cells at S4 compared to the control, but this difference was not significant.

### Differentially expressed proteins detected by iTRAQ

To investigate the underlying molecular mechanisms in response to EGTA treatment, control and EGTA-treated inflorescence stems at S4 were sampled for proteomic analysis using isobaric tags for relative and absolute quantitation (iTRAQ). As a result, 256,853 spectra with 19,326 matching known peptides were obtained, and 5045 proteins were identified. Under EGTA treatment, 1065 differentially expressed proteins (DEPs) compared to the control were found. Of these, 533 were upregulated and 532 were downregulated. To further explore the functions of these DEPs, gene ontology (GO) and Kyoto encyclopedia of genes and genomes (KEGG) enrichment analyses were implemented.

**Fig. 4:**
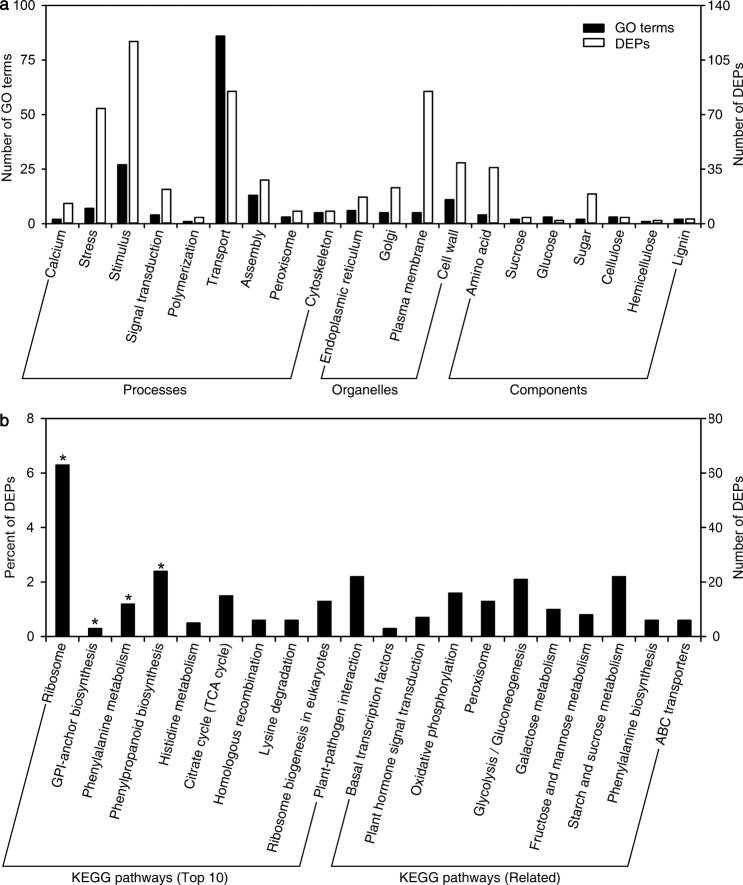
Effects of EGTA treatment on the proteome of *P. lactiflora* inflorescence stems. **a** GO enrichment analysis of differentially expressed proteins related to mechanical strength-related processes. **b** Top 10 KEGG pathways and 10 KEGG enrichment analyses of differentially expressed proteins related to mechanical strength-related processes. *Differentially expressed proteins and significantly enriched pathways. DEPs, differentially expressed proteins; GO, gene ontology; KEGG, Kyoto encyclopedia of genes and genomes

Of the 1065 DEPs, 554 were annotated via GO analysis. A total of 1186 GO terms were searched, of which 746 were involved in biological processes, 143 in cellular components, and 297 in molecular function categories (Supplementary Table S2A). Twenty keywords encompassed the most important processes that responded to EGTA (Ca, stress, stimulus, signal transduction, polymerization, transport, assembly, peroxisome, and cytoskeleton) and organelles and components related to inflorescence stem mechanical strength (endoplasmic reticulum, golgi, plasma membrane, cell wall, amino acid, sucrose, glucose, sugar, lignin, cellulose, and hemicellulose). A total of 192 GO terms and 314 DEPs were screened (Fig. 4a and Supplementary Tables S2B and S2C). Most GO terms were related to transport, stimulus, assembly, and cell wall, and these DEPs covered various functions. For example, cinnamyl-alcohol dehydrogenase (CAD) is involved in lignin biosynthesis, calcium-binding protein (CML) is a major Ca^2+^ sensor involved in Ca^2+^ signature transduction, and ATP-binding cassette, subfamily G, member 2 (ABCG2) plays an important role in transport.

Through KEGG analysis, 837 DEPs were annotated and assigned to 121 KEGG pathways. These DEPs were significantly enriched (*P*<0.05) in four pathways, including ribosome (63 DEPs), glycosylphosphatidylinositol (GPI)-anchor biosynthesis (3 DEPs), phenylalanine metabolism (12 DEPs), and phenylpropanoid biosynthesis (24 DEPs; Fig. 4b). Among these four KEGG pathways, phenylalanine metabolism and phenylpropanoid biosynthesis were related to inflorescence stem mechanical strength processes. In the remaining 10 KEGG pathways, the top two were plant–pathogen interactions (22 DEPs) and the citrate cycle (TCA cycle; 15 DEPs; Fig. 4b), which were also related to inflorescence stem mechanical strength processes. Apart from these pathways, another 10 KEGG pathways that might be involved in inflorescence stem mechanical strength processes, including starch and sucrose metabolism, were also found. Abundant DEPs were significantly enriched for starch and sucrose metabolism (22 DEPs) and glycolysis/gluconeogenesis (21 DEPs; Fig. 4). As a result, 144 DEPs were enriched in these KEGG pathways after EGTA treatment. Of them, 80 were upregulated and 66 were downregulated (Supplementary Table S3).

**Fig. 5:**
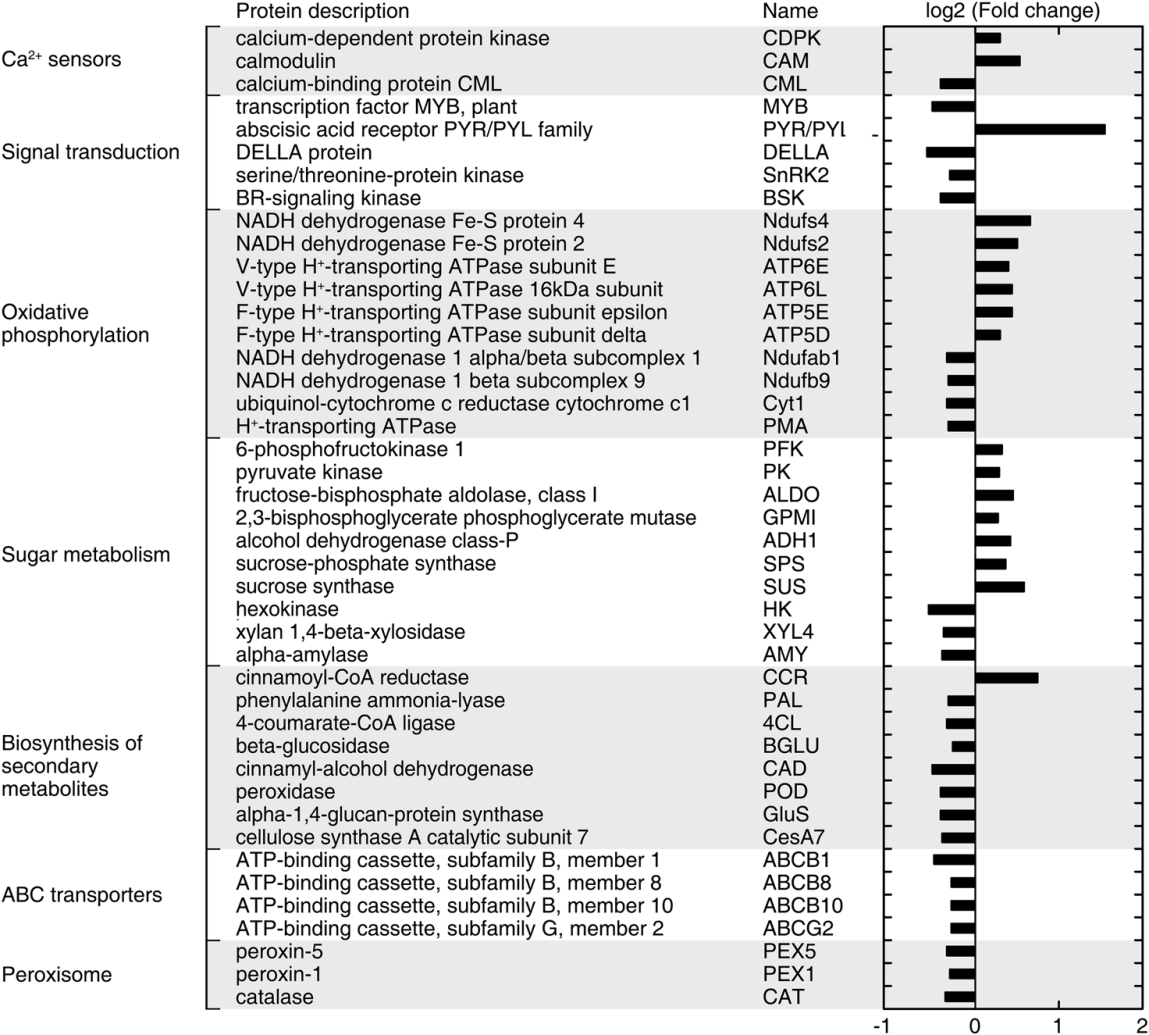
The log2 relative expression levels of 43 overlapped differentially expressed proteins from KEGG and GO analysis. GO, gene ontology; KEGG, Kyoto encyclopedia of genes and genomes

In addition, we found 43 DEPs (17 upregulated and 26 downregulated) from the overlapping KEGG and GO analysis results (Fig. 5 and Supplementary Table S4). These DEPs were related to Ca^2+^ sensors, signal transduction, oxidative phosphorylation, sugar metabolism, biosynthesis of secondary metabolites, ABC transporters, and peroxisomes. Among the upregulated DEPs, a more than 1.5-fold elevation of the abscisic acid receptor PYR/PYL family (PYR/PYL), which inhibits the signal transduction of abscisic acid, was observed. The remainder of the upregulated DEPs, such as NADH dehydrogenase Fe-S protein (Ndufs), V/T-type H^+^-transporting ATPase, and 6-phosphofructokinase 1 (PFK), were involved in oxidative phosphorylation and sugar metabolism. Of the downregulated proteins, DELLA protein, which acts as a negative regulator of gibberellin biosynthesis, was most significantly suppressed by EGTA treatment. CAD, peroxidase (POD), and ATP-binding cassette (ABC) enzymes were involved in lignin biosynthesis, and cellulose synthase A catalytic subunit 7 (CESA7), which is involved in cellulose biosynthesis, was also inhibited by EGTA treatment. Additionally, the Ca^2+^ sensor CML and MYB transcription factor were also suppressed by EGTA treatment.

### Quantitative real-time polymerase chain reaction analysis

Ca^2+^-binding proteins related to secondary wall biosynthesis were identified through proteomic analyses. To characterize the effect of EGTA treatment on Ca^2+^ binding and secondary wall biosynthesis in inflorescence stems, we examined the expression of genes corresponding to these proteins by quantitative real-time polymerase chain reaction (qRT-PCR). These genes included calmodulin (*CaM*)/*CML* and calcium-dependent protein kinase (*CDPK*), which are involved in Ca^2+^ binding, and *an MYB* gene that acts as a transcription factor regulating secondary wall biosynthesis. Additionally, phenylalanine ammonia-lyase gene (*PAL*), 4-coumarate-CoA ligase (*4CL*), and *CAD*, which are involved in the phenylpropanoid pathway and important for monolignol synthesis, were analyzed, as well as *POD* and *ABC* genes (such as *ABCC2* and *ABCG2*), which are related to monolignol transport. We also analyzed the expression of calcineurin B-like protein-interacting protein kinase (*CIPK*), cinnamate 4-hydroxylase (*C4H*), cinnamoyl-CoA reductase (*CCR*), caffeoyl shikimate esterase (*CSE*), caffeic acid 3-*O*-methyltransferase (*COMT*), and caffeoyl-CoA 3-*O*-methyltransferase (*CCoAOMT*), which are related to Ca^2+^-binding proteins and secondary wall biosynthesis.

**Fig. 6:**
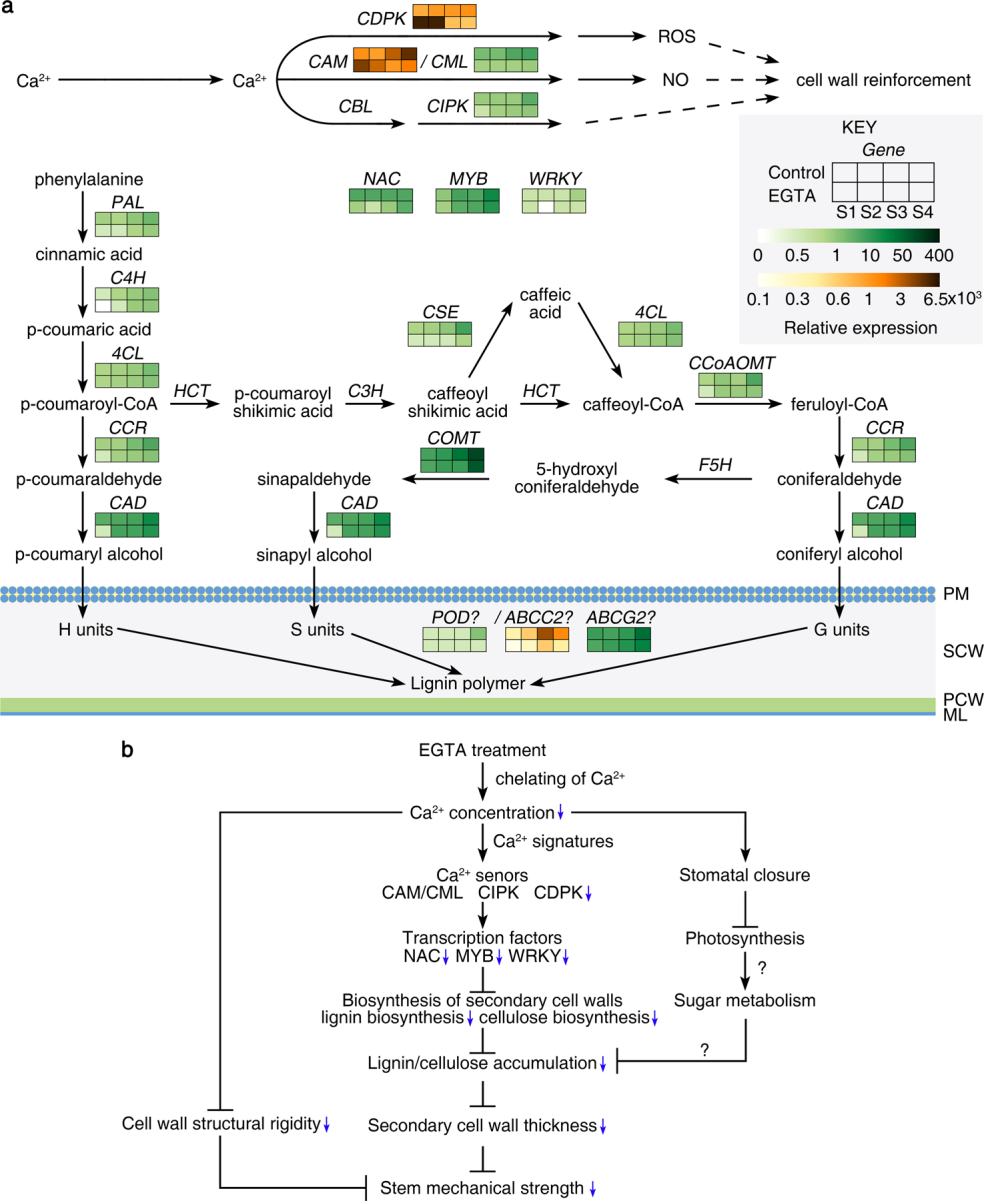
Effects of EGTA treatment on key genes related to mechanical strength and a proposed pathway for EGTA-mediated reduction of mechanical strength of *P. lactiflora* inflorescence stems. **a** Heat maps of the expression of 23 genes involved in Ca signal transduction and secondary cell wall biosynthesis at four developmental stages. PM, plasma membrane; SCW, secondary cell wall; PCW, primary cell wall; ML, middle lamella. **b** Proposed pathway for EGTA-mediated weakness of the mechanical strength of *Paeonia lactiflora* inflorescence stems. ML, middle lamella; PCW, primary cell wall; PM, plasma membrane; SCW, secondary cell wall; S1, flower-bud stage; S2, pigmented stage; S3, unfold-petal stage; S4, full-flowering stage

*CDPK*, *CAM*, and *ABCC2* were expressed at the highest levels, whereas *PAL*, *C4H*, *4CL,* and *POD* were expressed at the lowest levels (Fig. 6a). With EGTA treatment, the expression levels of *CML*, *CIPK*, *NAC*, *MYB*, *PAL*, *CCR*, *CSE*, *COMT*, and *ABCC2* were lower by about 79, 49, 77, 39, 65, 48, 84, 49, and 69%, respectively, compared to the untreated control. Lower expression levels of *WRKY*, *4CL*, *CCoAOMT*, and *POD* were also observed in response to EGTA treatment at the S4 stage of development. The expression levels of the *C4H* and *ABCG2* genes were lower by about 88, 25% and 73, 28% compared to the control at S1 and S4, respectively. The expression levels of the *CDPK* and *CAM* genes were significantly lower in EGTA-treated inflorescence stems at S3 and S4. In contrast, the expression of these two genes increased in EGTA-treated inflorescence stems at S1 and S2. For* CAD*, there was no significant difference in expression between the control and EGTA-treated plants. EGTA stimulated the expression of genes involved in Ca^2+^ signal transduction and lignin synthesis.

## Discussion

Ca^2+^ deficiency is very common in nature and can severely affect plant growth, development, and metabolism^1^. However, the molecular mechanisms of Ca^2+^ deficiency in *P. lactiflora* are unknown. In this study, we found that Ca^2+^ deficiency in *P. lactiflora* significantly inhibited growth and development, especially for the upper inflorescence stems. EGTA reduced Ca^2+^ in the cell wall, lignin deposition, and the number of xylem cell layers with thickened secondary walls. Also, we used iTRAQ to analyze the DEPs in EGTA-treated inflorescence stems at S4. Ca^2+^ deprivation induced a signal-like stress, which was decoded by Ca^2+^ sensors, inducing downstream physiological responses. Simultaneously, sugar and energy metabolism and secondary metabolites were affected by the lack of Ca^2+^. Furthermore, qRT-PCR analyses verified that Ca^2+^ deprivation reduced the biosynthesis and accumulation of the secondary metabolites used to form lignin.

In ornamental plants, morphological characteristics, especially inflorescence stem mechanical strength, play crucial roles in the quality of cut flowers. Inflorescence stem mechanical strength is correlated with internode number, stem length, weight, and diameter^21,23^. In *P. lactiflora*, there is a positive correlation between inflorescence stem mechanical strength and stem weight and diameter^12^. In our study, EGTA treatment led to a comprehensive inhibition of *P. lactiflora* plant development, reducing the weight, diameter, and mechanical strength of inflorescence stems. Mechanical support of plants is mainly contributed by secondary walls^13,16^, and stem mechanical strength is related to the thickness of the secondary walls. For example, Aohara^24^ et al. reported that the thickness of secondary walls of *O. sativa* under the epidermal layer in rice *brittle culm 5* (brittle node) mutants is notably thinner than those in wild-type nodes, and similar results were found in the *flexible culm 1* mutants^16^. In our study, similar results were found for secondary walls, which were thinner with EGTA treatment compared to the control. The thinner thickened secondary walls distributed around the xylem are important for stem mechanical strength in *P. lactiflora*, but the thickened secondary walls of cells under the epidermal layer are not. Interestingly, we found a nearly 50% decrease in the number of xylem cells in thickened secondary walls after EGTA treatment compared to controls. The reduction in xylem cells in thickened secondary walls (reduction of area with thickened secondary walls) was more important than the reduction of these thickened secondary walls in causing loss of mechanical strength.

Secondary walls are composed mainly of the polysaccharides cellulose and hemicellulose, as well as lignin and cell wall proteins. Mechanically, secondary walls can be compared to reinforced concrete, with the cellulose microfibrils that are embedded in an amorphous matrix of lignin 25 being analogous to steel rods . Therefore, stem mechanical strength is determined mainly by the properties of the secondary walls, especially lignin^26^. Wang et al.^21^ reported that among the three genotypes of wheat (*Triticum aestivum* L.), the genotype with the lowest bending stress contained the least lignin. In *O. sativa*, decreased accumulation of lignin was also observed in *brittle culm 5* nodes^24^. In ornamental plants, similar results have been reported for *G. jamesonii*^14^ and *P. lactiflora*^8^. In our study, we also found less lignin accumulation in EGTA-treated plants compared to controls. The weakened inflorescence stems were primarily due to the decreased lignin accumulation induced by EGTA treatment.

The transcriptional regulation of secondary wall deposition is a multi-leveled, hierarchical network, mainly regulated by *NAC* and *MYB* transcription factors. These regulators together activate downstream transcription factors and secondary wall biosynthetic genes^27^. In this study, both the *NAC* and *MYB* transcription factors had lower expression in response to EGTA treatment compared to the control. *NAC* transcription factors are the master switches that activate secondary wall biosynthesis in vessels and fibers^28^. The *MYB46* and *MYB83* transcription factors are second-level master switches that activate the expression of many lignin biosynthetic genes^29^. The suppression of *NAC* and *MYB* transcription factors by EGTA treatment affected the expression of downstream transcription factors and secondary wall biosynthetic genes and eventually influenced secondary wall deposition. However, the functions of *NAC* and *MYB* in this study were not conclusive, and additional studies are needed.

Lignin is a phenolic component of cell walls that plays a role in maintaining cell wall rigidity and can be categorized as hydroxyphenyl (H), guaiacyl (G), or syringyl (S) lignin^30,31^. The lignin monomer-biosynthesis pathway is well established, and several genes for lignin biosynthesis have been identified, including *PAL*, *C4H*, *4CL*, *CCR*, *CAD*, *p*-hydroxycinnamoyl-CoA:quinate/shikimate *p*-hydroxycinnamoyltransferase (*HCT*), *p*-coumarate 3-hydroxylase (*C3H*), *CSE*, ferulate 5-hydroxylase (*F5H*), *COMT,* and *CCoAOMT* (Fig. 6a)^25,32^. In our study, the expression of *PAL*, *C4H*, *4CL*, *CCR*, *CAD*, *CSE*, *COMT*, and *CCoAOMT*, which are involved in lignin biosynthesis, was examined by qRT-PCR. They all had lower expression levels in EGTA-treated inflorescence stems, especially *PAL*, *CSE*, and *COMT*, compared to the control. Twenty-four DEPs were significantly enriched in phenylpropanoid biosynthesis, and PAL, 4CL, and CAD were all expressed at lower levels in EGTA-treated inflorescence stems compared to the control. These genes are related to lignin accumulation. Lignin in stem cell walls of *PAL*-knockdown plants in *Brachypodium distachyon* is reduced by approximately 43%^33^. Downregulation of *C4H* in hybrid eucalyptus (*Eucalyptus urophylla* × *Eucalyptus grandis*)^34^, *4CL* in hybrid poplar (*Populus tremula* × *Populus alba*)^35^, *CCR* in birch (*Betula platyphylla* × *Betula pendula*)^36^, *CAD* in *Arabidopsis thaliana*^37^, and *a CCoAOMT* homolog in tobacco (*Nicotiana tabacum*)^38^ leads to varying degrees of reduction in lignin content compared to controls. The suppression of these genes also affects plant development, especially the repression of *CCR*, which reduces the thickness of secondary walls. The expression level of *COMT* is lower in *Chlorogalum grandiflorum* varieties with a poor vase life, which is consistent with reduced lignin content and pedicel rigidity^39^. The *CSE*-2 loss-of-function mutant shows the typical phenotype of lignin-deficient mutants^40^. Low expression of lignin biosynthesis-related genes in EGTA-treated inflorescence stems caused a reduction in lignin accumulation.

EGTA chelates cell wall-associated Ca^2+^, and it does not cross the cell membrane^41^. In our study, the concentration of Ca in inflorescence stem cell walls was lower in plants treated with EGTA, indicating that EGTA chelated Ca^2+^ in the cell wall. Ca^2+^ binds with pectin and plays an important role in cell wall structural rigidity. Chelation of cell wall-associated Ca^2+^ reduced the Ca^2+^ concentration in cell walls and caused a low Ca^2+^ environment in the cytoplasm. We surmised that like stimuli such as mechanical injury, cold or ABA, which induce spontaneous Ca^2+^ oscillations, EGTA treatment may also induce Ca^2+^ oscillations and generate a Ca^2+^ signature. Plants have a unique repertoire of Ca^2+^-binding proteins to decode Ca^2+^ signatures in the gene families of CAMs/CMLs, CDPKs, and CIPKs^42,43^. These proteins sense the Ca^2+^ signature and activate downstream transcription factor activity and gene expression to alter biochemical function through reversible phosphorylation^7^. We found that the expression levels of proteins involved in oxidative phosphorylation changed in response to EGTA treatment. The expression of H^+^-transporting ATPases and several NADH dehydrogenase enzymes was upregulated, whereas the expression of NADH dehydrogenase Fe-S proteins and V/F-type H^+^-transporting ATPases was downregulated, which suggested that EGTA application affected the phosphorylation of proteins involved in the TCA cycle and glycolysis in inflorescence stems and was also related to signal transduction. *CDPK*, a Ca^2+^ sensor, plays an important role in plant cell wall development and secondary growth. For example, Zhao et al.^44^ found a major role for *CDPK11*/*24* function in plant pollen-tube growth in double mutant lines, and Matschi et al.^45^ reported that secondary growth is inhibited by a single *CDPK* (*AtCPK28*) loss-of-function mutant in *Arabidopsis*. CAM and CML, members of the NAC, MYB, and WRKY TF families, regulate secondary wall biosynthesis and interact with Ca^2+^/CaM^25,46^. In our study, the expression levels of these Ca^2+^ signal sensors were different in treated plants compared to controls, suggesting that EGTA application generated Ca^2+^ signatures that were sensed by CDPK, CAM/CML, and CIPK. The differences in the expression levels of these proteins influenced downstream secondary wall development and thereby reduced the inflorescence stem mechanical strength in *P. lactiflora*.

Ca^2+^ deprivation inhibits photosynthesis by reducing water absorbance and regulation, resulting in leaf wilting and closure of the stomata on the leaves. This prevents gas exchange and leads to a reduction in the cellular carbon dioxide (CO_2_) concentration and concomitantly rubisco carboxylase activity^19^. Pn, Tr, and Gs decreased in Japanese morning glory (*Pharbitis nil* Chois., cv. Violet) after EGTA treatment^47^, which is consistent with the results of our study. Therefore, Ca^2+^ plays an important role in photosynthesis, and Ca^2+^ deprivation would suppress photosynthesis in leaves. The reduction in photosynthesis would cause the yield of photosynthetic products available for secondary wall biosynthesis to also be lower. In this study, the expression levels of proteins functioning in sugar metabolism were upregulated, whereas those of proteins that function in decomposition were downregulated. This result suggested that the reduced dry-matter accumulation was due to the lower rates of photosynthesis and altered sugar metabolism, which affected the synthesis of secondary walls.

## Conclusion

The application of EGTA, a Ca^2+^ chelator, caused Ca^2+^ deprivation in *P. lactiflora* and inhibited plant development, including the mechanical strength of inflorescence stems. We have put forward a hypothetical mechanism by which EGTA application reduced the mechanical strength of inflorescence stems (Fig. 6b). Ca^2+^ plays an important role in determining the structural rigidity of the cell wall. The application of EGTA chelated Ca^2+^ in the cell wall and made it more pliable. The low Ca^2+^ in response to EGTA application induced spontaneous Ca^2+^ oscillation and generated Ca^2+^ signatures. The Ca^2+^ signatures were sensed by Ca^2+^-binding proteins, which were activated through protein phosphorylation. Via the activities of the *NAC*, *MYB* and *WRKY* TF families, the expression of genes involved in lignin and cellulose biosynthesis was reduced. Lignin and cellulose are both main components of secondary walls, and the reduction of these components affected the development and thickness of the secondary walls. In turn, this decreased the mechanical support of the inflorescence stems. Furthermore, the Ca^2+^ signatures also induced the closure of the stomata and reduced the rates of photosynthesis. The reduction in photosynthetic products affected sugar metabolism and resulted in a decrease in metabolites available for the formation of secondary walls.

## Materials and methods

### Plant materials and treatment

*P. lactiflora* cv. ‘Hongyan Zhenghui’, grown in the field of the germplasm repository of Yangzhou University, Jiangsu Province, China (32°23′ N, 119°24′ E), was used as the experimental material. Specimens were planted in 60 rows with two plants per row in the field and divided into two groups with 30 rows per group. One group was sprayed with 10 mmolL^-1^ EGTA, and the other was sprayed with distilled water as a control. Treatments were done once a week from the leaf-expansion stage on 30 March to the bloom stage, with a total of five applications. During the development of *P. lactiflora*, morphological indices and photosynthetic characteristics were measured every 8 days from 7 April to 1 May, including four developmental stages (S1 to S4), and the upper parts (12 cm) of thirty inflorescence stems were collected as samples. Subsequently, 5- to 8-cm lengths of the upper part of inflorescence stems were cut into 5-mm sections and fixed with 2.5% glutaraldehyde (GA) solution in 0.1 molL^-1^ potassium phosphate buffer solution (PBS; pH 7.8). Other samples were fixed with formalin-aceto-alcohol solution for microstructure observation. The remainder of the inflorescence stem samples was immediately frozen in liquid nitrogen and stored at -80 °C until examination.

### Morphological indices and photosynthetic characteristics

Morphological indices were measured as described previously^12^. Fully expanded leaves at the fourth apical node were selected to measure photosynthetic characteristics, including Pn, Tr, and Gs, using a portable photosynthesis system (LI-6400; Li-Cor, USA) between 8:00 and 10:00 am, before each collection.

### Cell wall-composition analysis

Cell wall materials were extracted from inflorescence stems as described previously^48^. *X*-ray photoelectron spectroscopy determination of cell wall elements was performed on a spectrometer (ESCALAB250Xi; Thermofisher Scientific, USA) using a set monochromatic radiation A1Kα source with a background pressure of 5 × 10^-10 ^mbar. Each analysis consisted of a wide survey scan (pass energy 100 eV, 1.0 eV step size) FTIR determination of cell wall materials and was analyzed by an infrared spectroscopy (IR) spectrometer (670-IR + 610-IR; Varian, USA) using the KBr pellet technique. Spectra were taken in the wave number range from 4000 to 400 cm^-1^ with a resolution of 4 cm^−1^ and 32 scans per sample. All spectra were normalized and baseline-corrected with OPUS management software.

### Histological staining

Stem sections were fixed with formalin-aceto-alcohol for 2 h at room temperature and at 4 °C for a minimum of 1 day. Afterwards, the samples were dehydrated in a graded ethanol series, dehydrated with a xylene–ethanol mixture solution (1:3, 1:1, 3:1; v/v), and finally dehydrated with a xylene–chloroform mixture (9:1; v/v) twice. After infiltration and embedding in paraffin, 8 μm sections were cut using a rotary microtome (RM2245; Leica, USA) and mounted on glass slides. After the paraffin was removed with xylene, sections were rehydrated in a graded ethanol series followed by distilled water and finally air dried. To detect lignin, the sections were stained with 2% (w/v) phloroglucinol (Sigma, China) for 2 min and then treated with 18% hydrochloric acid. The stained sections were observed immediately under a light microscope (CX31RTSF; Olympus, Japan). For cellulose, the sections were stained with 0.1% calcofluor white M2R in 0.05% Evans blue (18909; Sigma, China) for 2 min and observed under a fluorescence microscope with a UV excitation filter SZX2-FUV, a GFP high-performance filter SZX2-FGFPHQ, and an RFP filter SZX2-RFP1 for calcofluor staining (CX31RTSF; Olympus, Japan).

### Microstructure observation

Inflorescence stem sections were fixed with 2.5% GA in 0.1 molL^-1^ PBS (pH 7.8) for 2 h at room temperature and at 4 °C for more than 1 day. They were rinsed with the same buffer three times (15 min each). For SEM observation, the samples were dehydrated and critical-point dried, sputter-coated with gold, and observed with an SEM (XL-30 ESEM; Philips, Holland) under 5 kV. For TEM observation, the samples were post-fixed in 1% osmium tetroxide in 0.1 molL^-1^ PBS (pH 7.8) for 4 h at 4 °C after GA. After dehydration in an ethanol series, the samples were infiltrated and embedded in butyl-methyl methacrylate. Seventy-nanometer sections were cut, stained with uranyl acetate and lead citrate, and viewed with a TEM (CM100; Philips, Holland).

### Proteome with iTRAQ analysis

Samples at S4 were used to perform proteome analyses. Protein extraction, quantification, iTRAQ labeling and strong cation-exchange fractionation, LC-ESI-MS/MS analysis, database search, quantification bioinformatics, and annotations were performed as described previously^49^, with a few modifications. Briefly, the extraction and purification of protein followed the manufacturer’s instructions. The control samples were labeled with iTRAQ tags 114, 115N, and 115C, and the EGTA-treated samples were labeled with 117C, 118N, and 118C. The combined peptide mixtures were lyophilized. After trypsin digestion, the peptides were desalted on a C18 column (Phenomenex) and dried in a spin vacuum. The desalted peptides (100 μg) were labeled with iTRAQ 6-plex reagent in 200 mmolL^-1^ triethylamonium bicarbonat (TEAB) according to the manufacturer’s instructions. The peptides labeled with different reagents were combined, vacuum-dried, and separated on an LC-20AB HPLC Pump system (Shimadzu, Japan) coupled with a high-pH RP column. Subsequently, the peptides were injected into the tandem mass spectrometer Q EXACTIVE (Thermofisher Scientific, USA) for data-dependent acquisition detection by nano-electrospray ionization.

Raw data were processed with OpenMS software and searched against the SwissProt and common MS contaminant database using Mascot software. Trypsin was chosen as the enzyme with one missed cleavage allowed. The fixed modification carbamidomethylation was set for cysteine and the variable modification oxidation for methionine. The peptide tolerance was set as 0.05 Da, and the MS/MS tolerance was set as 0.1 Da. The ratio of each protein is given by the geometric mean of the protein ratios measured from all replicates. Student’s *t* test was used to analyze the significance of protein geometric ratios, and the Benjamini-Hochberg multiple hypothesis test correction was employed to correct the *P* values. DEPs were screened out using 1.2 normalized protein geometric ratios at *a P* < 0.05 level with 5% false discovery rate (FDR) correction.

### qRT-PCR analyses

Gene transcript levels were analyzed using qRT-PCR with a BIO-RAD CFX ConnectTM Optics Module (Bio-Rad, USA). cDNA was synthesized from RNA using the PrimeScript® RT Reagent Kit With gDNA Eraser (TaKaRa, Japan). All gene-specific primers in this study are shown in Supplementary Table S5. qRT-PCR was performed using SYBR® Premix Ex TaqTM (Perfect Real Time) (TaKaRa, Japan). The amplification was carried out under the following conditions: 55 °C for 2 min, followed by an initial denaturation step at 95 °C for 30 s, and 40 cycles at 95 °C for 5 s, 55 °C for 15 s, and 72 °C for 30 s. Relative expression levels of target genes were calculated by the 2^-△△Ct^ comparative threshold cycle method^50^.

### Statistical analysis

All experiments described here were repeated three times in a completely randomized design. Primers were designed using the Primer 5.0 program. All data were means of three replicates with standard deviations. The results were analyzed for variance using the SAS/STAT statistical analysis package (version 6.12; SAS Institute, Cary, NC, USA).

## Supplementary information

Supplementary Table S1-S5
