# Supplementary material for: EGTA reduces the inflorescence stem mechanical strength of herbaceous peony by modifying secondary wall biosynthesis
Source: Hortic Res. 2019 Mar 1;6:36. doi: 10.1038/s41438-019-0117-7 (PMC6395589; doi:10.1038/s41438-019-0117-7)
Supplement: Supplementary file 1 — Supplementary Table S1-S5 [file 41438_2019_117_MOESM1_ESM.docx]

Title: EGTA, a calcium chelator, weakens mechanical strength of inflorescence stems of herbaceous peony (*Paeonia lactiflora* Pall.) through modifying secondary cell wall biosynthesis

**Authors:** Yuhan Tang, Daqiu Zhao, Jiasong Meng, Jun Tao

**Supplementary data**

Supplementary data are available at HR online.

Table S1. Supplementary data of elemental composition of cell walls in *Paeonia lactiflora* inflorescence stems using XPS.

Table S2A. Supplementary data of GO terms enrichment.

Table S2B. Supplementary data of GO terms involved in twenty types.

Table S2C. Supplementary data of 314 DEPs description.

Table S3. Supplementary data of 146 DEPs description.

Table S4. Supplementary data of 43 DEPs description.

Table S5. Supplementary data of gene-specific primers used in qRT-PCR analysis.

**Supplementary Table S1.** Supplementary data of elemental composition of cell walls in *Paeonia lactiflora* inflorescence stems using XPS.

| **Stage** | **Treatment** | **Element (%)** | | | |
| --- | --- | --- | --- | --- | --- |
|  |  | **C** | **O** | **N** | **Ca** |
| S1 | Control | 60.73±0.38^bc^ | 33.01±0.21^c^ | 5.90±0.11^a^ | 0.36±0.05^e^ |
|  | EGTA | 60.13±0.13^c^ | 34.10±0.84^ab^ | 5.47±0.44^b^ | 0.31±0.02^f^ |
| S2 | Control | 61.09±1.13^bc^ | 34.31±1.07^ab^ | 4.18±0.35^c^ | 0.42±0.01^d^ |
|  | EGTA | 61.89±2.42^ab^ | 33.84±0.91^bc^ | 3.91±0.09^d^ | 0.36±0.04^e^ |
| S3 | Control | 61.67±1.54^abc^ | 34.46±1.26^ab^ | 3.36±0.30^e^ | 0.52±0.03^b^ |
|  | EGTA | 62.11±0.82^ab^ | 34.41±1.20^ab^ | 3.03±0.11^f^ | 0.45±0.06^cd^ |
| S4 | Control | 61.83±2.23^ab^ | 35.00±1.94^a^ | 2.62±0.26^g^ | 0.56±0.06^a^ |
|  | EGTA | 63.21±0.98^a^ | 34.03±0.76^b^ | 2.30±0.21^h^ | 0.47±0.06^c^ |

Atomic ratios (%) are obtained from the low-resolution survey scan. Values represent means ± SDs. Different letters indicate significant differences (*P* < 0.05)

**Supplementary Table S2A.** Supplementary data of GO terms enrichment.

| Categories | Gene Ontology ID | Gene Ontology term | Numbers of GO terms in the list | Numbers of GO terms in all the protein | P value |
| --- | --- | --- | --- | --- | --- |
| Cellular component | GO:0043228 | non-membrane-bounded organelle | 95 of 377 in the list | 323 of 1895 in all the Protein | 4.05E-06 |
| Cellular component | GO:0043232 | intracellular non-membrane-bounded organelle | 95 of 377 in the list | 323 of 1895 in all the Protein | 4.05E-06 |
| Cellular component | GO:0000786 | nucleosome | 19 of 377 in the list | 38 of 1895 in all the Protein | 2.60E-05 |
| Cellular component | GO:0032993 | protein-DNA complex | 19 of 377 in the list | 38 of 1895 in all the Protein | 2.60E-05 |
| Cellular component | GO:0005694 | chromosome | 24 of 377 in the list | 54 of 1895 in all the Protein | 2.81E-05 |
| Cellular component | GO:0000785 | chromatin | 19 of 377 in the list | 40 of 1895 in all the Protein | 6.48E-05 |
| Cellular component | GO:0044427 | chromosomal part | 19 of 377 in the list | 41 of 1895 in all the Protein | 9.90E-05 |
| Cellular component | GO:0031974 | membrane-enclosed lumen | 47 of 377 in the list | 146 of 1895 in all the Protein | 0.00017165 |
| Cellular component | GO:0005730 | nucleolus | 42 of 377 in the list | 129 of 1895 in all the Protein | 0.000296869 |
| Cellular component | GO:0043233 | organelle lumen | 46 of 377 in the list | 145 of 1895 in all the Protein | 0.000298104 |
| Cellular component | GO:0070013 | intracellular organelle lumen | 46 of 377 in the list | 145 of 1895 in all the Protein | 0.000298104 |
| Cellular component | GO:0031981 | nuclear lumen | 43 of 377 in the list | 135 of 1895 in all the Protein | 0.000432086 |
| Cellular component | GO:0044428 | nuclear part | 47 of 377 in the list | 152 of 1895 in all the Protein | 0.000497643 |
| Cellular component | GO:0005840 | ribosome | 55 of 377 in the list | 186 of 1895 in all the Protein | 0.000572392 |
| Cellular component | GO:0005634 | nucleus | 93 of 377 in the list | 357 of 1895 in all the Protein | 0.001002042 |
| Cellular component | GO:0044391 | ribosomal subunit | 30 of 377 in the list | 89 of 1895 in all the Protein | 0.001202306 |
| Cellular component | GO:0030529 | ribonucleoprotein complex | 61 of 377 in the list | 221 of 1895 in all the Protein | 0.00202572 |
| Cellular component | GO:0022627 | cytosolic small ribosomal subunit | 12 of 377 in the list | 26 of 1895 in all the Protein | 0.002078455 |
| Cellular component | GO:0022626 | cytosolic ribosome | 34 of 377 in the list | 110 of 1895 in all the Protein | 0.003118266 |
| Cellular component | GO:0044445 | cytosolic part | 38 of 377 in the list | 127 of 1895 in all the Protein | 0.003424252 |
| Cellular component | GO:0009514 | glyoxysome | 3 of 377 in the list | 3 of 1895 in all the Protein | 0.007823853 |
| Cellular component | GO:0015935 | small ribosomal subunit | 14 of 377 in the list | 37 of 1895 in all the Protein | 0.008244014 |
| Cellular component | GO:0032991 | macromolecular complex | 122 of 377 in the list | 525 of 1895 in all the Protein | 0.01488784 |
| Cellular component | GO:0043226 | organelle | 250 of 377 in the list | 1180 of 1895 in all the Protein | 0.03943755 |
| Cellular component | GO:0043229 | intracellular organelle | 250 of 377 in the list | 1180 of 1895 in all the Protein | 0.03943755 |
| Cellular component | GO:0000275 | mitochondrial proton-transporting ATP synthase complex, catalytic core F(1) | 2 of 377 in the list | 2 of 1895 in all the Protein | 0.03949481 |
| Cellular component | GO:0005732 | small nucleolar ribonucleoprotein complex | 2 of 377 in the list | 2 of 1895 in all the Protein | 0.03949481 |
| Cellular component | GO:0030684 | preribosome | 2 of 377 in the list | 2 of 1895 in all the Protein | 0.03949481 |
| Cellular component | GO:0030686 | 90S preribosome | 2 of 377 in the list | 2 of 1895 in all the Protein | 0.03949481 |
| Cellular component | GO:0015934 | large ribosomal subunit | 16 of 377 in the list | 52 of 1895 in all the Protein | 0.03954482 |
| Cellular component | GO:0022625 | cytosolic large ribosomal subunit | 14 of 377 in the list | 44 of 1895 in all the Protein | 0.04008565 |
| Cellular component | GO:0044432 | endoplasmic reticulum part | 6 of 377 in the list | 15 of 1895 in all the Protein | 0.05895529 |
| Cellular component | GO:0005789 | endoplasmic reticulum membrane | 5 of 377 in the list | 12 of 1895 in all the Protein | 0.07053212 |
| Cellular component | GO:0031224 | intrinsic to membrane | 52 of 377 in the list | 218 of 1895 in all the Protein | 0.07325282 |
| Cellular component | GO:0005773 | vacuole | 40 of 377 in the list | 164 of 1895 in all the Protein | 0.08193835 |
| Cellular component | GO:0044424 | intracellular part | 302 of 377 in the list | 1467 of 1895 in all the Protein | 0.09109536 |
| Cellular component | GO:0042175 | nuclear outer membrane-endoplasmic reticulum membrane network | 5 of 377 in the list | 13 of 1895 in all the Protein | 0.09658718 |
| Cellular component | GO:0016021 | integral to membrane | 48 of 377 in the list | 205 of 1895 in all the Protein | 0.1079901 |
| Cellular component | GO:0044429 | mitochondrial part | 16 of 377 in the list | 59 of 1895 in all the Protein | 0.1087523 |
| Cellular component | GO:0005753 | mitochondrial proton-transporting ATP synthase complex | 3 of 377 in the list | 7 of 1895 in all the Protein | 0.1458561 |
| Cellular component | GO:0005740 | mitochondrial envelope | 14 of 377 in the list | 53 of 1895 in all the Protein | 0.1511731 |
| Cellular component | GO:0005743 | mitochondrial inner membrane | 9 of 377 in the list | 32 of 1895 in all the Protein | 0.1687593 |
| Cellular component | GO:0005829 | cytosol | 56 of 377 in the list | 251 of 1895 in all the Protein | 0.1720295 |
| Cellular component | GO:0000145 | exocyst | 2 of 377 in the list | 4 of 1895 in all the Protein | 0.1790178 |
| Cellular component | GO:0031227 | intrinsic to endoplasmic reticulum membrane | 2 of 377 in the list | 4 of 1895 in all the Protein | 0.1790178 |
| Cellular component | GO:0031965 | nuclear membrane | 2 of 377 in the list | 4 of 1895 in all the Protein | 0.1790178 |
| Cellular component | GO:0044422 | organelle part | 127 of 377 in the list | 599 of 1895 in all the Protein | 0.1818707 |
| Cellular component | GO:0044446 | intracellular organelle part | 127 of 377 in the list | 599 of 1895 in all the Protein | 0.1818707 |
| Cellular component | GO:0019866 | organelle inner membrane | 10 of 377 in the list | 37 of 1895 in all the Protein | 0.1842707 |
| Cellular component | GO:0044444 | cytoplasmic part | 217 of 377 in the list | 1051 of 1895 in all the Protein | 0.1955915 |
| Cellular component | GO:0005945 | 6-phosphofructokinase complex | 3 of 377 in the list | 8 of 1895 in all the Protein | 0.2004101 |
| Cellular component | GO:0043227 | membrane-bounded organelle | 221 of 377 in the list | 1072 of 1895 in all the Protein | 0.2007255 |
| Cellular component | GO:0043231 | intracellular membrane-bounded organelle | 221 of 377 in the list | 1072 of 1895 in all the Protein | 0.2007255 |
| Cellular component | GO:0005622 | intracellular | 311 of 377 in the list | 1536 of 1895 in all the Protein | 0.2363225 |
| Cellular component | GO:0031966 | mitochondrial membrane | 12 of 377 in the list | 49 of 1895 in all the Protein | 0.2560854 |
| Cellular component | GO:0015629 | actin cytoskeleton | 3 of 377 in the list | 9 of 1895 in all the Protein | 0.2587321 |
| Cellular component | GO:0005938 | cell cortex | 2 of 377 in the list | 5 of 1895 in all the Protein | 0.2604494 |
| Cellular component | GO:0044448 | cell cortex part | 2 of 377 in the list | 5 of 1895 in all the Protein | 0.2604494 |
| Cellular component | GO:0005737 | cytoplasm | 258 of 377 in the list | 1273 of 1895 in all the Protein | 0.3026235 |
| Cellular component | GO:0019773 | proteasome core complex, alpha-subunit complex | 3 of 377 in the list | 10 of 1895 in all the Protein | 0.3188476 |
| Cellular component | GO:0005759 | mitochondrial matrix | 2 of 377 in the list | 6 of 1895 in all the Protein | 0.3420426 |
| Cellular component | GO:0030054 | cell junction | 9 of 377 in the list | 39 of 1895 in all the Protein | 0.368592 |
| Cellular component | GO:0031090 | organelle membrane | 30 of 377 in the list | 143 of 1895 in all the Protein | 0.4026715 |
| Cellular component | GO:0071944 | cell periphery | 104 of 377 in the list | 511 of 1895 in all the Protein | 0.4035721 |
| Cellular component | GO:0000151 | ubiquitin ligase complex | 3 of 377 in the list | 12 of 1895 in all the Protein | 0.4380888 |
| Cellular component | GO:0031300 | intrinsic to organelle membrane | 3 of 377 in the list | 12 of 1895 in all the Protein | 0.4380888 |
| Cellular component | GO:0046658 | anchored to plasma membrane | 3 of 377 in the list | 12 of 1895 in all the Protein | 0.4380888 |
| Cellular component | GO:0070469 | respiratory chain | 5 of 377 in the list | 22 of 1895 in all the Protein | 0.4523316 |
| Cellular component | GO:0044425 | membrane part | 67 of 377 in the list | 332 of 1895 in all the Protein | 0.4689136 |
| Cellular component | GO:0005618 | cell wall | 32 of 377 in the list | 157 of 1895 in all the Protein | 0.4709325 |
| Cellular component | GO:0030312 | external encapsulating structure | 32 of 377 in the list | 157 of 1895 in all the Protein | 0.4709325 |
| Cellular component | GO:0031461 | cullin-RING ubiquitin ligase complex | 2 of 377 in the list | 8 of 1895 in all the Protein | 0.4938863 |
| Cellular component | GO:0045261 | proton-transporting ATP synthase complex, catalytic core F(1) | 2 of 377 in the list | 8 of 1895 in all the Protein | 0.4938863 |
| Cellular component | GO:0031225 | anchored to membrane | 3 of 377 in the list | 13 of 1895 in all the Protein | 0.4948323 |
| Cellular component | GO:0005839 | proteasome core complex | 4 of 377 in the list | 18 of 1895 in all the Protein | 0.4948607 |
| Cellular component | GO:0005886 | plasma membrane | 83 of 377 in the list | 420 of 1895 in all the Protein | 0.5551847 |
| Cellular component | GO:0005654 | nucleoplasm | 2 of 377 in the list | 9 of 1895 in all the Protein | 0.5610733 |
| Cellular component | GO:0009522 | photosystem I | 2 of 377 in the list | 9 of 1895 in all the Protein | 0.5610733 |
| Cellular component | GO:0031301 | integral to organelle membrane | 2 of 377 in the list | 9 of 1895 in all the Protein | 0.5610733 |
| Cellular component | GO:0044451 | nucleoplasm part | 2 of 377 in the list | 9 of 1895 in all the Protein | 0.5610733 |
| Cellular component | GO:0042651 | thylakoid membrane | 12 of 377 in the list | 61 of 1895 in all the Protein | 0.5702766 |
| Cellular component | GO:0009535 | chloroplast thylakoid membrane | 11 of 377 in the list | 56 of 1895 in all the Protein | 0.5738725 |
| Cellular component | GO:0055035 | plastid thylakoid membrane | 11 of 377 in the list | 56 of 1895 in all the Protein | 0.5738725 |
| Cellular component | GO:0005874 | microtubule | 4 of 377 in the list | 20 of 1895 in all the Protein | 0.5848415 |
| Cellular component | GO:0005623 | cell | 338 of 377 in the list | 1702 of 1895 in all the Protein | 0.5887962 |
| Cellular component | GO:0044464 | cell part | 338 of 377 in the list | 1702 of 1895 in all the Protein | 0.5887962 |
| Cellular component | GO:0005911 | cell-cell junction | 7 of 377 in the list | 36 of 1895 in all the Protein | 0.594678 |
| Cellular component | GO:0009506 | plasmodesma | 7 of 377 in the list | 36 of 1895 in all the Protein | 0.594678 |
| Cellular component | GO:0055044 | symplast | 7 of 377 in the list | 36 of 1895 in all the Protein | 0.594678 |
| Cellular component | GO:0033178 | proton-transporting two-sector ATPase complex, catalytic domain | 3 of 377 in the list | 15 of 1895 in all the Protein | 0.5987913 |
| Cellular component | GO:0045259 | proton-transporting ATP synthase complex | 3 of 377 in the list | 15 of 1895 in all the Protein | 0.5987913 |
| Cellular component | GO:0034357 | photosynthetic membrane | 14 of 377 in the list | 73 of 1895 in all the Protein | 0.610238 |
| Cellular component | GO:0016469 | proton-transporting two-sector ATPase complex | 5 of 377 in the list | 26 of 1895 in all the Protein | 0.6127877 |
| Cellular component | GO:0005794 | Golgi apparatus | 17 of 377 in the list | 89 of 1895 in all the Protein | 0.6199335 |
| Cellular component | GO:0009505 | plant-type cell wall | 10 of 377 in the list | 53 of 1895 in all the Protein | 0.6309556 |
| Cellular component | GO:0031226 | intrinsic to plasma membrane | 3 of 377 in the list | 16 of 1895 in all the Protein | 0.6452145 |
| Cellular component | GO:0044455 | mitochondrial membrane part | 5 of 377 in the list | 27 of 1895 in all the Protein | 0.6484106 |
| Cellular component | GO:0033177 | proton-transporting two-sector ATPase complex, proton-transporting domain | 2 of 377 in the list | 11 of 1895 in all the Protein | 0.675529 |
| Cellular component | GO:0015630 | microtubule cytoskeleton | 5 of 377 in the list | 28 of 1895 in all the Protein | 0.681901 |
| Cellular component | GO:0005856 | cytoskeleton | 7 of 377 in the list | 39 of 1895 in all the Protein | 0.684145 |
| Cellular component | GO:0016020 | membrane | 172 of 377 in the list | 886 of 1895 in all the Protein | 0.708483 |
| Cellular component | GO:0005774 | vacuolar membrane | 7 of 377 in the list | 41 of 1895 in all the Protein | 0.7363002 |
| Cellular component | GO:0044437 | vacuolar part | 7 of 377 in the list | 41 of 1895 in all the Protein | 0.7363002 |
| Cellular component | GO:0044436 | thylakoid part | 14 of 377 in the list | 80 of 1895 in all the Protein | 0.7507799 |
| Cellular component | GO:0005739 | mitochondrion | 36 of 377 in the list | 197 of 1895 in all the Protein | 0.7543336 |
| Cellular component | GO:0010319 | stromule | 2 of 377 in the list | 13 of 1895 in all the Protein | 0.7645052 |
| Cellular component | GO:0044459 | plasma membrane part | 5 of 377 in the list | 31 of 1895 in all the Protein | 0.769086 |
| Cellular component | GO:0000502 | proteasome complex | 6 of 377 in the list | 37 of 1895 in all the Protein | 0.7754068 |
| Cellular component | GO:0012505 | endomembrane system | 10 of 377 in the list | 60 of 1895 in all the Protein | 0.7850362 |
| Cellular component | GO:0044430 | cytoskeletal part | 5 of 377 in the list | 32 of 1895 in all the Protein | 0.7937527 |
| Cellular component | GO:0005777 | peroxisome | 7 of 377 in the list | 44 of 1895 in all the Protein | 0.8027616 |
| Cellular component | GO:0042579 | microbody | 7 of 377 in the list | 44 of 1895 in all the Protein | 0.8027616 |
| Cellular component | GO:0009534 | chloroplast thylakoid | 12 of 377 in the list | 73 of 1895 in all the Protein | 0.8156945 |
| Cellular component | GO:0031976 | plastid thylakoid | 12 of 377 in the list | 73 of 1895 in all the Protein | 0.8156945 |
| Cellular component | GO:0031984 | organelle subcompartment | 12 of 377 in the list | 73 of 1895 in all the Protein | 0.8156945 |
| Cellular component | GO:0030964 | NADH dehydrogenase complex | 2 of 377 in the list | 15 of 1895 in all the Protein | 0.8315263 |
| Cellular component | GO:0045271 | respiratory chain complex I | 2 of 377 in the list | 15 of 1895 in all the Protein | 0.8315263 |
| Cellular component | GO:0005783 | endoplasmic reticulum | 15 of 377 in the list | 93 of 1895 in all the Protein | 0.8578041 |
| Cellular component | GO:0005635 | nuclear envelope | 2 of 377 in the list | 16 of 1895 in all the Protein | 0.8581423 |
| Cellular component | GO:0005746 | mitochondrial respiratory chain | 2 of 377 in the list | 16 of 1895 in all the Protein | 0.8581423 |
| Cellular component | GO:0009536 | plastid | 87 of 377 in the list | 486 of 1895 in all the Protein | 0.9111696 |
| Cellular component | GO:0000139 | Golgi membrane | 2 of 377 in the list | 19 of 1895 in all the Protein | 0.9165626 |
| Cellular component | GO:0009507 | chloroplast | 81 of 377 in the list | 456 of 1895 in all the Protein | 0.9165725 |
| Cellular component | GO:0009579 | thylakoid | 16 of 377 in the list | 108 of 1895 in all the Protein | 0.9354805 |
| Cellular component | GO:0031967 | organelle envelope | 29 of 377 in the list | 182 of 1895 in all the Protein | 0.9367858 |
| Cellular component | GO:0031975 | envelope | 29 of 377 in the list | 182 of 1895 in all the Protein | 0.9367858 |
| Cellular component | GO:0048046 | apoplast | 14 of 377 in the list | 97 of 1895 in all the Protein | 0.9395331 |
| Cellular component | GO:0016023 | cytoplasmic membrane-bounded vesicle | 3 of 377 in the list | 32 of 1895 in all the Protein | 0.9683613 |
| Cellular component | GO:0031410 | cytoplasmic vesicle | 3 of 377 in the list | 32 of 1895 in all the Protein | 0.9683613 |
| Cellular component | GO:0031982 | vesicle | 3 of 377 in the list | 32 of 1895 in all the Protein | 0.9683613 |
| Cellular component | GO:0031988 | membrane-bounded vesicle | 3 of 377 in the list | 32 of 1895 in all the Protein | 0.9683613 |
| Cellular component | GO:0009521 | photosystem | 2 of 377 in the list | 25 of 1895 in all the Protein | 0.9725746 |
| Cellular component | GO:0009941 | chloroplast envelope | 15 of 377 in the list | 115 of 1895 in all the Protein | 0.9821849 |
| Cellular component | GO:0043234 | protein complex | 42 of 377 in the list | 272 of 1895 in all the Protein | 0.9827567 |
| Cellular component | GO:0009526 | plastid envelope | 15 of 377 in the list | 118 of 1895 in all the Protein | 0.9871746 |
| Cellular component | GO:0044431 | Golgi apparatus part | 2 of 377 in the list | 29 of 1895 in all the Protein | 0.9872977 |
| Cellular component | GO:0005576 | extracellular region | 16 of 377 in the list | 126 of 1895 in all the Protein | 0.9894974 |
| Cellular component | GO:0030117 | membrane coat | 2 of 377 in the list | 31 of 1895 in all the Protein | 0.9914119 |
| Cellular component | GO:0048475 | coated membrane | 2 of 377 in the list | 31 of 1895 in all the Protein | 0.9914119 |
| Cellular component | GO:0044434 | chloroplast part | 28 of 377 in the list | 223 of 1895 in all the Protein | 0.999184 |
| Cellular component | GO:0044435 | plastid part | 28 of 377 in the list | 224 of 1895 in all the Protein | 0.9992703 |
| Cellular component | GO:0009570 | chloroplast stroma | 7 of 377 in the list | 110 of 1895 in all the Protein | 0.9999895 |
| Cellular component | GO:0009532 | plastid stroma | 7 of 377 in the list | 111 of 1895 in all the Protein | 0.9999912 |
| Molecular Function | GO:0003677 | DNA binding | 27 of 535 in the list | 82 of 2767 in all the Protein | 0.00215231 |
| Molecular Function | GO:0003676 | nucleic acid binding | 81 of 535 in the list | 320 of 2767 in all the Protein | 0.003132084 |
| Molecular Function | GO:0003735 | structural constituent of ribosome | 41 of 535 in the list | 146 of 2767 in all the Protein | 0.00545499 |
| Molecular Function | GO:0016410 | N-acyltransferase activity | 7 of 535 in the list | 13 of 2767 in all the Protein | 0.005645765 |
| Molecular Function | GO:0016746 | transferase activity, transferring acyl groups | 22 of 535 in the list | 68 of 2767 in all the Protein | 0.006846245 |
| Molecular Function | GO:0004128 | cytochrome-b5 reductase activity, acting on NAD(P)H | 3 of 535 in the list | 3 of 2767 in all the Protein | 0.00719558 |
| Molecular Function | GO:0016407 | acetyltransferase activity | 8 of 535 in the list | 17 of 2767 in all the Protein | 0.008682061 |
| Molecular Function | GO:0015291 | secondary active transmembrane transporter activity | 7 of 535 in the list | 14 of 2767 in all the Protein | 0.009437382 |
| Molecular Function | GO:0008080 | N-acetyltransferase activity | 6 of 535 in the list | 11 of 2767 in all the Protein | 0.009697248 |
| Molecular Function | GO:0005198 | structural molecule activity | 47 of 535 in the list | 178 of 2767 in all the Protein | 0.01065236 |
| Molecular Function | GO:0015297 | antiporter activity | 4 of 535 in the list | 6 of 2767 in all the Protein | 0.01489726 |
| Molecular Function | GO:0004185 | serine-type carboxypeptidase activity | 6 of 535 in the list | 12 of 2767 in all the Protein | 0.01627464 |
| Molecular Function | GO:0016653 | oxidoreductase activity, acting on NAD(P)H, heme protein as acceptor | 3 of 535 in the list | 4 of 2767 in all the Protein | 0.02462742 |
| Molecular Function | GO:0070008 | serine-type exopeptidase activity | 6 of 535 in the list | 13 of 2767 in all the Protein | 0.02538511 |
| Molecular Function | GO:0004180 | carboxypeptidase activity | 8 of 535 in the list | 21 of 2767 in all the Protein | 0.03533049 |
| Molecular Function | GO:0000036 | ACP phosphopantetheine attachment site binding involved in fatty acid biosynthetic process | 2 of 535 in the list | 2 of 2767 in all the Protein | 0.03732791 |
| Molecular Function | GO:0005452 | inorganic anion exchanger activity | 2 of 535 in the list | 2 of 2767 in all the Protein | 0.03732791 |
| Molecular Function | GO:0008839 | 4-hydroxy-tetrahydrodipicolinate reductase | 2 of 535 in the list | 2 of 2767 in all the Protein | 0.03732791 |
| Molecular Function | GO:0031177 | phosphopantetheine binding | 2 of 535 in the list | 2 of 2767 in all the Protein | 0.03732791 |
| Molecular Function | GO:0044620 | ACP phosphopantetheine attachment site binding | 2 of 535 in the list | 2 of 2767 in all the Protein | 0.03732791 |
| Molecular Function | GO:0050253 | retinyl-palmitate esterase activity | 2 of 535 in the list | 2 of 2767 in all the Protein | 0.03732791 |
| Molecular Function | GO:0051192 | prosthetic group binding | 2 of 535 in the list | 2 of 2767 in all the Protein | 0.03732791 |
| Molecular Function | GO:0080030 | methyl indole-3-acetate esterase activity | 2 of 535 in the list | 2 of 2767 in all the Protein | 0.03732791 |
| Molecular Function | GO:0080031 | methyl salicylate esterase activity | 2 of 535 in the list | 2 of 2767 in all the Protein | 0.03732791 |
| Molecular Function | GO:0080032 | methyl jasmonate esterase activity | 2 of 535 in the list | 2 of 2767 in all the Protein | 0.03732791 |
| Molecular Function | GO:0003723 | RNA binding | 38 of 535 in the list | 151 of 2767 in all the Protein | 0.04226005 |
| Molecular Function | GO:0004091 | carboxylesterase activity | 3 of 535 in the list | 5 of 2767 in all the Protein | 0.05277829 |
| Molecular Function | GO:0004478 | methionine adenosyltransferase activity | 3 of 535 in the list | 5 of 2767 in all the Protein | 0.05277829 |
| Molecular Function | GO:0004709 | MAP kinase kinase kinase activity | 3 of 535 in the list | 5 of 2767 in all the Protein | 0.05277829 |
| Molecular Function | GO:0008061 | chitin binding | 3 of 535 in the list | 5 of 2767 in all the Protein | 0.05277829 |
| Molecular Function | GO:0097367 | carbohydrate derivative binding | 3 of 535 in the list | 5 of 2767 in all the Protein | 0.05277829 |
| Molecular Function | GO:0016747 | transferase activity, transferring acyl groups other than amino-acyl groups | 15 of 535 in the list | 51 of 2767 in all the Protein | 0.05348293 |
| Molecular Function | GO:0022804 | active transmembrane transporter activity | 19 of 535 in the list | 69 of 2767 in all the Protein | 0.0599296 |
| Molecular Function | GO:0008443 | phosphofructokinase activity | 4 of 535 in the list | 9 of 2767 in all the Protein | 0.07678464 |
| Molecular Function | GO:0004601 | peroxidase activity | 9 of 535 in the list | 29 of 2767 in all the Protein | 0.0904484 |
| Molecular Function | GO:0016684 | oxidoreductase activity, acting on peroxide as acceptor | 9 of 535 in the list | 29 of 2767 in all the Protein | 0.0904484 |
| Molecular Function | GO:0033218 | amide binding | 3 of 535 in the list | 6 of 2767 in all the Protein | 0.09065932 |
| Molecular Function | GO:0005342 | organic acid transmembrane transporter activity | 2 of 535 in the list | 3 of 2767 in all the Protein | 0.09759258 |
| Molecular Function | GO:0008094 | DNA-dependent ATPase activity | 2 of 535 in the list | 3 of 2767 in all the Protein | 0.09759258 |
| Molecular Function | GO:0008430 | selenium binding | 2 of 535 in the list | 3 of 2767 in all the Protein | 0.09759258 |
| Molecular Function | GO:0008466 | glycogenin glucosyltransferase activity | 2 of 535 in the list | 3 of 2767 in all the Protein | 0.09759258 |
| Molecular Function | GO:0009011 | starch synthase activity | 2 of 535 in the list | 3 of 2767 in all the Protein | 0.09759258 |
| Molecular Function | GO:0015301 | anion:anion antiporter activity | 2 of 535 in the list | 3 of 2767 in all the Protein | 0.09759258 |
| Molecular Function | GO:0016413 | O-acetyltransferase activity | 2 of 535 in the list | 3 of 2767 in all the Protein | 0.09759258 |
| Molecular Function | GO:0016841 | ammonia-lyase activity | 2 of 535 in the list | 3 of 2767 in all the Protein | 0.09759258 |
| Molecular Function | GO:0018456 | aryl-alcohol dehydrogenase (NAD+) activity | 2 of 535 in the list | 3 of 2767 in all the Protein | 0.09759258 |
| Molecular Function | GO:0045289 | luciferin monooxygenase activity | 2 of 535 in the list | 3 of 2767 in all the Protein | 0.09759258 |
| Molecular Function | GO:0045548 | phenylalanine ammonia-lyase activity | 2 of 535 in the list | 3 of 2767 in all the Protein | 0.09759258 |
| Molecular Function | GO:0045551 | cinnamyl-alcohol dehydrogenase activity | 2 of 535 in the list | 3 of 2767 in all the Protein | 0.09759258 |
| Molecular Function | GO:0047077 | Photinus-luciferin 4-monooxygenase (ATP-hydrolyzing) activity | 2 of 535 in the list | 3 of 2767 in all the Protein | 0.09759258 |
| Molecular Function | GO:0072341 | modified amino acid binding | 2 of 535 in the list | 3 of 2767 in all the Protein | 0.09759258 |
| Molecular Function | GO:0008514 | organic anion transmembrane transporter activity | 4 of 535 in the list | 10 of 2767 in all the Protein | 0.1090723 |
| Molecular Function | GO:0042623 | ATPase activity, coupled | 24 of 535 in the list | 97 of 2767 in all the Protein | 0.1091738 |
| Molecular Function | GO:0008509 | anion transmembrane transporter activity | 8 of 535 in the list | 27 of 2767 in all the Protein | 0.1335146 |
| Molecular Function | GO:0004402 | histone acetyltransferase activity | 3 of 535 in the list | 7 of 2767 in all the Protein | 0.1365323 |
| Molecular Function | GO:0016887 | ATPase activity | 24 of 535 in the list | 101 of 2767 in all the Protein | 0.1539849 |
| Molecular Function | GO:0003729 | mRNA binding | 2 of 535 in the list | 4 of 2767 in all the Protein | 0.1705577 |
| Molecular Function | GO:0004725 | protein tyrosine phosphatase activity | 2 of 535 in the list | 4 of 2767 in all the Protein | 0.1705577 |
| Molecular Function | GO:0005351 | sugar:hydrogen symporter activity | 2 of 535 in the list | 4 of 2767 in all the Protein | 0.1705577 |
| Molecular Function | GO:0005402 | cation:sugar symporter activity | 2 of 535 in the list | 4 of 2767 in all the Protein | 0.1705577 |
| Molecular Function | GO:0015293 | symporter activity | 2 of 535 in the list | 4 of 2767 in all the Protein | 0.1705577 |
| Molecular Function | GO:0015294 | solute:cation symporter activity | 2 of 535 in the list | 4 of 2767 in all the Protein | 0.1705577 |
| Molecular Function | GO:0015295 | solute:hydrogen symporter activity | 2 of 535 in the list | 4 of 2767 in all the Protein | 0.1705577 |
| Molecular Function | GO:0015416 | organic phosphonate transmembrane-transporting ATPase activity | 2 of 535 in the list | 4 of 2767 in all the Protein | 0.1705577 |
| Molecular Function | GO:0015604 | organic phosphonate transmembrane transporter activity | 2 of 535 in the list | 4 of 2767 in all the Protein | 0.1705577 |
| Molecular Function | GO:0015605 | organophosphate ester transmembrane transporter activity | 2 of 535 in the list | 4 of 2767 in all the Protein | 0.1705577 |
| Molecular Function | GO:0048038 | quinone binding | 2 of 535 in the list | 4 of 2767 in all the Protein | 0.1705577 |
| Molecular Function | GO:0070402 | NADPH binding | 2 of 535 in the list | 4 of 2767 in all the Protein | 0.1705577 |
| Molecular Function | GO:0016635 | oxidoreductase activity, acting on the CH-CH group of donors, quinone or related compound as acceptor | 2 of 535 in the list | 4 of 2767 in all the Protein | 0.1705577 |
| Molecular Function | GO:0008236 | serine-type peptidase activity | 13 of 535 in the list | 51 of 2767 in all the Protein | 0.1711409 |
| Molecular Function | GO:0017171 | serine hydrolase activity | 13 of 535 in the list | 51 of 2767 in all the Protein | 0.1711409 |
| Molecular Function | GO:0016788 | hydrolase activity, acting on ester bonds | 36 of 535 in the list | 160 of 2767 in all the Protein | 0.1726516 |
| Molecular Function | GO:0008234 | cysteine-type peptidase activity | 5 of 535 in the list | 16 of 2767 in all the Protein | 0.1816469 |
| Molecular Function | GO:0008026 | ATP-dependent helicase activity | 8 of 535 in the list | 29 of 2767 in all the Protein | 0.1824801 |
| Molecular Function | GO:0070035 | purine NTP-dependent helicase activity | 8 of 535 in the list | 29 of 2767 in all the Protein | 0.1824801 |
| Molecular Function | GO:0003872 | 6-phosphofructokinase activity | 3 of 535 in the list | 8 of 2767 in all the Protein | 0.1883754 |
| Molecular Function | GO:0004715 | non-membrane spanning protein tyrosine kinase activity | 3 of 535 in the list | 8 of 2767 in all the Protein | 0.1883754 |
| Molecular Function | GO:0030060 | L-malate dehydrogenase activity | 3 of 535 in the list | 8 of 2767 in all the Protein | 0.1883754 |
| Molecular Function | GO:0051920 | peroxiredoxin activity | 3 of 535 in the list | 8 of 2767 in all the Protein | 0.1883754 |
| Molecular Function | GO:0015075 | ion transmembrane transporter activity | 20 of 535 in the list | 85 of 2767 in all the Protein | 0.1941559 |
| Molecular Function | GO:0042626 | ATPase activity, coupled to transmembrane movement of substances | 12 of 535 in the list | 48 of 2767 in all the Protein | 0.2030538 |
| Molecular Function | GO:0043492 | ATPase activity, coupled to movement of substances | 12 of 535 in the list | 48 of 2767 in all the Protein | 0.2030538 |
| Molecular Function | GO:0042625 | ATPase activity, coupled to transmembrane movement of ions | 10 of 535 in the list | 39 of 2767 in all the Protein | 0.2074606 |
| Molecular Function | GO:0046527 | glucosyltransferase activity | 10 of 535 in the list | 39 of 2767 in all the Protein | 0.2074606 |
| Molecular Function | GO:0016881 | acid-amino acid ligase activity | 8 of 535 in the list | 30 of 2767 in all the Protein | 0.2095048 |
| Molecular Function | GO:0016627 | oxidoreductase activity, acting on the CH-CH group of donors | 18 of 535 in the list | 77 of 2767 in all the Protein | 0.2187855 |
| Molecular Function | GO:0008238 | exopeptidase activity | 9 of 535 in the list | 35 of 2767 in all the Protein | 0.2219408 |
| Molecular Function | GO:0016820 | hydrolase activity, acting on acid anhydrides, catalyzing transmembrane movement of substances | 12 of 535 in the list | 49 of 2767 in all the Protein | 0.2248761 |
| Molecular Function | GO:0016209 | antioxidant activity | 10 of 535 in the list | 40 of 2767 in all the Protein | 0.2320873 |
| Molecular Function | GO:0031406 | carboxylic acid binding | 4 of 535 in the list | 13 of 2767 in all the Protein | 0.232152 |
| Molecular Function | GO:0004871 | signal transducer activity | 6 of 535 in the list | 22 of 2767 in all the Protein | 0.2403533 |
| Molecular Function | GO:0060089 | molecular transducer activity | 6 of 535 in the list | 22 of 2767 in all the Protein | 0.2403533 |
| Molecular Function | GO:0004702 | receptor signaling protein serine/threonine kinase activity | 3 of 535 in the list | 9 of 2767 in all the Protein | 0.2441707 |
| Molecular Function | GO:0005057 | receptor signaling protein activity | 3 of 535 in the list | 9 of 2767 in all the Protein | 0.2441707 |
| Molecular Function | GO:0016597 | amino acid binding | 3 of 535 in the list | 9 of 2767 in all the Protein | 0.2441707 |
| Molecular Function | GO:0042973 | glucan endo-1,3-beta-D-glucosidase activity | 3 of 535 in the list | 9 of 2767 in all the Protein | 0.2441707 |
| Molecular Function | GO:0003690 | double-stranded DNA binding | 2 of 535 in the list | 5 of 2767 in all the Protein | 0.2490774 |
| Molecular Function | GO:0004467 | long-chain fatty acid-CoA ligase activity | 2 of 535 in the list | 5 of 2767 in all the Protein | 0.2490774 |
| Molecular Function | GO:0004630 | phospholipase D activity | 2 of 535 in the list | 5 of 2767 in all the Protein | 0.2490774 |
| Molecular Function | GO:0008170 | N-methyltransferase activity | 2 of 535 in the list | 5 of 2767 in all the Protein | 0.2490774 |
| Molecular Function | GO:0015645 | fatty acid ligase activity | 2 of 535 in the list | 5 of 2767 in all the Protein | 0.2490774 |
| Molecular Function | GO:0019238 | cyclohydrolase activity | 2 of 535 in the list | 5 of 2767 in all the Protein | 0.2490774 |
| Molecular Function | GO:0043022 | ribosome binding | 2 of 535 in the list | 5 of 2767 in all the Protein | 0.2490774 |
| Molecular Function | GO:0070290 | NAPE-specific phospholipase D activity | 2 of 535 in the list | 5 of 2767 in all the Protein | 0.2490774 |
| Molecular Function | GO:0016877 | ligase activity, forming carbon-sulfur bonds | 5 of 535 in the list | 18 of 2767 in all the Protein | 0.2584451 |
| Molecular Function | GO:0022857 | transmembrane transporter activity | 27 of 535 in the list | 123 of 2767 in all the Protein | 0.2586051 |
| Molecular Function | GO:0016758 | transferase activity, transferring hexosyl groups | 16 of 535 in the list | 70 of 2767 in all the Protein | 0.2672272 |
| Molecular Function | GO:0008289 | lipid binding | 6 of 535 in the list | 23 of 2767 in all the Protein | 0.2761054 |
| Molecular Function | GO:0031072 | heat shock protein binding | 5 of 535 in the list | 19 of 2767 in all the Protein | 0.299376 |
| Molecular Function | GO:0046906 | tetrapyrrole binding | 13 of 535 in the list | 57 of 2767 in all the Protein | 0.2995384 |
| Molecular Function | GO:0004576 | oligosaccharyl transferase activity | 3 of 535 in the list | 10 of 2767 in all the Protein | 0.3020701 |
| Molecular Function | GO:0016645 | oxidoreductase activity, acting on the CH-NH group of donors | 3 of 535 in the list | 10 of 2767 in all the Protein | 0.3020701 |
| Molecular Function | GO:0019842 | vitamin binding | 3 of 535 in the list | 10 of 2767 in all the Protein | 0.3020701 |
| Molecular Function | GO:1901363 | heterocyclic compound binding | 209 of 535 in the list | 1053 of 2767 in all the Protein | 0.3128311 |
| Molecular Function | GO:0097159 | organic cyclic compound binding | 209 of 535 in the list | 1054 of 2767 in all the Protein | 0.3196687 |
| Molecular Function | GO:0015662 | ATPase activity, coupled to transmembrane movement of ions, phosphorylative mechanism | 4 of 535 in the list | 15 of 2767 in all the Protein | 0.3268099 |
| Molecular Function | GO:0044769 | ATPase activity, coupled to transmembrane movement of ions, rotational mechanism | 4 of 535 in the list | 15 of 2767 in all the Protein | 0.3268099 |
| Molecular Function | GO:0046961 | proton-transporting ATPase activity, rotational mechanism | 4 of 535 in the list | 15 of 2767 in all the Protein | 0.3268099 |
| Molecular Function | GO:0015144 | carbohydrate transmembrane transporter activity | 2 of 535 in the list | 6 of 2767 in all the Protein | 0.3282864 |
| Molecular Function | GO:0016671 | oxidoreductase activity, acting on a sulfur group of donors, disulfide as acceptor | 2 of 535 in the list | 6 of 2767 in all the Protein | 0.3282864 |
| Molecular Function | GO:0016703 | oxidoreductase activity, acting on single donors with incorporation of molecular oxygen, incorporation of one atom of oxygen (internal monooxygenases or internal mixed function oxidases) | 2 of 535 in the list | 6 of 2767 in all the Protein | 0.3282864 |
| Molecular Function | GO:0016730 | oxidoreductase activity, acting on iron-sulfur proteins as donors | 2 of 535 in the list | 6 of 2767 in all the Protein | 0.3282864 |
| Molecular Function | GO:0016731 | oxidoreductase activity, acting on iron-sulfur proteins as donors, NAD or NADP as acceptor | 2 of 535 in the list | 6 of 2767 in all the Protein | 0.3282864 |
| Molecular Function | GO:0043566 | structure-specific DNA binding | 2 of 535 in the list | 6 of 2767 in all the Protein | 0.3282864 |
| Molecular Function | GO:0051119 | sugar transmembrane transporter activity | 2 of 535 in the list | 6 of 2767 in all the Protein | 0.3282864 |
| Molecular Function | GO:1901476 | carbohydrate transporter activity | 2 of 535 in the list | 6 of 2767 in all the Protein | 0.3282864 |
| Molecular Function | GO:0022891 | substrate-specific transmembrane transporter activity | 22 of 535 in the list | 103 of 2767 in all the Protein | 0.3361816 |
| Molecular Function | GO:0019200 | carbohydrate kinase activity | 5 of 535 in the list | 20 of 2767 in all the Protein | 0.3412225 |
| Molecular Function | GO:0004386 | helicase activity | 8 of 535 in the list | 35 of 2767 in all the Protein | 0.3616922 |
| Molecular Function | GO:0020037 | heme binding | 11 of 535 in the list | 50 of 2767 in all the Protein | 0.3695864 |
| Molecular Function | GO:0005215 | transporter activity | 38 of 535 in the list | 186 of 2767 in all the Protein | 0.3779595 |
| Molecular Function | GO:0035251 | UDP-glucosyltransferase activity | 8 of 535 in the list | 36 of 2767 in all the Protein | 0.393919 |
| Molecular Function | GO:0015399 | primary active transmembrane transporter activity | 12 of 535 in the list | 56 of 2767 in all the Protein | 0.3968644 |
| Molecular Function | GO:0015405 | P-P-bond-hydrolysis-driven transmembrane transporter activity | 12 of 535 in the list | 56 of 2767 in all the Protein | 0.3968644 |
| Molecular Function | GO:0004221 | ubiquitin thiolesterase activity | 2 of 535 in the list | 7 of 2767 in all the Protein | 0.404988 |
| Molecular Function | GO:0015238 | drug transmembrane transporter activity | 2 of 535 in the list | 7 of 2767 in all the Protein | 0.404988 |
| Molecular Function | GO:0016168 | chlorophyll binding | 2 of 535 in the list | 7 of 2767 in all the Protein | 0.404988 |
| Molecular Function | GO:0043021 | ribonucleoprotein complex binding | 2 of 535 in the list | 7 of 2767 in all the Protein | 0.404988 |
| Molecular Function | GO:0090484 | drug transporter activity | 2 of 535 in the list | 7 of 2767 in all the Protein | 0.404988 |
| Molecular Function | GO:0004713 | protein tyrosine kinase activity | 3 of 535 in the list | 12 of 2767 in all the Protein | 0.4180866 |
| Molecular Function | GO:0016706 | oxidoreductase activity, acting on paired donors, with incorporation or reduction of molecular oxygen, 2-oxoglutarate as one donor, and incorporation of one atom each of oxygen into both donors | 3 of 535 in the list | 12 of 2767 in all the Protein | 0.4180866 |
| Molecular Function | GO:0016628 | oxidoreductase activity, acting on the CH-CH group of donors, NAD or NADP as acceptor | 13 of 535 in the list | 62 of 2767 in all the Protein | 0.4217276 |
| Molecular Function | GO:0003779 | actin binding | 4 of 535 in the list | 17 of 2767 in all the Protein | 0.4232565 |
| Molecular Function | GO:0015078 | hydrogenion transmembrane transporter activity | 7 of 535 in the list | 32 of 2767 in all the Protein | 0.4265323 |
| Molecular Function | GO:0016757 | transferase activity, transferring glycosyl groups | 21 of 535 in the list | 103 of 2767 in all the Protein | 0.4316301 |
| Molecular Function | GO:0052689 | carboxylic ester hydrolase activity | 6 of 535 in the list | 28 of 2767 in all the Protein | 0.463937 |
| Molecular Function | GO:0008757 | S-adenosylmethionine-dependent methyltransferase activity | 5 of 535 in the list | 23 of 2767 in all the Protein | 0.4670268 |
| Molecular Function | GO:0015035 | protein disulfide oxidoreductase activity | 5 of 535 in the list | 23 of 2767 in all the Protein | 0.4670268 |
| Molecular Function | GO:0016405 | CoA-ligase activity | 3 of 535 in the list | 13 of 2767 in all the Protein | 0.473867 |
| Molecular Function | GO:0016615 | malate dehydrogenase activity | 3 of 535 in the list | 13 of 2767 in all the Protein | 0.473867 |
| Molecular Function | GO:0016878 | acid-thiol ligase activity | 3 of 535 in the list | 13 of 2767 in all the Protein | 0.473867 |
| Molecular Function | GO:0004022 | alcohol dehydrogenase (NAD) activity | 2 of 535 in the list | 8 of 2767 in all the Protein | 0.4771923 |
| Molecular Function | GO:0004312 | fatty acid synthase activity | 2 of 535 in the list | 8 of 2767 in all the Protein | 0.4771923 |
| Molecular Function | GO:0016634 | oxidoreductase activity, acting on the CH-CH group of donors, oxygen as acceptor | 2 of 535 in the list | 8 of 2767 in all the Protein | 0.4771923 |
| Molecular Function | GO:0016646 | oxidoreductase activity, acting on the CH-NH group of donors, NAD or NADP as acceptor | 2 of 535 in the list | 8 of 2767 in all the Protein | 0.4771923 |
| Molecular Function | GO:0016814 | hydrolase activity, acting on carbon-nitrogen (but not peptide) bonds, in cyclic amidines | 2 of 535 in the list | 8 of 2767 in all the Protein | 0.4771923 |
| Molecular Function | GO:0016832 | aldehyde-lyase activity | 2 of 535 in the list | 8 of 2767 in all the Protein | 0.4771923 |
| Molecular Function | GO:0016840 | carbon-nitrogen lyase activity | 2 of 535 in the list | 8 of 2767 in all the Protein | 0.4771923 |
| Molecular Function | GO:0016773 | phosphotransferase activity, alcohol group as acceptor | 32 of 535 in the list | 162 of 2767 in all the Protein | 0.4781168 |
| Molecular Function | GO:0042578 | phosphoric ester hydrolase activity | 15 of 535 in the list | 75 of 2767 in all the Protein | 0.4886054 |
| Molecular Function | GO:0016667 | oxidoreductase activity, acting on a sulfur group of donors | 8 of 535 in the list | 39 of 2767 in all the Protein | 0.4901189 |
| Molecular Function | GO:0015077 | monovalent inorganic cation transmembrane transporter activity | 7 of 535 in the list | 34 of 2767 in all the Protein | 0.4950914 |
| Molecular Function | GO:0016741 | transferase activity, transferring one-carbon groups | 13 of 535 in the list | 65 of 2767 in all the Protein | 0.4960857 |
| Molecular Function | GO:0004721 | phosphoprotein phosphatase activity | 6 of 535 in the list | 29 of 2767 in all the Protein | 0.5008024 |
| Molecular Function | GO:0019829 | cation-transporting ATPase activity | 6 of 535 in the list | 29 of 2767 in all the Protein | 0.5008024 |
| Molecular Function | GO:0050661 | NADP binding | 5 of 535 in the list | 24 of 2767 in all the Protein | 0.507565 |
| Molecular Function | GO:0016879 | ligase activity, forming carbon-nitrogen bonds | 10 of 535 in the list | 50 of 2767 in all the Protein | 0.509713 |
| Molecular Function | GO:0016701 | oxidoreductase activity, acting on single donors with incorporation of molecular oxygen | 4 of 535 in the list | 19 of 2767 in all the Protein | 0.5159373 |
| Molecular Function | GO:0008194 | UDP-glycosyltransferase activity | 8 of 535 in the list | 40 of 2767 in all the Protein | 0.5213702 |
| Molecular Function | GO:0008168 | methyltransferase activity | 12 of 535 in the list | 61 of 2767 in all the Protein | 0.5255696 |
| Molecular Function | GO:0046933 | proton-transporting ATP synthase activity, rotational mechanism | 3 of 535 in the list | 14 of 2767 in all the Protein | 0.5270548 |
| Molecular Function | GO:0016491 | oxidoreductase activity | 95 of 535 in the list | 492 of 2767 in all the Protein | 0.5282318 |
| Molecular Function | GO:0008324 | cation transmembrane transporter activity | 11 of 535 in the list | 56 of 2767 in all the Protein | 0.5311396 |
| Molecular Function | GO:0016791 | phosphatase activity | 11 of 535 in the list | 56 of 2767 in all the Protein | 0.5311396 |
| Molecular Function | GO:0000049 | tRNA binding | 2 of 535 in the list | 9 of 2767 in all the Protein | 0.5437699 |
| Molecular Function | GO:0004579 | dolichyl-diphosphooligosaccharide-protein glycotransferase activity | 2 of 535 in the list | 9 of 2767 in all the Protein | 0.5437699 |
| Molecular Function | GO:0043225 | anion transmembrane-transporting ATPase activity | 2 of 535 in the list | 9 of 2767 in all the Protein | 0.5437699 |
| Molecular Function | GO:0070546 | L-phenylalanine aminotransferase activity | 2 of 535 in the list | 9 of 2767 in all the Protein | 0.5437699 |
| Molecular Function | GO:0080130 | L-phenylalanine:2-oxoglutarate aminotransferase activity | 2 of 535 in the list | 9 of 2767 in all the Protein | 0.5437699 |
| Molecular Function | GO:0022890 | inorganic cation transmembrane transporter activity | 9 of 535 in the list | 46 of 2767 in all the Protein | 0.5441505 |
| Molecular Function | GO:0016740 | transferase activity | 108 of 535 in the list | 562 of 2767 in all the Protein | 0.5524954 |
| Molecular Function | GO:0008092 | cytoskeletal protein binding | 4 of 535 in the list | 20 of 2767 in all the Protein | 0.559616 |
| Molecular Function | GO:0008422 | beta-glucosidase activity | 4 of 535 in the list | 20 of 2767 in all the Protein | 0.559616 |
| Molecular Function | GO:0004674 | protein serine/threonine kinase activity | 14 of 535 in the list | 73 of 2767 in all the Protein | 0.5619149 |
| Molecular Function | GO:0070011 | peptidase activity, acting on L-amino acid peptides | 26 of 535 in the list | 136 of 2767 in all the Protein | 0.5623715 |
| Molecular Function | GO:0022892 | substrate-specific transporter activity | 25 of 535 in the list | 131 of 2767 in all the Protein | 0.5664402 |
| Molecular Function | GO:0004672 | protein kinase activity | 20 of 535 in the list | 105 of 2767 in all the Protein | 0.5709233 |
| Molecular Function | GO:0003924 | GTPase activity | 9 of 535 in the list | 47 of 2767 in all the Protein | 0.5724221 |
| Molecular Function | GO:0009055 | electron carrier activity | 14 of 535 in the list | 74 of 2767 in all the Protein | 0.584381 |
| Molecular Function | GO:0015036 | disulfide oxidoreductase activity | 5 of 535 in the list | 26 of 2767 in all the Protein | 0.5845028 |
| Molecular Function | GO:0017111 | nucleoside-triphosphatase activity | 34 of 535 in the list | 180 of 2767 in all the Protein | 0.5939998 |
| Molecular Function | GO:0016462 | pyrophosphatase activity | 36 of 535 in the list | 191 of 2767 in all the Protein | 0.6009372 |
| Molecular Function | GO:0003746 | translation elongation factor activity | 4 of 535 in the list | 21 of 2767 in all the Protein | 0.6010696 |
| Molecular Function | GO:0016620 | oxidoreductase activity, acting on the aldehyde or oxo group of donors, NAD or NADP as acceptor | 4 of 535 in the list | 21 of 2767 in all the Protein | 0.6010696 |
| Molecular Function | GO:0016765 | transferase activity, transferring alkyl or aryl (other than methyl) groups | 4 of 535 in the list | 21 of 2767 in all the Protein | 0.6010696 |
| Molecular Function | GO:0015926 | glucosidase activity | 5 of 535 in the list | 27 of 2767 in all the Protein | 0.6204339 |
| Molecular Function | GO:0016903 | oxidoreductase activity, acting on the aldehyde or oxo group of donors | 5 of 535 in the list | 27 of 2767 in all the Protein | 0.6204339 |
| Molecular Function | GO:0004620 | phospholipase activity | 3 of 535 in the list | 16 of 2767 in all the Protein | 0.6237127 |
| Molecular Function | GO:0016790 | thiolester hydrolase activity | 3 of 535 in the list | 16 of 2767 in all the Protein | 0.6237127 |
| Molecular Function | GO:0016874 | ligase activity | 21 of 535 in the list | 114 of 2767 in all the Protein | 0.6380907 |
| Molecular Function | GO:0016818 | hydrolase activity, acting on acid anhydrides, in phosphorus-containing anhydrides | 36 of 535 in the list | 194 of 2767 in all the Protein | 0.6422382 |
| Molecular Function | GO:0004540 | ribonuclease activity | 2 of 535 in the list | 11 of 2767 in all the Protein | 0.6583539 |
| Molecular Function | GO:0008374 | O-acyltransferase activity | 2 of 535 in the list | 11 of 2767 in all the Protein | 0.6583539 |
| Molecular Function | GO:0019843 | rRNA binding | 2 of 535 in the list | 11 of 2767 in all the Protein | 0.6583539 |
| Molecular Function | GO:0004298 | threonine-type endopeptidase activity | 3 of 535 in the list | 17 of 2767 in all the Protein | 0.6666675 |
| Molecular Function | GO:0016298 | lipase activity | 3 of 535 in the list | 17 of 2767 in all the Protein | 0.6666675 |
| Molecular Function | GO:0016866 | intramolecular transferase activity | 3 of 535 in the list | 17 of 2767 in all the Protein | 0.6666675 |
| Molecular Function | GO:0070003 | threonine-type peptidase activity | 3 of 535 in the list | 17 of 2767 in all the Protein | 0.6666675 |
| Molecular Function | GO:0016817 | hydrolase activity, acting on acid anhydrides | 36 of 535 in the list | 198 of 2767 in all the Protein | 0.6941267 |
| Molecular Function | GO:0008233 | peptidase activity | 28 of 535 in the list | 156 of 2767 in all the Protein | 0.7061744 |
| Molecular Function | GO:0004842 | ubiquitin-protein ligase activity | 2 of 535 in the list | 12 of 2767 in all the Protein | 0.7064074 |
| Molecular Function | GO:0019203 | carbohydrate phosphatase activity | 2 of 535 in the list | 12 of 2767 in all the Protein | 0.7064074 |
| Molecular Function | GO:0019787 | small conjugating protein ligase activity | 2 of 535 in the list | 12 of 2767 in all the Protein | 0.7064074 |
| Molecular Function | GO:0042803 | protein homodimerization activity | 2 of 535 in the list | 12 of 2767 in all the Protein | 0.7064074 |
| Molecular Function | GO:0016301 | kinase activity | 35 of 535 in the list | 194 of 2767 in all the Protein | 0.7108725 |
| Molecular Function | GO:0032440 | 2-alkenal reductase [NAD(P)] activity | 8 of 535 in the list | 47 of 2767 in all the Protein | 0.7142652 |
| Molecular Function | GO:0004252 | serine-type endopeptidase activity | 5 of 535 in the list | 30 of 2767 in all the Protein | 0.7164107 |
| Molecular Function | GO:0016616 | oxidoreductase activity, acting on the CH-OH group of donors, NAD or NADP as acceptor | 21 of 535 in the list | 119 of 2767 in all the Protein | 0.7191977 |
| Molecular Function | GO:0016651 | oxidoreductase activity, acting on NAD(P)H | 6 of 535 in the list | 36 of 2767 in all the Protein | 0.7231804 |
| Molecular Function | GO:0005509 | calcium ion binding | 10 of 535 in the list | 59 of 2767 in all the Protein | 0.730784 |
| Molecular Function | GO:0004518 | nuclease activity | 3 of 535 in the list | 19 of 2767 in all the Protein | 0.741556 |
| Molecular Function | GO:0008081 | phosphoric diester hydrolase activity | 3 of 535 in the list | 19 of 2767 in all the Protein | 0.741556 |
| Molecular Function | GO:0005543 | phospholipid binding | 2 of 535 in the list | 13 of 2767 in all the Protein | 0.7486875 |
| Molecular Function | GO:0016702 | oxidoreductase activity, acting on single donors with incorporation of molecular oxygen, incorporation of two atoms of oxygen | 2 of 535 in the list | 13 of 2767 in all the Protein | 0.7486875 |
| Molecular Function | GO:0015925 | galactosidase activity | 2 of 535 in the list | 13 of 2767 in all the Protein | 0.7486875 |
| Molecular Function | GO:0016763 | transferase activity, transferring pentosyl groups | 2 of 535 in the list | 13 of 2767 in all the Protein | 0.7486875 |
| Molecular Function | GO:0016614 | oxidoreductase activity, acting on CH-OH group of donors | 22 of 535 in the list | 128 of 2767 in all the Protein | 0.7687813 |
| Molecular Function | GO:0050660 | flavin adenine dinucleotide binding | 5 of 535 in the list | 33 of 2767 in all the Protein | 0.7938282 |
| Molecular Function | GO:0051287 | NAD binding | 6 of 535 in the list | 39 of 2767 in all the Protein | 0.7940237 |
| Molecular Function | GO:0005506 | iron ion binding | 8 of 535 in the list | 51 of 2767 in all the Protein | 0.7980545 |
| Molecular Function | GO:0008135 | translation factor activity, nucleic acid binding | 8 of 535 in the list | 51 of 2767 in all the Protein | 0.7980545 |
| Molecular Function | GO:0016705 | oxidoreductase activity, acting on paired donors, with incorporation or reduction of molecular oxygen | 8 of 535 in the list | 52 of 2767 in all the Protein | 0.8158301 |
| Molecular Function | GO:0051213 | dioxygenase activity | 3 of 535 in the list | 22 of 2767 in all the Protein | 0.8280625 |
| Molecular Function | GO:0030246 | carbohydrate binding | 5 of 535 in the list | 35 of 2767 in all the Protein | 0.8355436 |
| Molecular Function | GO:0005507 | copper ion binding | 8 of 535 in the list | 54 of 2767 in all the Protein | 0.847753 |
| Molecular Function | GO:0046983 | protein dimerization activity | 3 of 535 in the list | 23 of 2767 in all the Protein | 0.850795 |
| Molecular Function | GO:0004683 | calmodulin-dependent protein kinase activity | 2 of 535 in the list | 17 of 2767 in all the Protein | 0.8692513 |
| Molecular Function | GO:0050897 | cobalt ion binding | 2 of 535 in the list | 17 of 2767 in all the Protein | 0.8692513 |
| Molecular Function | GO:0003755 | peptidyl-prolyl cis-trans isomerase activity | 3 of 535 in the list | 24 of 2767 in all the Protein | 0.8708655 |
| Molecular Function | GO:0008270 | zinc ion binding | 24 of 535 in the list | 149 of 2767 in all the Protein | 0.8726246 |
| Molecular Function | GO:0004175 | endopeptidase activity | 12 of 535 in the list | 80 of 2767 in all the Protein | 0.8750639 |
| Molecular Function | GO:0003743 | translation initiation factor activity | 4 of 535 in the list | 31 of 2767 in all the Protein | 0.877166 |
| Molecular Function | GO:0016859 | cis-trans isomerase activity | 3 of 535 in the list | 25 of 2767 in all the Protein | 0.888514 |
| Molecular Function | GO:0019205 | nucleobase-containing compound kinase activity | 2 of 535 in the list | 18 of 2767 in all the Protein | 0.8896662 |
| Molecular Function | GO:0005488 | binding | 296 of 535 in the list | 1595 of 2767 in all the Protein | 0.895313 |
| Molecular Function | GO:0016853 | isomerase activity | 14 of 535 in the list | 94 of 2767 in all the Protein | 0.8960091 |
| Molecular Function | GO:0008483 | transaminase activity | 3 of 535 in the list | 26 of 2767 in all the Protein | 0.9039753 |
| Molecular Function | GO:0016769 | transferase activity, transferring nitrogenous groups | 3 of 535 in the list | 26 of 2767 in all the Protein | 0.9039753 |
| Molecular Function | GO:0016787 | hydrolase activity | 125 of 535 in the list | 706 of 2767 in all the Protein | 0.9082562 |
| Molecular Function | GO:0016772 | transferase activity, transferring phosphorus-containing groups | 39 of 535 in the list | 240 of 2767 in all the Protein | 0.9139264 |
| Molecular Function | GO:0004497 | monooxygenase activity | 4 of 535 in the list | 34 of 2767 in all the Protein | 0.9181308 |
| Molecular Function | GO:0004553 | hydrolase activity, hydrolyzing O-glycosyl compounds | 15 of 535 in the list | 103 of 2767 in all the Protein | 0.9196939 |
| Molecular Function | GO:0005525 | GTP binding | 13 of 535 in the list | 91 of 2767 in all the Protein | 0.9197458 |
| Molecular Function | GO:0019001 | guanyl nucleotide binding | 13 of 535 in the list | 91 of 2767 in all the Protein | 0.9197458 |
| Molecular Function | GO:0032561 | guanyl ribonucleotide binding | 13 of 535 in the list | 91 of 2767 in all the Protein | 0.9197458 |
| Molecular Function | GO:0050662 | coenzyme binding | 20 of 535 in the list | 133 of 2767 in all the Protein | 0.9225208 |
| Molecular Function | GO:0051082 | unfolded protein binding | 6 of 535 in the list | 48 of 2767 in all the Protein | 0.9250409 |
| Molecular Function | GO:0016779 | nucleotidyltransferase activity | 4 of 535 in the list | 35 of 2767 in all the Protein | 0.9287872 |
| Molecular Function | GO:0016830 | carbon-carbon lyase activity | 4 of 535 in the list | 35 of 2767 in all the Protein | 0.9287872 |
| Molecular Function | GO:0042802 | identical protein binding | 3 of 535 in the list | 28 of 2767 in all the Protein | 0.9292221 |
| Molecular Function | GO:0051536 | iron-sulfur cluster binding | 3 of 535 in the list | 28 of 2767 in all the Protein | 0.9292221 |
| Molecular Function | GO:0051540 | metal cluster binding | 3 of 535 in the list | 28 of 2767 in all the Protein | 0.9292221 |
| Molecular Function | GO:0008565 | protein transporter activity | 3 of 535 in the list | 29 of 2767 in all the Protein | 0.9394165 |
| Molecular Function | GO:0016798 | hydrolase activity, acting on glycosyl bonds | 15 of 535 in the list | 109 of 2767 in all the Protein | 0.9529227 |
| Molecular Function | GO:0046914 | transition metal ion binding | 42 of 535 in the list | 267 of 2767 in all the Protein | 0.9532038 |
| Molecular Function | GO:0005524 | ATP binding | 61 of 535 in the list | 375 of 2767 in all the Protein | 0.9563641 |
| Molecular Function | GO:0030554 | adenyl nucleotide binding | 61 of 535 in the list | 376 of 2767 in all the Protein | 0.958736 |
| Molecular Function | GO:0032559 | adenyl ribonucleotide binding | 61 of 535 in the list | 376 of 2767 in all the Protein | 0.958736 |
| Molecular Function | GO:0036094 | small molecule binding | 135 of 535 in the list | 780 of 2767 in all the Protein | 0.9604454 |
| Molecular Function | GO:0016831 | carboxy-lyase activity | 2 of 535 in the list | 24 of 2767 in all the Protein | 0.9616908 |
| Molecular Function | GO:0043169 | cation binding | 89 of 535 in the list | 532 of 2767 in all the Protein | 0.9617836 |
| Molecular Function | GO:0016810 | hydrolase activity, acting on carbon-nitrogen (but not peptide) bonds | 3 of 535 in the list | 32 of 2767 in all the Protein | 0.9624107 |
| Molecular Function | GO:0046872 | metal ion binding | 80 of 535 in the list | 487 of 2767 in all the Protein | 0.9696488 |
| Molecular Function | GO:0016836 | hydro-lyase activity | 3 of 535 in the list | 34 of 2767 in all the Protein | 0.972875 |
| Molecular Function | GO:0000166 | nucleotide binding | 131 of 535 in the list | 767 of 2767 in all the Protein | 0.9730953 |
| Molecular Function | GO:1901265 | nucleoside phosphate binding | 131 of 535 in the list | 767 of 2767 in all the Protein | 0.9730953 |
| Molecular Function | GO:0005515 | protein binding | 25 of 535 in the list | 178 of 2767 in all the Protein | 0.9774338 |
| Molecular Function | GO:0048037 | cofactor binding | 27 of 535 in the list | 190 of 2767 in all the Protein | 0.9774601 |
| Molecular Function | GO:0001882 | nucleoside binding | 75 of 535 in the list | 468 of 2767 in all the Protein | 0.9814195 |
| Molecular Function | GO:0032549 | ribonucleoside binding | 75 of 535 in the list | 468 of 2767 in all the Protein | 0.9814195 |
| Molecular Function | GO:0035639 | purine ribonucleoside triphosphate binding | 74 of 535 in the list | 465 of 2767 in all the Protein | 0.9840636 |
| Molecular Function | GO:0001883 | purine nucleoside binding | 74 of 535 in the list | 466 of 2767 in all the Protein | 0.9849958 |
| Molecular Function | GO:0017076 | purine nucleotide binding | 74 of 535 in the list | 466 of 2767 in all the Protein | 0.9849958 |
| Molecular Function | GO:0032550 | purine ribonucleoside binding | 74 of 535 in the list | 466 of 2767 in all the Protein | 0.9849958 |
| Molecular Function | GO:0032555 | purine ribonucleotide binding | 74 of 535 in the list | 466 of 2767 in all the Protein | 0.9849958 |
| Molecular Function | GO:0000287 | magnesium ion binding | 5 of 535 in the list | 54 of 2767 in all the Protein | 0.9871178 |
| Molecular Function | GO:0032553 | ribonucleotide binding | 75 of 535 in the list | 479 of 2767 in all the Protein | 0.9905085 |
| Molecular Function | GO:0004812 | aminoacyl-tRNA ligase activity | 2 of 535 in the list | 32 of 2767 in all the Protein | 0.9913202 |
| Molecular Function | GO:0016875 | ligase activity, forming carbon-oxygen bonds | 2 of 535 in the list | 32 of 2767 in all the Protein | 0.9913202 |
| Molecular Function | GO:0016876 | ligase activity, forming aminoacyl-tRNA and related compounds | 2 of 535 in the list | 32 of 2767 in all the Protein | 0.9913202 |
| Molecular Function | GO:0016835 | carbon-oxygen lyase activity | 3 of 535 in the list | 44 of 2767 in all the Protein | 0.9950856 |
| Molecular Function | GO:0016829 | lyase activity | 11 of 535 in the list | 106 of 2767 in all the Protein | 0.996267 |
| Molecular Function | GO:0003824 | catalytic activity | 359 of 535 in the list | 1987 of 2767 in all the Protein | 0.9967377 |
| Molecular Function | GO:0043168 | anion binding | 90 of 535 in the list | 586 of 2767 in all the Protein | 0.9978718 |
| Molecular Function | GO:0030234 | enzyme regulator activity | 2 of 535 in the list | 40 of 2767 in all the Protein | 0.9981401 |
| Molecular Function | GO:0030170 | pyridoxal phosphate binding | 3 of 535 in the list | 50 of 2767 in all the Protein | 0.9983198 |
| Molecular Function | GO:0043167 | ion binding | 162 of 535 in the list | 1017 of 2767 in all the Protein | 0.9998032 |
| Biological process | GO:0006334 | nucleosome assembly | 19 of 486 in the list | 38 of 2520 in all the Protein | 1.72E-05 |
| Biological process | GO:0030036 | actin cytoskeleton organization | 3 of 486 in the list | 12 of 2520 in all the Protein | 0.4163313 |
| Biological process | GO:0007010 | cytoskeleton organization | 5 of 486 in the list | 26 of 2520 in all the Protein | 0.5820774 |
| Biological process | GO:0034728 | nucleosome organization | 19 of 486 in the list | 38 of 2520 in all the Protein | 1.72E-05 |
| Biological process | GO:0065004 | protein-DNA complex assembly | 19 of 486 in the list | 38 of 2520 in all the Protein | 1.72E-05 |
| Biological process | GO:0071824 | protein-DNA complex subunit organization | 19 of 486 in the list | 38 of 2520 in all the Protein | 1.72E-05 |
| Biological process | GO:0031497 | chromatin assembly | 19 of 486 in the list | 39 of 2520 in all the Protein | 2.76E-05 |
| Biological process | GO:0071103 | DNA conformation change | 21 of 486 in the list | 46 of 2520 in all the Protein | 3.62E-05 |
| Biological process | GO:0006323 | DNA packaging | 19 of 486 in the list | 40 of 2520 in all the Protein | 4.33E-05 |
| Biological process | GO:0006333 | chromatin assembly or disassembly | 19 of 486 in the list | 40 of 2520 in all the Protein | 4.33E-05 |
| Biological process | GO:0034622 | cellular macromolecular complex assembly | 27 of 486 in the list | 71 of 2520 in all the Protein | 0.000148839 |
| Biological process | GO:0065003 | macromolecular complex assembly | 28 of 486 in the list | 75 of 2520 in all the Protein | 0.000163127 |
| Biological process | GO:0006325 | chromatin organization | 23 of 486 in the list | 58 of 2520 in all the Protein | 0.000223738 |
| Biological process | GO:0006259 | DNA metabolic process | 25 of 486 in the list | 66 of 2520 in all the Protein | 0.000281639 |
| Biological process | GO:0051276 | chromosome organization | 23 of 486 in the list | 61 of 2520 in all the Protein | 0.000532597 |
| Biological process | GO:0043933 | macromolecular complex subunit organization | 29 of 486 in the list | 85 of 2520 in all the Protein | 0.000735592 |
| Biological process | GO:0022607 | cellular component assembly | 28 of 486 in the list | 87 of 2520 in all the Protein | 0.002486031 |
| Biological process | GO:0006996 | organelle organization | 41 of 486 in the list | 142 of 2520 in all the Protein | 0.002933739 |
| Biological process | GO:0006643 | membrane lipid metabolic process | 7 of 486 in the list | 14 of 2520 in all the Protein | 0.009285248 |
| Biological process | GO:0007030 | Golgi organization | 6 of 486 in the list | 11 of 2520 in all the Protein | 0.009559143 |
| Biological process | GO:0006665 | sphingolipid metabolic process | 4 of 486 in the list | 6 of 2520 in all the Protein | 0.01474901 |
| Biological process | GO:0044260 | cellular macromolecule metabolic process | 159 of 486 in the list | 721 of 2520 in all the Protein | 0.01552948 |
| Biological process | GO:0044085 | cellular component biogenesis | 33 of 486 in the list | 120 of 2520 in all the Protein | 0.01588186 |
| Biological process | GO:0016043 | cellular component organization | 57 of 486 in the list | 229 of 2520 in all the Protein | 0.01702142 |
| Biological process | GO:0071840 | cellular component organization or biogenesis | 63 of 486 in the list | 257 of 2520 in all the Protein | 0.01723452 |
| Biological process | GO:0006970 | response to osmotic stress | 27 of 486 in the list | 95 of 2520 in all the Protein | 0.0181827 |
| Biological process | GO:0009626 | plant-type hypersensitive response | 3 of 486 in the list | 4 of 2520 in all the Protein | 0.02444064 |
| Biological process | GO:0031123 | RNA 3'-end processing | 3 of 486 in the list | 4 of 2520 in all the Protein | 0.02444064 |
| Biological process | GO:0010016 | shoot system morphogenesis | 8 of 486 in the list | 20 of 2520 in all the Protein | 0.02574617 |
| Biological process | GO:0006820 | anion transport | 7 of 486 in the list | 17 of 2520 in all the Protein | 0.03091516 |
| Biological process | GO:0006097 | glyoxylate cycle | 2 of 486 in the list | 2 of 2520 in all the Protein | 0.03713208 |
| Biological process | GO:0006403 | RNA localization | 2 of 486 in the list | 2 of 2520 in all the Protein | 0.03713208 |
| Biological process | GO:0006405 | RNA export from nucleus | 2 of 486 in the list | 2 of 2520 in all the Protein | 0.03713208 |
| Biological process | GO:0022618 | ribonucleoprotein complex assembly | 2 of 486 in the list | 2 of 2520 in all the Protein | 0.03713208 |
| Biological process | GO:0042255 | ribosome assembly | 2 of 486 in the list | 2 of 2520 in all the Protein | 0.03713208 |
| Biological process | GO:0042256 | mature ribosome assembly | 2 of 486 in the list | 2 of 2520 in all the Protein | 0.03713208 |
| Biological process | GO:0042274 | ribosomal small subunit biogenesis | 2 of 486 in the list | 2 of 2520 in all the Protein | 0.03713208 |
| Biological process | GO:0043631 | RNA polyadenylation | 2 of 486 in the list | 2 of 2520 in all the Protein | 0.03713208 |
| Biological process | GO:0050657 | nucleic acid transport | 2 of 486 in the list | 2 of 2520 in all the Protein | 0.03713208 |
| Biological process | GO:0050658 | RNA transport | 2 of 486 in the list | 2 of 2520 in all the Protein | 0.03713208 |
| Biological process | GO:0051168 | nuclear export | 2 of 486 in the list | 2 of 2520 in all the Protein | 0.03713208 |
| Biological process | GO:0051205 | protein insertion into membrane | 2 of 486 in the list | 2 of 2520 in all the Protein | 0.03713208 |
| Biological process | GO:0051236 | establishment of RNA localization | 2 of 486 in the list | 2 of 2520 in all the Protein | 0.03713208 |
| Biological process | GO:0051668 | localization within membrane | 2 of 486 in the list | 2 of 2520 in all the Protein | 0.03713208 |
| Biological process | GO:0070925 | organelle assembly | 2 of 486 in the list | 2 of 2520 in all the Protein | 0.03713208 |
| Biological process | GO:0071826 | ribonucleoprotein complex subunit organization | 2 of 486 in the list | 2 of 2520 in all the Protein | 0.03713208 |
| Biological process | GO:0080170 | hydrogen peroxide transmembrane transport | 2 of 486 in the list | 2 of 2520 in all the Protein | 0.03713208 |
| Biological process | GO:0090304 | nucleic acid metabolic process | 44 of 486 in the list | 179 of 2520 in all the Protein | 0.04154936 |
| Biological process | GO:0044765 | single-organism transport | 54 of 486 in the list | 226 of 2520 in all the Protein | 0.04228507 |
| Biological process | GO:0009269 | response to desiccation | 5 of 486 in the list | 11 of 2520 in all the Protein | 0.0434315 |
| Biological process | GO:0043170 | macromolecule metabolic process | 181 of 486 in the list | 853 of 2520 in all the Protein | 0.04454438 |
| Biological process | GO:0009651 | response to salt stress | 24 of 486 in the list | 89 of 2520 in all the Protein | 0.04550569 |
| Biological process | GO:0001932 | regulation of protein phosphorylation | 4 of 486 in the list | 8 of 2520 in all the Protein | 0.04968156 |
| Biological process | GO:0042325 | regulation of phosphorylation | 4 of 486 in the list | 8 of 2520 in all the Protein | 0.04968156 |
| Biological process | GO:0033554 | cellular response to stress | 15 of 486 in the list | 51 of 2520 in all the Protein | 0.05228798 |
| Biological process | GO:0000186 | activation of MAPKK activity | 3 of 486 in the list | 5 of 2520 in all the Protein | 0.0524037 |
| Biological process | GO:0006556 | S-adenosylmethionine biosynthetic process | 3 of 486 in the list | 5 of 2520 in all the Protein | 0.0524037 |
| Biological process | GO:0071705 | nitrogen compound transport | 10 of 486 in the list | 31 of 2520 in all the Protein | 0.05946341 |
| Biological process | GO:0044699 | single-organism process | 162 of 486 in the list | 764 of 2520 in all the Protein | 0.0606531 |
| Biological process | GO:0006811 | ion transport | 21 of 486 in the list | 79 of 2520 in all the Protein | 0.06744802 |
| Biological process | GO:0032268 | regulation of cellular protein metabolic process | 7 of 486 in the list | 20 of 2520 in all the Protein | 0.07292093 |
| Biological process | GO:0009628 | response to abiotic stimulus | 54 of 486 in the list | 234 of 2520 in all the Protein | 0.07467295 |
| Biological process | GO:0015931 | nucleobase-containing compound transport | 4 of 486 in the list | 9 of 2520 in all the Protein | 0.07614114 |
| Biological process | GO:0006950 | response to stress | 74 of 486 in the list | 332 of 2520 in all the Protein | 0.08020163 |
| Biological process | GO:0009965 | leaf morphogenesis | 5 of 486 in the list | 13 of 2520 in all the Protein | 0.08669611 |
| Biological process | GO:0001934 | positive regulation of protein phosphorylation | 3 of 486 in the list | 6 of 2520 in all the Protein | 0.09005839 |
| Biological process | GO:0006826 | iron ion transport | 3 of 486 in the list | 6 of 2520 in all the Protein | 0.09005839 |
| Biological process | GO:0006887 | exocytosis | 3 of 486 in the list | 6 of 2520 in all the Protein | 0.09005839 |
| Biological process | GO:0010562 | positive regulation of phosphorus metabolic process | 3 of 486 in the list | 6 of 2520 in all the Protein | 0.09005839 |
| Biological process | GO:0031401 | positive regulation of protein modification process | 3 of 486 in the list | 6 of 2520 in all the Protein | 0.09005839 |
| Biological process | GO:0032147 | activation of protein kinase activity | 3 of 486 in the list | 6 of 2520 in all the Protein | 0.09005839 |
| Biological process | GO:0033674 | positive regulation of kinase activity | 3 of 486 in the list | 6 of 2520 in all the Protein | 0.09005839 |
| Biological process | GO:0042327 | positive regulation of phosphorylation | 3 of 486 in the list | 6 of 2520 in all the Protein | 0.09005839 |
| Biological process | GO:0043408 | regulation of MAPK cascade | 3 of 486 in the list | 6 of 2520 in all the Protein | 0.09005839 |
| Biological process | GO:0045860 | positive regulation of protein kinase activity | 3 of 486 in the list | 6 of 2520 in all the Protein | 0.09005839 |
| Biological process | GO:0045937 | positive regulation of phosphate metabolic process | 3 of 486 in the list | 6 of 2520 in all the Protein | 0.09005839 |
| Biological process | GO:0051347 | positive regulation of transferase activity | 3 of 486 in the list | 6 of 2520 in all the Protein | 0.09005839 |
| Biological process | GO:0009451 | RNA modification | 6 of 486 in the list | 17 of 2520 in all the Protein | 0.09099377 |
| Biological process | GO:0002376 | immune system process | 8 of 486 in the list | 25 of 2520 in all the Protein | 0.09112148 |
| Biological process | GO:0022613 | ribonucleoprotein complex biogenesis | 7 of 486 in the list | 21 of 2520 in all the Protein | 0.09198897 |
| Biological process | GO:0042254 | ribosome biogenesis | 7 of 486 in the list | 21 of 2520 in all the Protein | 0.09198897 |
| Biological process | GO:0000105 | histidine biosynthetic process | 2 of 486 in the list | 3 of 2520 in all the Protein | 0.09712148 |
| Biological process | GO:0006672 | ceramide metabolic process | 2 of 486 in the list | 3 of 2520 in all the Protein | 0.09712148 |
| Biological process | GO:0009076 | histidine family amino acid biosynthetic process | 2 of 486 in the list | 3 of 2520 in all the Protein | 0.09712148 |
| Biological process | GO:0009566 | fertilization | 2 of 486 in the list | 3 of 2520 in all the Protein | 0.09712148 |
| Biological process | GO:0009567 | double fertilization forming a zygote and endosperm | 2 of 486 in the list | 3 of 2520 in all the Protein | 0.09712148 |
| Biological process | GO:0009606 | tropism | 2 of 486 in the list | 3 of 2520 in all the Protein | 0.09712148 |
| Biological process | GO:0016137 | glycoside metabolic process | 2 of 486 in the list | 3 of 2520 in all the Protein | 0.09712148 |
| Biological process | GO:0046487 | glyoxylate metabolic process | 2 of 486 in the list | 3 of 2520 in all the Protein | 0.09712148 |
| Biological process | GO:0048598 | embryonic morphogenesis | 2 of 486 in the list | 3 of 2520 in all the Protein | 0.09712148 |
| Biological process | GO:0001510 | RNA methylation | 4 of 486 in the list | 10 of 2520 in all the Protein | 0.1082119 |
| Biological process | GO:0031399 | regulation of protein modification process | 4 of 486 in the list | 10 of 2520 in all the Protein | 0.1082119 |
| Biological process | GO:0043543 | protein acylation | 4 of 486 in the list | 10 of 2520 in all the Protein | 0.1082119 |
| Biological process | GO:0009415 | response to water stimulus | 11 of 486 in the list | 39 of 2520 in all the Protein | 0.1142076 |
| Biological process | GO:0043085 | positive regulation of catalytic activity | 5 of 486 in the list | 14 of 2520 in all the Protein | 0.1143711 |
| Biological process | GO:0044093 | positive regulation of molecular function | 5 of 486 in the list | 14 of 2520 in all the Protein | 0.1143711 |
| Biological process | GO:0045087 | innate immune response | 6 of 486 in the list | 18 of 2520 in all the Protein | 0.1152724 |
| Biological process | GO:0009059 | macromolecule biosynthetic process | 71 of 486 in the list | 327 of 2520 in all the Protein | 0.1324757 |
| Biological process | GO:0005977 | glycogen metabolic process | 3 of 486 in the list | 7 of 2520 in all the Protein | 0.1356889 |
| Biological process | GO:0005978 | glycogen biosynthetic process | 3 of 486 in the list | 7 of 2520 in all the Protein | 0.1356889 |
| Biological process | GO:0006112 | energy reserve metabolic process | 3 of 486 in the list | 7 of 2520 in all the Protein | 0.1356889 |
| Biological process | GO:0006473 | protein acetylation | 3 of 486 in the list | 7 of 2520 in all the Protein | 0.1356889 |
| Biological process | GO:0006475 | internal protein amino acid acetylation | 3 of 486 in the list | 7 of 2520 in all the Protein | 0.1356889 |
| Biological process | GO:0006816 | calcium ion transport | 3 of 486 in the list | 7 of 2520 in all the Protein | 0.1356889 |
| Biological process | GO:0006972 | hyperosmotic response | 3 of 486 in the list | 7 of 2520 in all the Protein | 0.1356889 |
| Biological process | GO:0010627 | regulation of intracellular protein kinase cascade | 3 of 486 in the list | 7 of 2520 in all the Protein | 0.1356889 |
| Biological process | GO:0015849 | organic acid transport | 3 of 486 in the list | 7 of 2520 in all the Protein | 0.1356889 |
| Biological process | GO:0016573 | histone acetylation | 3 of 486 in the list | 7 of 2520 in all the Protein | 0.1356889 |
| Biological process | GO:0018393 | internal peptidyl-lysine acetylation | 3 of 486 in the list | 7 of 2520 in all the Protein | 0.1356889 |
| Biological process | GO:0018394 | peptidyl-lysine acetylation | 3 of 486 in the list | 7 of 2520 in all the Protein | 0.1356889 |
| Biological process | GO:0034050 | host programmed cell death induced by symbiont | 3 of 486 in the list | 7 of 2520 in all the Protein | 0.1356889 |
| Biological process | GO:0043549 | regulation of kinase activity | 3 of 486 in the list | 7 of 2520 in all the Protein | 0.1356889 |
| Biological process | GO:0045859 | regulation of protein kinase activity | 3 of 486 in the list | 7 of 2520 in all the Protein | 0.1356889 |
| Biological process | GO:0046500 | S-adenosylmethionine metabolic process | 3 of 486 in the list | 7 of 2520 in all the Protein | 0.1356889 |
| Biological process | GO:0046942 | carboxylic acid transport | 3 of 486 in the list | 7 of 2520 in all the Protein | 0.1356889 |
| Biological process | GO:0051338 | regulation of transferase activity | 3 of 486 in the list | 7 of 2520 in all the Protein | 0.1356889 |
| Biological process | GO:0009414 | response to water deprivation | 10 of 486 in the list | 36 of 2520 in all the Protein | 0.1391538 |
| Biological process | GO:0006412 | translation | 51 of 486 in the list | 230 of 2520 in all the Protein | 0.1411639 |
| Biological process | GO:0006955 | immune response | 6 of 486 in the list | 19 of 2520 in all the Protein | 0.1424398 |
| Biological process | GO:0006006 | glucose metabolic process | 19 of 486 in the list | 77 of 2520 in all the Protein | 0.1426899 |
| Biological process | GO:0006397 | mRNA processing | 4 of 486 in the list | 11 of 2520 in all the Protein | 0.1452293 |
| Biological process | GO:0008643 | carbohydrate transport | 4 of 486 in the list | 11 of 2520 in all the Protein | 0.1452293 |
| Biological process | GO:0032940 | secretion by cell | 4 of 486 in the list | 11 of 2520 in all the Protein | 0.1452293 |
| Biological process | GO:0046903 | secretion | 4 of 486 in the list | 11 of 2520 in all the Protein | 0.1452293 |
| Biological process | GO:0009887 | organ morphogenesis | 5 of 486 in the list | 15 of 2520 in all the Protein | 0.1456795 |
| Biological process | GO:0009908 | flower development | 5 of 486 in the list | 15 of 2520 in all the Protein | 0.1456795 |
| Biological process | GO:0051716 | cellular response to stimulus | 36 of 486 in the list | 158 of 2520 in all the Protein | 0.1477331 |
| Biological process | GO:0006091 | generation of precursor metabolites and energy | 28 of 486 in the list | 120 of 2520 in all the Protein | 0.1509359 |
| Biological process | GO:0034645 | cellular macromolecule biosynthetic process | 68 of 486 in the list | 315 of 2520 in all the Protein | 0.1514959 |
| Biological process | GO:0048367 | shoot system development | 8 of 486 in the list | 28 of 2520 in all the Protein | 0.1553005 |
| Biological process | GO:0051246 | regulation of protein metabolic process | 7 of 486 in the list | 24 of 2520 in all the Protein | 0.1637692 |
| Biological process | GO:0048316 | seed development | 9 of 486 in the list | 33 of 2520 in all the Protein | 0.1697457 |
| Biological process | GO:0001666 | response to hypoxia | 2 of 486 in the list | 4 of 2520 in all the Protein | 0.1698023 |
| Biological process | GO:0006547 | histidine metabolic process | 2 of 486 in the list | 4 of 2520 in all the Protein | 0.1698023 |
| Biological process | GO:0006559 | L-phenylalanine catabolic process | 2 of 486 in the list | 4 of 2520 in all the Protein | 0.1698023 |
| Biological process | GO:0006904 | vesicle docking involved in exocytosis | 2 of 486 in the list | 4 of 2520 in all the Protein | 0.1698023 |
| Biological process | GO:0009075 | histidine family amino acid metabolic process | 2 of 486 in the list | 4 of 2520 in all the Protein | 0.1698023 |
| Biological process | GO:0010411 | xyloglucan metabolic process | 2 of 486 in the list | 4 of 2520 in all the Protein | 0.1698023 |
| Biological process | GO:0035335 | peptidyl-tyrosine dephosphorylation | 2 of 486 in the list | 4 of 2520 in all the Protein | 0.1698023 |
| Biological process | GO:0036293 | response to decreased oxygen levels | 2 of 486 in the list | 4 of 2520 in all the Protein | 0.1698023 |
| Biological process | GO:0042744 | hydrogen peroxide catabolic process | 2 of 486 in the list | 4 of 2520 in all the Protein | 0.1698023 |
| Biological process | GO:0052803 | imidazole-containing compound metabolic process | 2 of 486 in the list | 4 of 2520 in all the Protein | 0.1698023 |
| Biological process | GO:0070301 | cellular response to hydrogen peroxide | 2 of 486 in the list | 4 of 2520 in all the Protein | 0.1698023 |
| Biological process | GO:0070482 | response to oxygen levels | 2 of 486 in the list | 4 of 2520 in all the Protein | 0.1698023 |
| Biological process | GO:0016044 | cellular membrane organization | 6 of 486 in the list | 20 of 2520 in all the Protein | 0.1722336 |
| Biological process | GO:0061024 | membrane organization | 6 of 486 in the list | 20 of 2520 in all the Protein | 0.1722336 |
| Biological process | GO:0070887 | cellular response to chemical stimulus | 12 of 486 in the list | 47 of 2520 in all the Protein | 0.1796773 |
| Biological process | GO:0016071 | mRNA metabolic process | 5 of 486 in the list | 16 of 2520 in all the Protein | 0.1801749 |
| Biological process | GO:0042545 | cell wall modification | 5 of 486 in the list | 16 of 2520 in all the Protein | 0.1801749 |
| Biological process | GO:0043414 | macromolecule methylation | 5 of 486 in the list | 16 of 2520 in all the Protein | 0.1801749 |
| Biological process | GO:0009793 | embryo development ending in seed dormancy | 8 of 486 in the list | 29 of 2520 in all the Protein | 0.1804949 |
| Biological process | GO:0019318 | hexose metabolic process | 22 of 486 in the list | 94 of 2520 in all the Protein | 0.183088 |
| Biological process | GO:0006364 | rRNA processing | 4 of 486 in the list | 12 of 2520 in all the Protein | 0.1863501 |
| Biological process | GO:0051258 | protein polymerization | 4 of 486 in the list | 12 of 2520 in all the Protein | 0.1863501 |
| Biological process | GO:0009637 | response to blue light | 3 of 486 in the list | 8 of 2520 in all the Protein | 0.1872933 |
| Biological process | GO:0009668 | plastid membrane organization | 3 of 486 in the list | 8 of 2520 in all the Protein | 0.1872933 |
| Biological process | GO:0010027 | thylakoid membrane organization | 3 of 486 in the list | 8 of 2520 in all the Protein | 0.1872933 |
| Biological process | GO:0015748 | organophosphate ester transport | 3 of 486 in the list | 8 of 2520 in all the Protein | 0.1872933 |
| Biological process | GO:0034614 | cellular response to reactive oxygen species | 3 of 486 in the list | 8 of 2520 in all the Protein | 0.1872933 |
| Biological process | GO:1901659 | glycosyl compound biosynthetic process | 16 of 486 in the list | 66 of 2520 in all the Protein | 0.1883588 |
| Biological process | GO:0044763 | single-organism cellular process | 133 of 486 in the list | 648 of 2520 in all the Protein | 0.1918448 |
| Biological process | GO:0009790 | embryo development | 10 of 486 in the list | 39 of 2520 in all the Protein | 0.2049872 |
| Biological process | GO:0042451 | purine nucleoside biosynthetic process | 13 of 486 in the list | 53 of 2520 in all the Protein | 0.2076844 |
| Biological process | GO:0046129 | purine ribonucleoside biosynthetic process | 13 of 486 in the list | 53 of 2520 in all the Protein | 0.2076844 |
| Biological process | GO:0055085 | transmembrane transport | 23 of 486 in the list | 101 of 2520 in all the Protein | 0.2155162 |
| Biological process | GO:0006833 | water transport | 5 of 486 in the list | 17 of 2520 in all the Protein | 0.2173337 |
| Biological process | GO:0009664 | plant-type cell wall organization | 5 of 486 in the list | 17 of 2520 in all the Protein | 0.2173337 |
| Biological process | GO:0035295 | tube development | 5 of 486 in the list | 17 of 2520 in all the Protein | 0.2173337 |
| Biological process | GO:0042044 | fluid transport | 5 of 486 in the list | 17 of 2520 in all the Protein | 0.2173337 |
| Biological process | GO:0048868 | pollen tube development | 5 of 486 in the list | 17 of 2520 in all the Protein | 0.2173337 |
| Biological process | GO:0010154 | fruit development | 9 of 486 in the list | 35 of 2520 in all the Protein | 0.2195228 |
| Biological process | GO:0009163 | nucleoside biosynthetic process | 15 of 486 in the list | 63 of 2520 in all the Protein | 0.2196394 |
| Biological process | GO:0042455 | ribonucleoside biosynthetic process | 15 of 486 in the list | 63 of 2520 in all the Protein | 0.2196394 |
| Biological process | GO:0009250 | glucan biosynthetic process | 10 of 486 in the list | 40 of 2520 in all the Protein | 0.2294414 |
| Biological process | GO:0008219 | cell death | 4 of 486 in the list | 13 of 2520 in all the Protein | 0.2306407 |
| Biological process | GO:0016265 | death | 4 of 486 in the list | 13 of 2520 in all the Protein | 0.2306407 |
| Biological process | GO:0010467 | gene expression | 62 of 486 in the list | 295 of 2520 in all the Protein | 0.2328886 |
| Biological process | GO:0009733 | response to auxin stimulus | 6 of 486 in the list | 22 of 2520 in all the Protein | 0.2383681 |
| Biological process | GO:0006534 | cysteine metabolic process | 3 of 486 in the list | 9 of 2520 in all the Protein | 0.2428693 |
| Biological process | GO:0006664 | glycolipid metabolic process | 3 of 486 in the list | 9 of 2520 in all the Protein | 0.2428693 |
| Biological process | GO:0009734 | auxin mediated signaling pathway | 3 of 486 in the list | 9 of 2520 in all the Protein | 0.2428693 |
| Biological process | GO:0012501 | programmed cell death | 3 of 486 in the list | 9 of 2520 in all the Protein | 0.2428693 |
| Biological process | GO:0015711 | organic anion transport | 3 of 486 in the list | 9 of 2520 in all the Protein | 0.2428693 |
| Biological process | GO:0019344 | cysteine biosynthetic process | 3 of 486 in the list | 9 of 2520 in all the Protein | 0.2428693 |
| Biological process | GO:0032270 | positive regulation of cellular protein metabolic process | 3 of 486 in the list | 9 of 2520 in all the Protein | 0.2428693 |
| Biological process | GO:0034599 | cellular response to oxidative stress | 3 of 486 in the list | 9 of 2520 in all the Protein | 0.2428693 |
| Biological process | GO:0051247 | positive regulation of protein metabolic process | 3 of 486 in the list | 9 of 2520 in all the Protein | 0.2428693 |
| Biological process | GO:0070838 | divalent metal ion transport | 3 of 486 in the list | 9 of 2520 in all the Protein | 0.2428693 |
| Biological process | GO:0071365 | cellular response to auxin stimulus | 3 of 486 in the list | 9 of 2520 in all the Protein | 0.2428693 |
| Biological process | GO:0072511 | divalent inorganic cation transport | 3 of 486 in the list | 9 of 2520 in all the Protein | 0.2428693 |
| Biological process | GO:0006007 | glucose catabolic process | 16 of 486 in the list | 69 of 2520 in all the Protein | 0.2436822 |
| Biological process | GO:0006812 | cation transport | 16 of 486 in the list | 69 of 2520 in all the Protein | 0.2436822 |
| Biological process | GO:0019320 | hexose catabolic process | 16 of 486 in the list | 69 of 2520 in all the Protein | 0.2436822 |
| Biological process | GO:0044724 | single-organism carbohydrate catabolic process | 16 of 486 in the list | 69 of 2520 in all the Protein | 0.2436822 |
| Biological process | GO:0046365 | monosaccharide catabolic process | 16 of 486 in the list | 69 of 2520 in all the Protein | 0.2436822 |
| Biological process | GO:0016052 | carbohydrate catabolic process | 17 of 486 in the list | 74 of 2520 in all the Protein | 0.2475027 |
| Biological process | GO:0001676 | long-chain fatty acid metabolic process | 2 of 486 in the list | 5 of 2520 in all the Protein | 0.2480681 |
| Biological process | GO:0006207 | 'de novo' pyrimidine nucleobase biosynthetic process | 2 of 486 in the list | 5 of 2520 in all the Protein | 0.2480681 |
| Biological process | GO:0006553 | lysine metabolic process | 2 of 486 in the list | 5 of 2520 in all the Protein | 0.2480681 |
| Biological process | GO:0006558 | L-phenylalanine metabolic process | 2 of 486 in the list | 5 of 2520 in all the Protein | 0.2480681 |
| Biological process | GO:0009085 | lysine biosynthetic process | 2 of 486 in the list | 5 of 2520 in all the Protein | 0.2480681 |
| Biological process | GO:0009089 | lysine biosynthetic process via diaminopimelate | 2 of 486 in the list | 5 of 2520 in all the Protein | 0.2480681 |
| Biological process | GO:0009751 | response to salicylic acid stimulus | 2 of 486 in the list | 5 of 2520 in all the Protein | 0.2480681 |
| Biological process | GO:0009911 | positive regulation of flower development | 2 of 486 in the list | 5 of 2520 in all the Protein | 0.2480681 |
| Biological process | GO:0010393 | galacturonan metabolic process | 2 of 486 in the list | 5 of 2520 in all the Protein | 0.2480681 |
| Biological process | GO:0015865 | purine nucleotide transport | 2 of 486 in the list | 5 of 2520 in all the Protein | 0.2480681 |
| Biological process | GO:0022406 | membrane docking | 2 of 486 in the list | 5 of 2520 in all the Protein | 0.2480681 |
| Biological process | GO:0042439 | ethanolamine-containing compound metabolic process | 2 of 486 in the list | 5 of 2520 in all the Protein | 0.2480681 |
| Biological process | GO:0042743 | hydrogen peroxide metabolic process | 2 of 486 in the list | 5 of 2520 in all the Protein | 0.2480681 |
| Biological process | GO:0043604 | amide biosynthetic process | 2 of 486 in the list | 5 of 2520 in all the Protein | 0.2480681 |
| Biological process | GO:0045488 | pectin metabolic process | 2 of 486 in the list | 5 of 2520 in all the Protein | 0.2480681 |
| Biological process | GO:0046470 | phosphatidylcholine metabolic process | 2 of 486 in the list | 5 of 2520 in all the Protein | 0.2480681 |
| Biological process | GO:0048278 | vesicle docking | 2 of 486 in the list | 5 of 2520 in all the Protein | 0.2480681 |
| Biological process | GO:0048582 | positive regulation of post-embryonic development | 2 of 486 in the list | 5 of 2520 in all the Protein | 0.2480681 |
| Biological process | GO:0051240 | positive regulation of multicellular organismal process | 2 of 486 in the list | 5 of 2520 in all the Protein | 0.2480681 |
| Biological process | GO:0051552 | flavone metabolic process | 2 of 486 in the list | 5 of 2520 in all the Protein | 0.2480681 |
| Biological process | GO:0051553 | flavone biosynthetic process | 2 of 486 in the list | 5 of 2520 in all the Protein | 0.2480681 |
| Biological process | GO:2000243 | positive regulation of reproductive process | 2 of 486 in the list | 5 of 2520 in all the Protein | 0.2480681 |
| Biological process | GO:0044237 | cellular metabolic process | 274 of 486 in the list | 1384 of 2520 in all the Protein | 0.2521748 |
| Biological process | GO:0006725 | cellular aromatic compound metabolic process | 93 of 486 in the list | 454 of 2520 in all the Protein | 0.2564635 |
| Biological process | GO:0006576 | cellular biogenic amine metabolic process | 5 of 486 in the list | 18 of 2520 in all the Protein | 0.2565881 |
| Biological process | GO:0044106 | cellular amine metabolic process | 5 of 486 in the list | 18 of 2520 in all the Protein | 0.2565881 |
| Biological process | GO:0009072 | aromatic amino acid family metabolic process | 6 of 486 in the list | 23 of 2520 in all the Protein | 0.2739515 |
| Biological process | GO:0048366 | leaf development | 6 of 486 in the list | 23 of 2520 in all the Protein | 0.2739515 |
| Biological process | GO:0048827 | phyllome development | 6 of 486 in the list | 23 of 2520 in all the Protein | 0.2739515 |
| Biological process | GO:0051234 | establishment of localization | 75 of 486 in the list | 365 of 2520 in all the Protein | 0.2756046 |
| Biological process | GO:0006928 | cellular component movement | 4 of 486 in the list | 14 of 2520 in all the Protein | 0.2771486 |
| Biological process | GO:0007018 | microtubule-based movement | 4 of 486 in the list | 14 of 2520 in all the Protein | 0.2771486 |
| Biological process | GO:0016072 | rRNA metabolic process | 4 of 486 in the list | 14 of 2520 in all the Protein | 0.2771486 |
| Biological process | GO:0030244 | cellulose biosynthetic process | 4 of 486 in the list | 14 of 2520 in all the Protein | 0.2771486 |
| Biological process | GO:0044036 | cell wall macromolecule metabolic process | 4 of 486 in the list | 14 of 2520 in all the Protein | 0.2771486 |
| Biological process | GO:0050896 | response to stimulus | 116 of 486 in the list | 574 of 2520 in all the Protein | 0.2801928 |
| Biological process | GO:0006096 | glycolysis | 10 of 486 in the list | 42 of 2520 in all the Protein | 0.2813349 |
| Biological process | GO:0015980 | energy derivation by oxidation of organic compounds | 10 of 486 in the list | 42 of 2520 in all the Protein | 0.2813349 |
| Biological process | GO:0009060 | aerobic respiration | 7 of 486 in the list | 28 of 2520 in all the Protein | 0.2863747 |
| Biological process | GO:0009698 | phenylpropanoid metabolic process | 7 of 486 in the list | 28 of 2520 in all the Protein | 0.2863747 |
| Biological process | GO:0045229 | external encapsulating structure organization | 7 of 486 in the list | 28 of 2520 in all the Protein | 0.2863747 |
| Biological process | GO:0055114 | oxidation-reduction process | 91 of 486 in the list | 448 of 2520 in all the Protein | 0.2920061 |
| Biological process | GO:0006139 | nucleobase-containing compound metabolic process | 77 of 486 in the list | 377 of 2520 in all the Protein | 0.2932065 |
| Biological process | GO:0051179 | localization | 76 of 486 in the list | 372 of 2520 in all the Protein | 0.2938996 |
| Biological process | GO:0014070 | response to organic cyclic compound | 5 of 486 in the list | 19 of 2520 in all the Protein | 0.2973553 |
| Biological process | GO:0000041 | transition metal ion transport | 3 of 486 in the list | 10 of 2520 in all the Protein | 0.3005796 |
| Biological process | GO:0000165 | MAPK cascade | 3 of 486 in the list | 10 of 2520 in all the Protein | 0.3005796 |
| Biological process | GO:0006417 | regulation of translation | 3 of 486 in the list | 10 of 2520 in all the Protein | 0.3005796 |
| Biological process | GO:0007243 | intracellular protein kinase cascade | 3 of 486 in the list | 10 of 2520 in all the Protein | 0.3005796 |
| Biological process | GO:0018205 | peptidyl-lysine modification | 3 of 486 in the list | 10 of 2520 in all the Protein | 0.3005796 |
| Biological process | GO:0034976 | response to endoplasmic reticulum stress | 3 of 486 in the list | 10 of 2520 in all the Protein | 0.3005796 |
| Biological process | GO:0042401 | cellular biogenic amine biosynthetic process | 3 of 486 in the list | 10 of 2520 in all the Protein | 0.3005796 |
| Biological process | GO:0048438 | floral whorl development | 3 of 486 in the list | 10 of 2520 in all the Protein | 0.3005796 |
| Biological process | GO:0006810 | transport | 74 of 486 in the list | 363 of 2520 in all the Protein | 0.3050312 |
| Biological process | GO:0006099 | tricarboxylic acid cycle | 6 of 486 in the list | 24 of 2520 in all the Protein | 0.310679 |
| Biological process | GO:0009987 | cellular process | 336 of 486 in the list | 1716 of 2520 in all the Protein | 0.3118727 |
| Biological process | GO:0006073 | cellular glucan metabolic process | 12 of 486 in the list | 53 of 2520 in all the Protein | 0.3166769 |
| Biological process | GO:0044042 | glucan metabolic process | 12 of 486 in the list | 53 of 2520 in all the Protein | 0.3166769 |
| Biological process | GO:0048608 | reproductive structure development | 13 of 486 in the list | 58 of 2520 in all the Protein | 0.3196821 |
| Biological process | GO:0061458 | reproductive system development | 13 of 486 in the list | 58 of 2520 in all the Protein | 0.3196821 |
| Biological process | GO:0006414 | translational elongation | 7 of 486 in the list | 29 of 2520 in all the Protein | 0.3200637 |
| Biological process | GO:0030001 | metal ion transport | 7 of 486 in the list | 29 of 2520 in all the Protein | 0.3200637 |
| Biological process | GO:0006662 | glycerol ether metabolic process | 4 of 486 in the list | 15 of 2520 in all the Protein | 0.3249555 |
| Biological process | GO:0018904 | ether metabolic process | 4 of 486 in the list | 15 of 2520 in all the Protein | 0.3249555 |
| Biological process | GO:0019220 | regulation of phosphate metabolic process | 4 of 486 in the list | 15 of 2520 in all the Protein | 0.3249555 |
| Biological process | GO:0051174 | regulation of phosphorus metabolic process | 4 of 486 in the list | 15 of 2520 in all the Protein | 0.3249555 |
| Biological process | GO:0006754 | ATP biosynthetic process | 8 of 486 in the list | 34 of 2520 in all the Protein | 0.3269618 |
| Biological process | GO:0045454 | cell redox homeostasis | 8 of 486 in the list | 34 of 2520 in all the Protein | 0.3269618 |
| Biological process | GO:0000023 | maltose metabolic process | 2 of 486 in the list | 6 of 2520 in all the Protein | 0.3270729 |
| Biological process | GO:0006022 | aminoglycan metabolic process | 2 of 486 in the list | 6 of 2520 in all the Protein | 0.3270729 |
| Biological process | GO:0006839 | mitochondrial transport | 2 of 486 in the list | 6 of 2520 in all the Protein | 0.3270729 |
| Biological process | GO:0006862 | nucleotide transport | 2 of 486 in the list | 6 of 2520 in all the Protein | 0.3270729 |
| Biological process | GO:0009074 | aromatic amino acid family catabolic process | 2 of 486 in the list | 6 of 2520 in all the Protein | 0.3270729 |
| Biological process | GO:0009627 | systemic acquired resistance | 2 of 486 in the list | 6 of 2520 in all the Protein | 0.3270729 |
| Biological process | GO:0009648 | photoperiodism | 2 of 486 in the list | 6 of 2520 in all the Protein | 0.3270729 |
| Biological process | GO:0010410 | hemicellulose metabolic process | 2 of 486 in the list | 6 of 2520 in all the Protein | 0.3270729 |
| Biological process | GO:0015698 | inorganic anion transport | 2 of 486 in the list | 6 of 2520 in all the Protein | 0.3270729 |
| Biological process | GO:0019856 | pyrimidine nucleobase biosynthetic process | 2 of 486 in the list | 6 of 2520 in all the Protein | 0.3270729 |
| Biological process | GO:0046451 | diaminopimelate metabolic process | 2 of 486 in the list | 6 of 2520 in all the Protein | 0.3270729 |
| Biological process | GO:0048467 | gynoecium development | 2 of 486 in the list | 6 of 2520 in all the Protein | 0.3270729 |
| Biological process | GO:0051094 | positive regulation of developmental process | 2 of 486 in the list | 6 of 2520 in all the Protein | 0.3270729 |
| Biological process | GO:0070646 | protein modification by small protein removal | 2 of 486 in the list | 6 of 2520 in all the Protein | 0.3270729 |
| Biological process | GO:0043412 | macromolecule modification | 41 of 486 in the list | 198 of 2520 in all the Protein | 0.3273447 |
| Biological process | GO:0044262 | cellular carbohydrate metabolic process | 19 of 486 in the list | 88 of 2520 in all the Protein | 0.3293591 |
| Biological process | GO:0044267 | cellular protein metabolic process | 106 of 486 in the list | 529 of 2520 in all the Protein | 0.3309845 |
| Biological process | GO:0019725 | cellular homeostasis | 10 of 486 in the list | 44 of 2520 in all the Protein | 0.3362063 |
| Biological process | GO:0070647 | protein modification by small protein conjugation or removal | 5 of 486 in the list | 20 of 2520 in all the Protein | 0.3390625 |
| Biological process | GO:0044723 | single-organism carbohydrate metabolic process | 38 of 486 in the list | 184 of 2520 in all the Protein | 0.3426891 |
| Biological process | GO:0032259 | methylation | 15 of 486 in the list | 69 of 2520 in all the Protein | 0.346563 |
| Biological process | GO:0071555 | cell wall organization | 6 of 486 in the list | 25 of 2520 in all the Protein | 0.3481499 |
| Biological process | GO:0009108 | coenzyme biosynthetic process | 7 of 486 in the list | 30 of 2520 in all the Protein | 0.3543731 |
| Biological process | GO:0006869 | lipid transport | 3 of 486 in the list | 11 of 2520 in all the Protein | 0.358836 |
| Biological process | GO:0009267 | cellular response to starvation | 3 of 486 in the list | 11 of 2520 in all the Protein | 0.358836 |
| Biological process | GO:0010876 | lipid localization | 3 of 486 in the list | 11 of 2520 in all the Protein | 0.358836 |
| Biological process | GO:0042594 | response to starvation | 3 of 486 in the list | 11 of 2520 in all the Protein | 0.358836 |
| Biological process | GO:0034637 | cellular carbohydrate biosynthetic process | 12 of 486 in the list | 55 of 2520 in all the Protein | 0.3673547 |
| Biological process | GO:0009266 | response to temperature stimulus | 19 of 486 in the list | 90 of 2520 in all the Protein | 0.3691575 |
| Biological process | GO:0006281 | DNA repair | 4 of 486 in the list | 16 of 2520 in all the Protein | 0.3732157 |
| Biological process | GO:0010604 | positive regulation of macromolecule metabolic process | 4 of 486 in the list | 16 of 2520 in all the Protein | 0.3732157 |
| Biological process | GO:0016570 | histone modification | 4 of 486 in the list | 16 of 2520 in all the Protein | 0.3732157 |
| Biological process | GO:0030243 | cellulose metabolic process | 4 of 486 in the list | 16 of 2520 in all the Protein | 0.3732157 |
| Biological process | GO:0034470 | ncRNA processing | 4 of 486 in the list | 16 of 2520 in all the Protein | 0.3732157 |
| Biological process | GO:0042398 | cellular modified amino acid biosynthetic process | 4 of 486 in the list | 16 of 2520 in all the Protein | 0.3732157 |
| Biological process | GO:0009699 | phenylpropanoid biosynthetic process | 5 of 486 in the list | 21 of 2520 in all the Protein | 0.3811659 |
| Biological process | GO:0009856 | pollination | 5 of 486 in the list | 21 of 2520 in all the Protein | 0.3811659 |
| Biological process | GO:0044703 | multi-organism reproductive process | 5 of 486 in the list | 21 of 2520 in all the Protein | 0.3811659 |
| Biological process | GO:0044706 | multi-multicellular organism process | 5 of 486 in the list | 21 of 2520 in all the Protein | 0.3811659 |
| Biological process | GO:0005996 | monosaccharide metabolic process | 22 of 486 in the list | 106 of 2520 in all the Protein | 0.3868038 |
| Biological process | GO:0034641 | cellular nitrogen compound metabolic process | 89 of 486 in the list | 448 of 2520 in all the Protein | 0.3877007 |
| Biological process | GO:0003006 | developmental process involved in reproduction | 15 of 486 in the list | 71 of 2520 in all the Protein | 0.3921158 |
| Biological process | GO:0072522 | purine-containing compound biosynthetic process | 14 of 486 in the list | 66 of 2520 in all the Protein | 0.3925903 |
| Biological process | GO:0009119 | ribonucleoside metabolic process | 28 of 486 in the list | 137 of 2520 in all the Protein | 0.3977369 |
| Biological process | GO:0042278 | purine nucleoside metabolic process | 26 of 486 in the list | 127 of 2520 in all the Protein | 0.4003537 |
| Biological process | GO:0046128 | purine ribonucleoside metabolic process | 26 of 486 in the list | 127 of 2520 in all the Protein | 0.4003537 |
| Biological process | GO:1901360 | organic cyclic compound metabolic process | 94 of 486 in the list | 475 of 2520 in all the Protein | 0.4004977 |
| Biological process | GO:0006541 | glutamine metabolic process | 2 of 486 in the list | 7 of 2520 in all the Protein | 0.4036265 |
| Biological process | GO:0006595 | polyamine metabolic process | 2 of 486 in the list | 7 of 2520 in all the Protein | 0.4036265 |
| Biological process | GO:0006596 | polyamine biosynthetic process | 2 of 486 in the list | 7 of 2520 in all the Protein | 0.4036265 |
| Biological process | GO:0009744 | response to sucrose stimulus | 2 of 486 in the list | 7 of 2520 in all the Protein | 0.4036265 |
| Biological process | GO:0010114 | response to red light | 2 of 486 in the list | 7 of 2520 in all the Protein | 0.4036265 |
| Biological process | GO:0010383 | cell wall polysaccharide metabolic process | 2 of 486 in the list | 7 of 2520 in all the Protein | 0.4036265 |
| Biological process | GO:0034285 | response to disaccharide stimulus | 2 of 486 in the list | 7 of 2520 in all the Protein | 0.4036265 |
| Biological process | GO:1901657 | glycosyl compound metabolic process | 30 of 486 in the list | 148 of 2520 in all the Protein | 0.411364 |
| Biological process | GO:0006913 | nucleocytoplasmic transport | 3 of 486 in the list | 12 of 2520 in all the Protein | 0.4163313 |
| Biological process | GO:0009814 | defense response, incompatible interaction | 3 of 486 in the list | 12 of 2520 in all the Protein | 0.4163313 |
| Biological process | GO:0030029 | actin filament-based process | 3 of 486 in the list | 12 of 2520 in all the Protein | 0.4163313 |
| Biological process | GO:0051169 | nuclear transport | 3 of 486 in the list | 12 of 2520 in all the Protein | 0.4163313 |
| Biological process | GO:0072593 | reactive oxygen species metabolic process | 3 of 486 in the list | 12 of 2520 in all the Protein | 0.4163313 |
| Biological process | GO:0006650 | glycerophospholipid metabolic process | 4 of 486 in the list | 17 of 2520 in all the Protein | 0.4211794 |
| Biological process | GO:0006974 | response to DNA damage stimulus | 4 of 486 in the list | 17 of 2520 in all the Protein | 0.4211794 |
| Biological process | GO:0046486 | glycerolipid metabolic process | 4 of 486 in the list | 17 of 2520 in all the Protein | 0.4211794 |
| Biological process | GO:0033692 | cellular polysaccharide biosynthetic process | 10 of 486 in the list | 47 of 2520 in all the Protein | 0.4213371 |
| Biological process | GO:0071554 | cell wall organization or biogenesis | 10 of 486 in the list | 47 of 2520 in all the Protein | 0.4213371 |
| Biological process | GO:0006511 | ubiquitin-dependent protein catabolic process | 9 of 486 in the list | 42 of 2520 in all the Protein | 0.4223346 |
| Biological process | GO:0009737 | response to abscisic acid stimulus | 9 of 486 in the list | 42 of 2520 in all the Protein | 0.4223346 |
| Biological process | GO:0019941 | modification-dependent protein catabolic process | 9 of 486 in the list | 42 of 2520 in all the Protein | 0.4223346 |
| Biological process | GO:0043632 | modification-dependent macromolecule catabolic process | 9 of 486 in the list | 42 of 2520 in all the Protein | 0.4223346 |
| Biological process | GO:0005984 | disaccharide metabolic process | 5 of 486 in the list | 22 of 2520 in all the Protein | 0.4231653 |
| Biological process | GO:0009657 | plastid organization | 5 of 486 in the list | 22 of 2520 in all the Protein | 0.4231653 |
| Biological process | GO:0009719 | response to endogenous stimulus | 18 of 486 in the list | 88 of 2520 in all the Protein | 0.4321135 |
| Biological process | GO:1901136 | carbohydrate derivative catabolic process | 17 of 486 in the list | 83 of 2520 in all the Protein | 0.4340941 |
| Biological process | GO:0006468 | protein phosphorylation | 16 of 486 in the list | 78 of 2520 in all the Protein | 0.4361291 |
| Biological process | GO:0006952 | defense response | 16 of 486 in the list | 78 of 2520 in all the Protein | 0.4361291 |
| Biological process | GO:1901658 | glycosyl compound catabolic process | 16 of 486 in the list | 78 of 2520 in all the Protein | 0.4361291 |
| Biological process | GO:1901700 | response to oxygen-containing compound | 24 of 486 in the list | 119 of 2520 in all the Protein | 0.43936 |
| Biological process | GO:0009314 | response to radiation | 13 of 486 in the list | 63 of 2520 in all the Protein | 0.4426169 |
| Biological process | GO:0009416 | response to light stimulus | 13 of 486 in the list | 63 of 2520 in all the Protein | 0.4426169 |
| Biological process | GO:0044238 | primary metabolic process | 279 of 486 in the list | 1437 of 2520 in all the Protein | 0.4452429 |
| Biological process | GO:0009409 | response to cold | 11 of 486 in the list | 53 of 2520 in all the Protein | 0.4473367 |
| Biological process | GO:0000271 | polysaccharide biosynthetic process | 10 of 486 in the list | 48 of 2520 in all the Protein | 0.4498459 |
| Biological process | GO:0097305 | response to alcohol | 10 of 486 in the list | 48 of 2520 in all the Protein | 0.4498459 |
| Biological process | GO:0006790 | sulfur compound metabolic process | 9 of 486 in the list | 43 of 2520 in all the Protein | 0.4524735 |
| Biological process | GO:0009145 | purine nucleoside triphosphate biosynthetic process | 8 of 486 in the list | 38 of 2520 in all the Protein | 0.4552383 |
| Biological process | GO:0009201 | ribonucleoside triphosphate biosynthetic process | 8 of 486 in the list | 38 of 2520 in all the Protein | 0.4552383 |
| Biological process | GO:0009206 | purine ribonucleoside triphosphate biosynthetic process | 8 of 486 in the list | 38 of 2520 in all the Protein | 0.4552383 |
| Biological process | GO:0009308 | amine metabolic process | 7 of 486 in the list | 33 of 2520 in all the Protein | 0.4581632 |
| Biological process | GO:0009605 | response to external stimulus | 7 of 486 in the list | 33 of 2520 in all the Protein | 0.4581632 |
| Biological process | GO:0009791 | post-embryonic development | 16 of 486 in the list | 79 of 2520 in all the Protein | 0.458453 |
| Biological process | GO:0006575 | cellular modified amino acid metabolic process | 6 of 486 in the list | 28 of 2520 in all the Protein | 0.4612769 |
| Biological process | GO:0009311 | oligosaccharide metabolic process | 6 of 486 in the list | 28 of 2520 in all the Protein | 0.4612769 |
| Biological process | GO:0022414 | reproductive process | 15 of 486 in the list | 74 of 2520 in all the Protein | 0.4612869 |
| Biological process | GO:0019438 | aromatic compound biosynthetic process | 35 of 486 in the list | 177 of 2520 in all the Protein | 0.4644261 |
| Biological process | GO:0000096 | sulfur amino acid metabolic process | 5 of 486 in the list | 23 of 2520 in all the Protein | 0.4646135 |
| Biological process | GO:0000302 | response to reactive oxygen species | 5 of 486 in the list | 23 of 2520 in all the Protein | 0.4646135 |
| Biological process | GO:0010608 | posttranscriptional regulation of gene expression | 4 of 486 in the list | 18 of 2520 in all the Protein | 0.4682055 |
| Biological process | GO:0016311 | dephosphorylation | 4 of 486 in the list | 18 of 2520 in all the Protein | 0.4682055 |
| Biological process | GO:0016569 | covalent chromatin modification | 4 of 486 in the list | 18 of 2520 in all the Protein | 0.4682055 |
| Biological process | GO:0006108 | malate metabolic process | 3 of 486 in the list | 13 of 2520 in all the Protein | 0.4720385 |
| Biological process | GO:0006470 | protein dephosphorylation | 3 of 486 in the list | 13 of 2520 in all the Protein | 0.4720385 |
| Biological process | GO:0007389 | pattern specification process | 3 of 486 in the list | 13 of 2520 in all the Protein | 0.4720385 |
| Biological process | GO:0009073 | aromatic amino acid family biosynthetic process | 3 of 486 in the list | 13 of 2520 in all the Protein | 0.4720385 |
| Biological process | GO:0009808 | lignin metabolic process | 3 of 486 in the list | 13 of 2520 in all the Protein | 0.4720385 |
| Biological process | GO:0031667 | response to nutrient levels | 3 of 486 in the list | 13 of 2520 in all the Protein | 0.4720385 |
| Biological process | GO:0031669 | cellular response to nutrient levels | 3 of 486 in the list | 13 of 2520 in all the Protein | 0.4720385 |
| Biological process | GO:0042542 | response to hydrogen peroxide | 3 of 486 in the list | 13 of 2520 in all the Protein | 0.4720385 |
| Biological process | GO:0042886 | amide transport | 3 of 486 in the list | 13 of 2520 in all the Protein | 0.4720385 |
| Biological process | GO:1901698 | response to nitrogen compound | 3 of 486 in the list | 13 of 2520 in all the Protein | 0.4720385 |
| Biological process | GO:0005985 | sucrose metabolic process | 2 of 486 in the list | 8 of 2520 in all the Protein | 0.4757376 |
| Biological process | GO:0006206 | pyrimidine nucleobase metabolic process | 2 of 486 in the list | 8 of 2520 in all the Protein | 0.4757376 |
| Biological process | GO:0007031 | peroxisome organization | 2 of 486 in the list | 8 of 2520 in all the Protein | 0.4757376 |
| Biological process | GO:0009886 | post-embryonic morphogenesis | 2 of 486 in the list | 8 of 2520 in all the Protein | 0.4757376 |
| Biological process | GO:0010218 | response to far red light | 2 of 486 in the list | 8 of 2520 in all the Protein | 0.4757376 |
| Biological process | GO:0016036 | cellular response to phosphate starvation | 2 of 486 in the list | 8 of 2520 in all the Protein | 0.4757376 |
| Biological process | GO:0019953 | sexual reproduction | 2 of 486 in the list | 8 of 2520 in all the Protein | 0.4757376 |
| Biological process | GO:0044702 | single organism reproductive process | 2 of 486 in the list | 8 of 2520 in all the Protein | 0.4757376 |
| Biological process | GO:0046467 | membrane lipid biosynthetic process | 2 of 486 in the list | 8 of 2520 in all the Protein | 0.4757376 |
| Biological process | GO:0048646 | anatomical structure formation involved in morphogenesis | 2 of 486 in the list | 8 of 2520 in all the Protein | 0.4757376 |
| Biological process | GO:0090305 | nucleic acid phosphodiester bond hydrolysis | 2 of 486 in the list | 8 of 2520 in all the Protein | 0.4757376 |
| Biological process | GO:0010033 | response to organic substance | 24 of 486 in the list | 121 of 2520 in all the Protein | 0.4757951 |
| Biological process | GO:0042592 | homeostatic process | 10 of 486 in the list | 49 of 2520 in all the Protein | 0.4781771 |
| Biological process | GO:1901137 | carbohydrate derivative biosynthetic process | 21 of 486 in the list | 106 of 2520 in all the Protein | 0.4848037 |
| Biological process | GO:0009142 | nucleoside triphosphate biosynthetic process | 8 of 486 in the list | 39 of 2520 in all the Protein | 0.4869952 |
| Biological process | GO:0019748 | secondary metabolic process | 8 of 486 in the list | 39 of 2520 in all the Protein | 0.4869952 |
| Biological process | GO:0009058 | biosynthetic process | 133 of 486 in the list | 686 of 2520 in all the Protein | 0.4888398 |
| Biological process | GO:0006807 | nitrogen compound metabolic process | 109 of 486 in the list | 562 of 2520 in all the Protein | 0.4917234 |
| Biological process | GO:0005976 | polysaccharide metabolic process | 13 of 486 in the list | 65 of 2520 in all the Protein | 0.4920509 |
| Biological process | GO:0044272 | sulfur compound biosynthetic process | 7 of 486 in the list | 34 of 2520 in all the Protein | 0.4921783 |
| Biological process | GO:0044264 | cellular polysaccharide metabolic process | 12 of 486 in the list | 60 of 2520 in all the Protein | 0.4963791 |
| Biological process | GO:0046034 | ATP metabolic process | 12 of 486 in the list | 60 of 2520 in all the Protein | 0.4963791 |
| Biological process | GO:0044249 | cellular biosynthetic process | 122 of 486 in the list | 630 of 2520 in all the Protein | 0.4976153 |
| Biological process | GO:0006633 | fatty acid biosynthetic process | 6 of 486 in the list | 29 of 2520 in all the Protein | 0.4981151 |
| Biological process | GO:0009725 | response to hormone stimulus | 17 of 486 in the list | 86 of 2520 in all the Protein | 0.4988235 |
| Biological process | GO:0009116 | nucleoside metabolic process | 28 of 486 in the list | 143 of 2520 in all the Protein | 0.4988918 |
| Biological process | GO:0051603 | proteolysis involved in cellular protein catabolic process | 11 of 486 in the list | 55 of 2520 in all the Protein | 0.5010684 |
| Biological process | GO:0009639 | response to red or far red light | 5 of 486 in the list | 24 of 2520 in all the Protein | 0.5051226 |
| Biological process | GO:0044550 | secondary metabolite biosynthetic process | 5 of 486 in the list | 24 of 2520 in all the Protein | 0.5051226 |
| Biological process | GO:0033993 | response to lipid | 10 of 486 in the list | 50 of 2520 in all the Protein | 0.5061974 |
| Biological process | GO:0006152 | purine nucleoside catabolic process | 15 of 486 in the list | 76 of 2520 in all the Protein | 0.5070529 |
| Biological process | GO:0006195 | purine nucleotide catabolic process | 15 of 486 in the list | 76 of 2520 in all the Protein | 0.5070529 |
| Biological process | GO:0009143 | nucleoside triphosphate catabolic process | 15 of 486 in the list | 76 of 2520 in all the Protein | 0.5070529 |
| Biological process | GO:0009146 | purine nucleoside triphosphate catabolic process | 15 of 486 in the list | 76 of 2520 in all the Protein | 0.5070529 |
| Biological process | GO:0009154 | purine ribonucleotide catabolic process | 15 of 486 in the list | 76 of 2520 in all the Protein | 0.5070529 |
| Biological process | GO:0009164 | nucleoside catabolic process | 15 of 486 in the list | 76 of 2520 in all the Protein | 0.5070529 |
| Biological process | GO:0009203 | ribonucleoside triphosphate catabolic process | 15 of 486 in the list | 76 of 2520 in all the Protein | 0.5070529 |
| Biological process | GO:0009207 | purine ribonucleoside triphosphate catabolic process | 15 of 486 in the list | 76 of 2520 in all the Protein | 0.5070529 |
| Biological process | GO:0009261 | ribonucleotide catabolic process | 15 of 486 in the list | 76 of 2520 in all the Protein | 0.5070529 |
| Biological process | GO:0042454 | ribonucleoside catabolic process | 15 of 486 in the list | 76 of 2520 in all the Protein | 0.5070529 |
| Biological process | GO:0046130 | purine ribonucleoside catabolic process | 15 of 486 in the list | 76 of 2520 in all the Protein | 0.5070529 |
| Biological process | GO:0072523 | purine-containing compound catabolic process | 15 of 486 in the list | 76 of 2520 in all the Protein | 0.5070529 |
| Biological process | GO:0008652 | cellular amino acid biosynthetic process | 19 of 486 in the list | 97 of 2520 in all the Protein | 0.5117114 |
| Biological process | GO:0006396 | RNA processing | 9 of 486 in the list | 45 of 2520 in all the Protein | 0.5118723 |
| Biological process | GO:0006631 | fatty acid metabolic process | 9 of 486 in the list | 45 of 2520 in all the Protein | 0.5118723 |
| Biological process | GO:0009067 | aspartate family amino acid biosynthetic process | 4 of 486 in the list | 19 of 2520 in all the Protein | 0.5137655 |
| Biological process | GO:0009893 | positive regulation of metabolic process | 4 of 486 in the list | 19 of 2520 in all the Protein | 0.5137655 |
| Biological process | GO:0016568 | chromatin modification | 4 of 486 in the list | 19 of 2520 in all the Protein | 0.5137655 |
| Biological process | GO:0031325 | positive regulation of cellular metabolic process | 4 of 486 in the list | 19 of 2520 in all the Protein | 0.5137655 |
| Biological process | GO:0034654 | nucleobase-containing compound biosynthetic process | 23 of 486 in the list | 118 of 2520 in all the Protein | 0.5156116 |
| Biological process | GO:0010035 | response to inorganic substance | 38 of 486 in the list | 196 of 2520 in all the Protein | 0.5160349 |
| Biological process | GO:0008152 | metabolic process | 393 of 486 in the list | 2037 of 2520 in all the Protein | 0.5211265 |
| Biological process | GO:0009991 | response to extracellular stimulus | 3 of 486 in the list | 14 of 2520 in all the Protein | 0.5251903 |
| Biological process | GO:0031668 | cellular response to extracellular stimulus | 3 of 486 in the list | 14 of 2520 in all the Protein | 0.5251903 |
| Biological process | GO:0043603 | cellular amide metabolic process | 3 of 486 in the list | 14 of 2520 in all the Protein | 0.5251903 |
| Biological process | GO:0071496 | cellular response to external stimulus | 3 of 486 in the list | 14 of 2520 in all the Protein | 0.5251903 |
| Biological process | GO:0006461 | protein complex assembly | 7 of 486 in the list | 35 of 2520 in all the Protein | 0.5255241 |
| Biological process | GO:0043648 | dicarboxylic acid metabolic process | 7 of 486 in the list | 35 of 2520 in all the Protein | 0.5255241 |
| Biological process | GO:0045333 | cellular respiration | 7 of 486 in the list | 35 of 2520 in all the Protein | 0.5255241 |
| Biological process | GO:0070271 | protein complex biogenesis | 7 of 486 in the list | 35 of 2520 in all the Protein | 0.5255241 |
| Biological process | GO:0071310 | cellular response to organic substance | 7 of 486 in the list | 35 of 2520 in all the Protein | 0.5255241 |
| Biological process | GO:0046483 | heterocycle metabolic process | 85 of 486 in the list | 441 of 2520 in all the Protein | 0.5256145 |
| Biological process | GO:0044257 | cellular protein catabolic process | 11 of 486 in the list | 56 of 2520 in all the Protein | 0.5274556 |
| Biological process | GO:0000003 | reproduction | 15 of 486 in the list | 77 of 2520 in all the Protein | 0.5296138 |
| Biological process | GO:0009166 | nucleotide catabolic process | 15 of 486 in the list | 77 of 2520 in all the Protein | 0.5296138 |
| Biological process | GO:0009653 | anatomical structure morphogenesis | 15 of 486 in the list | 77 of 2520 in all the Protein | 0.5296138 |
| Biological process | GO:1901292 | nucleoside phosphate catabolic process | 15 of 486 in the list | 77 of 2520 in all the Protein | 0.5296138 |
| Biological process | GO:0006200 | ATP catabolic process | 6 of 486 in the list | 30 of 2520 in all the Protein | 0.5340506 |
| Biological process | GO:0022900 | electron transport chain | 6 of 486 in the list | 30 of 2520 in all the Protein | 0.5340506 |
| Biological process | GO:0006184 | GTP catabolic process | 9 of 486 in the list | 46 of 2520 in all the Protein | 0.5408374 |
| Biological process | GO:1901069 | guanosine-containing compound catabolic process | 9 of 486 in the list | 46 of 2520 in all the Protein | 0.5408374 |
| Biological process | GO:0006448 | regulation of translational elongation | 2 of 486 in the list | 9 of 2520 in all the Protein | 0.5422714 |
| Biological process | GO:0006518 | peptide metabolic process | 2 of 486 in the list | 9 of 2520 in all the Protein | 0.5422714 |
| Biological process | GO:0006857 | oligopeptide transport | 2 of 486 in the list | 9 of 2520 in all the Protein | 0.5422714 |
| Biological process | GO:0009723 | response to ethylene stimulus | 2 of 486 in the list | 9 of 2520 in all the Protein | 0.5422714 |
| Biological process | GO:0009809 | lignin biosynthetic process | 2 of 486 in the list | 9 of 2520 in all the Protein | 0.5422714 |
| Biological process | GO:0046112 | nucleobase biosynthetic process | 2 of 486 in the list | 9 of 2520 in all the Protein | 0.5422714 |
| Biological process | GO:0046685 | response to arsenic-containing substance | 2 of 486 in the list | 9 of 2520 in all the Protein | 0.5422714 |
| Biological process | GO:0048285 | organelle fission | 2 of 486 in the list | 9 of 2520 in all the Protein | 0.5422714 |
| Biological process | GO:0071704 | organic substance metabolic process | 293 of 486 in the list | 1522 of 2520 in all the Protein | 0.5431405 |
| Biological process | GO:0044767 | single-organism developmental process | 25 of 486 in the list | 130 of 2520 in all the Protein | 0.5436236 |
| Biological process | GO:0019538 | protein metabolic process | 124 of 486 in the list | 646 of 2520 in all the Protein | 0.5476956 |
| Biological process | GO:1901576 | organic substance biosynthetic process | 123 of 486 in the list | 641 of 2520 in all the Protein | 0.5494403 |
| Biological process | GO:0046434 | organophosphate catabolic process | 15 of 486 in the list | 78 of 2520 in all the Protein | 0.5518721 |
| Biological process | GO:0000097 | sulfur amino acid biosynthetic process | 4 of 486 in the list | 20 of 2520 in all the Protein | 0.5574413 |
| Biological process | GO:1901068 | guanosine-containing compound metabolic process | 10 of 486 in the list | 52 of 2520 in all the Protein | 0.5608225 |
| Biological process | GO:0009144 | purine nucleoside triphosphate metabolic process | 21 of 486 in the list | 110 of 2520 in all the Protein | 0.5610972 |
| Biological process | GO:0009199 | ribonucleoside triphosphate metabolic process | 21 of 486 in the list | 110 of 2520 in all the Protein | 0.5610972 |
| Biological process | GO:0009205 | purine ribonucleoside triphosphate metabolic process | 21 of 486 in the list | 110 of 2520 in all the Protein | 0.5610972 |
| Biological process | GO:0009260 | ribonucleotide biosynthetic process | 13 of 486 in the list | 68 of 2520 in all the Protein | 0.564185 |
| Biological process | GO:0046390 | ribose phosphate biosynthetic process | 13 of 486 in the list | 68 of 2520 in all the Protein | 0.564185 |
| Biological process | GO:1901565 | organonitrogen compound catabolic process | 20 of 486 in the list | 105 of 2520 in all the Protein | 0.5660054 |
| Biological process | GO:1901566 | organonitrogen compound biosynthetic process | 40 of 486 in the list | 210 of 2520 in all the Protein | 0.5664878 |
| Biological process | GO:0043623 | cellular protein complex assembly | 6 of 486 in the list | 31 of 2520 in all the Protein | 0.5688604 |
| Biological process | GO:0006464 | cellular protein modification process | 35 of 486 in the list | 184 of 2520 in all the Protein | 0.5693579 |
| Biological process | GO:0036211 | protein modification process | 35 of 486 in the list | 184 of 2520 in all the Protein | 0.5693579 |
| Biological process | GO:0009853 | photorespiration | 3 of 486 in the list | 15 of 2520 in all the Protein | 0.575249 |
| Biological process | GO:0009141 | nucleoside triphosphate metabolic process | 21 of 486 in the list | 111 of 2520 in all the Protein | 0.5796275 |
| Biological process | GO:0009056 | catabolic process | 52 of 486 in the list | 274 of 2520 in all the Protein | 0.581324 |
| Biological process | GO:0007017 | microtubule-based process | 5 of 486 in the list | 26 of 2520 in all the Protein | 0.5820774 |
| Biological process | GO:0009755 | hormone-mediated signaling pathway | 5 of 486 in the list | 26 of 2520 in all the Protein | 0.5820774 |
| Biological process | GO:0032870 | cellular response to hormone stimulus | 5 of 486 in the list | 26 of 2520 in all the Protein | 0.5820774 |
| Biological process | GO:0071495 | cellular response to endogenous stimulus | 5 of 486 in the list | 26 of 2520 in all the Protein | 0.5820774 |
| Biological process | GO:0034220 | ion transmembrane transport | 7 of 486 in the list | 37 of 2520 in all the Protein | 0.5894786 |
| Biological process | GO:0019439 | aromatic compound catabolic process | 18 of 486 in the list | 96 of 2520 in all the Protein | 0.596303 |
| Biological process | GO:0009066 | aspartate family amino acid metabolic process | 4 of 486 in the list | 21 of 2520 in all the Protein | 0.5989178 |
| Biological process | GO:0009156 | ribonucleoside monophosphate biosynthetic process | 4 of 486 in the list | 21 of 2520 in all the Protein | 0.5989178 |
| Biological process | GO:0009161 | ribonucleoside monophosphate metabolic process | 4 of 486 in the list | 21 of 2520 in all the Protein | 0.5989178 |
| Biological process | GO:0009309 | amine biosynthetic process | 4 of 486 in the list | 21 of 2520 in all the Protein | 0.5989178 |
| Biological process | GO:0072521 | purine-containing compound metabolic process | 27 of 486 in the list | 144 of 2520 in all the Protein | 0.6017934 |
| Biological process | GO:1901607 | alpha-amino acid biosynthetic process | 14 of 486 in the list | 75 of 2520 in all the Protein | 0.6023533 |
| Biological process | GO:0003002 | regionalization | 2 of 486 in the list | 10 of 2520 in all the Protein | 0.6026944 |
| Biological process | GO:0006222 | UMP biosynthetic process | 2 of 486 in the list | 10 of 2520 in all the Protein | 0.6026944 |
| Biological process | GO:0009173 | pyrimidine ribonucleoside monophosphate metabolic process | 2 of 486 in the list | 10 of 2520 in all the Protein | 0.6026944 |
| Biological process | GO:0009174 | pyrimidine ribonucleoside monophosphate biosynthetic process | 2 of 486 in the list | 10 of 2520 in all the Protein | 0.6026944 |
| Biological process | GO:0009615 | response to virus | 2 of 486 in the list | 10 of 2520 in all the Protein | 0.6026944 |
| Biological process | GO:0009813 | flavonoid biosynthetic process | 2 of 486 in the list | 10 of 2520 in all the Protein | 0.6026944 |
| Biological process | GO:0009890 | negative regulation of biosynthetic process | 2 of 486 in the list | 10 of 2520 in all the Protein | 0.6026944 |
| Biological process | GO:0015833 | peptide transport | 2 of 486 in the list | 10 of 2520 in all the Protein | 0.6026944 |
| Biological process | GO:0031327 | negative regulation of cellular biosynthetic process | 2 of 486 in the list | 10 of 2520 in all the Protein | 0.6026944 |
| Biological process | GO:0046049 | UMP metabolic process | 2 of 486 in the list | 10 of 2520 in all the Protein | 0.6026944 |
| Biological process | GO:0051128 | regulation of cellular component organization | 2 of 486 in the list | 10 of 2520 in all the Protein | 0.6026944 |
| Biological process | GO:0007165 | signal transduction | 20 of 486 in the list | 107 of 2520 in all the Protein | 0.6034562 |
| Biological process | GO:0051188 | cofactor biosynthetic process | 8 of 486 in the list | 43 of 2520 in all the Protein | 0.6074015 |
| Biological process | GO:0009152 | purine ribonucleotide biosynthetic process | 10 of 486 in the list | 54 of 2520 in all the Protein | 0.6128711 |
| Biological process | GO:1901575 | organic substance catabolic process | 49 of 486 in the list | 261 of 2520 in all the Protein | 0.6147785 |
| Biological process | GO:0009826 | unidimensional cell growth | 5 of 486 in the list | 27 of 2520 in all the Protein | 0.61805 |
| Biological process | GO:0023052 | signaling | 20 of 486 in the list | 108 of 2520 in all the Protein | 0.6216718 |
| Biological process | GO:0044700 | single organism signaling | 20 of 486 in the list | 108 of 2520 in all the Protein | 0.6216718 |
| Biological process | GO:0006730 | one-carbon metabolic process | 3 of 486 in the list | 16 of 2520 in all the Protein | 0.6218721 |
| Biological process | GO:0032446 | protein modification by small protein conjugation | 3 of 486 in the list | 16 of 2520 in all the Protein | 0.6218721 |
| Biological process | GO:0042181 | ketone biosynthetic process | 3 of 486 in the list | 16 of 2520 in all the Protein | 0.6218721 |
| Biological process | GO:0007154 | cell communication | 23 of 486 in the list | 124 of 2520 in all the Protein | 0.6218984 |
| Biological process | GO:0009259 | ribonucleotide metabolic process | 26 of 486 in the list | 140 of 2520 in all the Protein | 0.6226183 |
| Biological process | GO:0019693 | ribose phosphate metabolic process | 26 of 486 in the list | 140 of 2520 in all the Protein | 0.6226183 |
| Biological process | GO:0042221 | response to chemical stimulus | 54 of 486 in the list | 288 of 2520 in all the Protein | 0.6227873 |
| Biological process | GO:0006164 | purine nucleotide biosynthetic process | 11 of 486 in the list | 60 of 2520 in all the Protein | 0.6275762 |
| Biological process | GO:1901361 | organic cyclic compound catabolic process | 18 of 486 in the list | 98 of 2520 in all the Protein | 0.634433 |
| Biological process | GO:0009070 | serine family amino acid biosynthetic process | 4 of 486 in the list | 22 of 2520 in all the Protein | 0.6379739 |
| Biological process | GO:0009123 | nucleoside monophosphate metabolic process | 4 of 486 in the list | 22 of 2520 in all the Protein | 0.6379739 |
| Biological process | GO:0009124 | nucleoside monophosphate biosynthetic process | 4 of 486 in the list | 22 of 2520 in all the Protein | 0.6379739 |
| Biological process | GO:0015985 | energy coupled proton transport, down electrochemical gradient | 4 of 486 in the list | 22 of 2520 in all the Protein | 0.6379739 |
| Biological process | GO:0015986 | ATP synthesis coupled proton transport | 4 of 486 in the list | 22 of 2520 in all the Protein | 0.6379739 |
| Biological process | GO:0043094 | cellular metabolic compound salvage | 4 of 486 in the list | 22 of 2520 in all the Protein | 0.6379739 |
| Biological process | GO:0051274 | beta-glucan biosynthetic process | 4 of 486 in the list | 22 of 2520 in all the Protein | 0.6379739 |
| Biological process | GO:0016051 | carbohydrate biosynthetic process | 14 of 486 in the list | 77 of 2520 in all the Protein | 0.6448192 |
| Biological process | GO:0072330 | monocarboxylic acid biosynthetic process | 7 of 486 in the list | 39 of 2520 in all the Protein | 0.6488401 |
| Biological process | GO:0046039 | GTP metabolic process | 9 of 486 in the list | 50 of 2520 in all the Protein | 0.6490425 |
| Biological process | GO:1901135 | carbohydrate derivative metabolic process | 39 of 486 in the list | 211 of 2520 in all the Protein | 0.6504036 |
| Biological process | GO:0071669 | plant-type cell wall organization or biogenesis | 5 of 486 in the list | 28 of 2520 in all the Protein | 0.6521311 |
| Biological process | GO:0048856 | anatomical structure development | 29 of 486 in the list | 158 of 2520 in all the Protein | 0.6534756 |
| Biological process | GO:0009150 | purine ribonucleotide metabolic process | 23 of 486 in the list | 126 of 2520 in all the Protein | 0.655005 |
| Biological process | GO:0009126 | purine nucleoside monophosphate metabolic process | 2 of 486 in the list | 11 of 2520 in all the Protein | 0.6568851 |
| Biological process | GO:0009127 | purine nucleoside monophosphate biosynthetic process | 2 of 486 in the list | 11 of 2520 in all the Protein | 0.6568851 |
| Biological process | GO:0009129 | pyrimidine nucleoside monophosphate metabolic process | 2 of 486 in the list | 11 of 2520 in all the Protein | 0.6568851 |
| Biological process | GO:0009130 | pyrimidine nucleoside monophosphate biosynthetic process | 2 of 486 in the list | 11 of 2520 in all the Protein | 0.6568851 |
| Biological process | GO:0009167 | purine ribonucleoside monophosphate metabolic process | 2 of 486 in the list | 11 of 2520 in all the Protein | 0.6568851 |
| Biological process | GO:0009168 | purine ribonucleoside monophosphate biosynthetic process | 2 of 486 in the list | 11 of 2520 in all the Protein | 0.6568851 |
| Biological process | GO:0009553 | embryo sac development | 2 of 486 in the list | 11 of 2520 in all the Protein | 0.6568851 |
| Biological process | GO:0009644 | response to high light intensity | 2 of 486 in the list | 11 of 2520 in all the Protein | 0.6568851 |
| Biological process | GO:0009658 | chloroplast organization | 2 of 486 in the list | 11 of 2520 in all the Protein | 0.6568851 |
| Biological process | GO:0009812 | flavonoid metabolic process | 2 of 486 in the list | 11 of 2520 in all the Protein | 0.6568851 |
| Biological process | GO:0010228 | vegetative to reproductive phase transition of meristem | 2 of 486 in the list | 11 of 2520 in all the Protein | 0.6568851 |
| Biological process | GO:0042777 | plasma membrane ATP synthesis coupled proton transport | 2 of 486 in the list | 11 of 2520 in all the Protein | 0.6568851 |
| Biological process | GO:0050832 | defense response to fungus | 2 of 486 in the list | 11 of 2520 in all the Protein | 0.6568851 |
| Biological process | GO:0048731 | system development | 20 of 486 in the list | 110 of 2520 in all the Protein | 0.6569297 |
| Biological process | GO:0071702 | organic substance transport | 34 of 486 in the list | 185 of 2520 in all the Protein | 0.658191 |
| Biological process | GO:1901362 | organic cyclic compound biosynthetic process | 37 of 486 in the list | 201 of 2520 in all the Protein | 0.6585522 |
| Biological process | GO:0009165 | nucleotide biosynthetic process | 17 of 486 in the list | 94 of 2520 in all the Protein | 0.6600925 |
| Biological process | GO:1901293 | nucleoside phosphate biosynthetic process | 17 of 486 in the list | 94 of 2520 in all the Protein | 0.6600925 |
| Biological process | GO:0071822 | protein complex subunit organization | 8 of 486 in the list | 45 of 2520 in all the Protein | 0.6618486 |
| Biological process | GO:0006351 | transcription, DNA-dependent | 3 of 486 in the list | 17 of 2520 in all the Protein | 0.6648777 |
| Biological process | GO:0016042 | lipid catabolic process | 3 of 486 in the list | 17 of 2520 in all the Protein | 0.6648777 |
| Biological process | GO:0032774 | RNA biosynthetic process | 3 of 486 in the list | 17 of 2520 in all the Protein | 0.6648777 |
| Biological process | GO:0042180 | cellular ketone metabolic process | 3 of 486 in the list | 17 of 2520 in all the Protein | 0.6648777 |
| Biological process | GO:0006644 | phospholipid metabolic process | 6 of 486 in the list | 34 of 2520 in all the Protein | 0.6648813 |
| Biological process | GO:0032787 | monocarboxylic acid metabolic process | 13 of 486 in the list | 73 of 2520 in all the Protein | 0.674282 |
| Biological process | GO:0060255 | regulation of macromolecule metabolic process | 13 of 486 in the list | 73 of 2520 in all the Protein | 0.674282 |
| Biological process | GO:0019362 | pyridine nucleotide metabolic process | 4 of 486 in the list | 23 of 2520 in all the Protein | 0.6744709 |
| Biological process | GO:0046496 | nicotinamide nucleotide metabolic process | 4 of 486 in the list | 23 of 2520 in all the Protein | 0.6744709 |
| Biological process | GO:0080167 | response to karrikin | 4 of 486 in the list | 23 of 2520 in all the Protein | 0.6744709 |
| Biological process | GO:0034655 | nucleobase-containing compound catabolic process | 15 of 486 in the list | 84 of 2520 in all the Protein | 0.6760265 |
| Biological process | GO:0018193 | peptidyl-amino acid modification | 7 of 486 in the list | 40 of 2520 in all the Protein | 0.6765427 |
| Biological process | GO:0048518 | positive regulation of biological process | 7 of 486 in the list | 40 of 2520 in all the Protein | 0.6765427 |
| Biological process | GO:0018130 | heterocycle biosynthetic process | 32 of 486 in the list | 176 of 2520 in all the Protein | 0.6809011 |
| Biological process | GO:0016053 | organic acid biosynthetic process | 25 of 486 in the list | 139 of 2520 in all the Protein | 0.6896735 |
| Biological process | GO:0046394 | carboxylic acid biosynthetic process | 25 of 486 in the list | 139 of 2520 in all the Protein | 0.6896735 |
| Biological process | GO:0005975 | carbohydrate metabolic process | 56 of 486 in the list | 305 of 2520 in all the Protein | 0.6932722 |
| Biological process | GO:0015672 | monovalent inorganic cation transport | 6 of 486 in the list | 35 of 2520 in all the Protein | 0.6937138 |
| Biological process | GO:0065008 | regulation of biological quality | 13 of 486 in the list | 74 of 2520 in all the Protein | 0.6943092 |
| Biological process | GO:0006163 | purine nucleotide metabolic process | 24 of 486 in the list | 134 of 2520 in all the Protein | 0.6956285 |
| Biological process | GO:0010468 | regulation of gene expression | 9 of 486 in the list | 52 of 2520 in all the Protein | 0.697375 |
| Biological process | GO:0006486 | protein glycosylation | 2 of 486 in the list | 12 of 2520 in all the Protein | 0.7049958 |
| Biological process | GO:0009100 | glycoprotein metabolic process | 2 of 486 in the list | 12 of 2520 in all the Protein | 0.7049958 |
| Biological process | GO:0009101 | glycoprotein biosynthetic process | 2 of 486 in the list | 12 of 2520 in all the Protein | 0.7049958 |
| Biological process | GO:0019252 | starch biosynthetic process | 2 of 486 in the list | 12 of 2520 in all the Protein | 0.7049958 |
| Biological process | GO:0043413 | macromolecule glycosylation | 2 of 486 in the list | 12 of 2520 in all the Protein | 0.7049958 |
| Biological process | GO:0071407 | cellular response to organic cyclic compound | 2 of 486 in the list | 12 of 2520 in all the Protein | 0.7049958 |
| Biological process | GO:0006066 | alcohol metabolic process | 4 of 486 in the list | 24 of 2520 in all the Protein | 0.7083405 |
| Biological process | GO:0009966 | regulation of signal transduction | 4 of 486 in the list | 24 of 2520 in all the Protein | 0.7083405 |
| Biological process | GO:0010646 | regulation of cell communication | 4 of 486 in the list | 24 of 2520 in all the Protein | 0.7083405 |
| Biological process | GO:0023051 | regulation of signaling | 4 of 486 in the list | 24 of 2520 in all the Protein | 0.7083405 |
| Biological process | GO:0051273 | beta-glucan metabolic process | 4 of 486 in the list | 24 of 2520 in all the Protein | 0.7083405 |
| Biological process | GO:1901615 | organic hydroxy compound metabolic process | 4 of 486 in the list | 24 of 2520 in all the Protein | 0.7083405 |
| Biological process | GO:0044271 | cellular nitrogen compound biosynthetic process | 31 of 486 in the list | 173 of 2520 in all the Protein | 0.7121734 |
| Biological process | GO:0006818 | hydrogen transport | 5 of 486 in the list | 30 of 2520 in all the Protein | 0.7142511 |
| Biological process | GO:0015992 | proton transport | 5 of 486 in the list | 30 of 2520 in all the Protein | 0.7142511 |
| Biological process | GO:1901701 | cellular response to oxygen-containing compound | 5 of 486 in the list | 30 of 2520 in all the Protein | 0.7142511 |
| Biological process | GO:0044265 | cellular macromolecule catabolic process | 11 of 486 in the list | 64 of 2520 in all the Protein | 0.7156919 |
| Biological process | GO:0009607 | response to biotic stimulus | 16 of 486 in the list | 92 of 2520 in all the Protein | 0.7213687 |
| Biological process | GO:0006979 | response to oxidative stress | 10 of 486 in the list | 59 of 2520 in all the Protein | 0.7278804 |
| Biological process | GO:0030163 | protein catabolic process | 11 of 486 in the list | 65 of 2520 in all the Protein | 0.7355095 |
| Biological process | GO:0006220 | pyrimidine nucleotide metabolic process | 4 of 486 in the list | 25 of 2520 in all the Protein | 0.7395738 |
| Biological process | GO:0006221 | pyrimidine nucleotide biosynthetic process | 4 of 486 in the list | 25 of 2520 in all the Protein | 0.7395738 |
| Biological process | GO:0009064 | glutamine family amino acid metabolic process | 4 of 486 in the list | 25 of 2520 in all the Protein | 0.7395738 |
| Biological process | GO:0048229 | gametophyte development | 4 of 486 in the list | 25 of 2520 in all the Protein | 0.7395738 |
| Biological process | GO:0006098 | pentose-phosphate shunt | 3 of 486 in the list | 19 of 2520 in all the Protein | 0.7399209 |
| Biological process | GO:0006740 | NADPH regeneration | 3 of 486 in the list | 19 of 2520 in all the Protein | 0.7399209 |
| Biological process | GO:0009063 | cellular amino acid catabolic process | 3 of 486 in the list | 19 of 2520 in all the Protein | 0.7399209 |
| Biological process | GO:0009218 | pyrimidine ribonucleotide metabolic process | 3 of 486 in the list | 19 of 2520 in all the Protein | 0.7399209 |
| Biological process | GO:0009220 | pyrimidine ribonucleotide biosynthetic process | 3 of 486 in the list | 19 of 2520 in all the Protein | 0.7399209 |
| Biological process | GO:0009892 | negative regulation of metabolic process | 3 of 486 in the list | 19 of 2520 in all the Protein | 0.7399209 |
| Biological process | GO:0090407 | organophosphate biosynthetic process | 21 of 486 in the list | 121 of 2520 in all the Protein | 0.7444838 |
| Biological process | GO:0009408 | response to heat | 6 of 486 in the list | 37 of 2520 in all the Protein | 0.7462819 |
| Biological process | GO:0005982 | starch metabolic process | 2 of 486 in the list | 13 of 2520 in all the Protein | 0.7473517 |
| Biological process | GO:0009084 | glutaminefamily aminoacid biosynthetic process | 2 of 486 in the list | 13 of 2520 in all the Protein | 0.7473517 |
| Biological process | GO:0009112 | nucleobase metabolic process | 2 of 486 in the list | 13 of 2520 in all the Protein | 0.7473517 |
| Biological process | GO:0009312 | oligosaccharide biosynthetic process | 2 of 486 in the list | 13 of 2520 in all the Protein | 0.7473517 |
| Biological process | GO:0009743 | response to carbohydrate stimulus | 2 of 486 in the list | 13 of 2520 in all the Protein | 0.7473517 |
| Biological process | GO:0009909 | regulation of flower development | 2 of 486 in the list | 13 of 2520 in all the Protein | 0.7473517 |
| Biological process | GO:0031324 | negative regulation of cellular metabolic process | 2 of 486 in the list | 13 of 2520 in all the Protein | 0.7473517 |
| Biological process | GO:0070085 | glycosylation | 2 of 486 in the list | 13 of 2520 in all the Protein | 0.7473517 |
| Biological process | GO:2000241 | regulation of reproductive process | 2 of 486 in the list | 13 of 2520 in all the Protein | 0.7473517 |
| Biological process | GO:0031323 | regulation of cellular metabolic process | 13 of 486 in the list | 77 of 2520 in all the Protein | 0.749833 |
| Biological process | GO:0006732 | coenzyme metabolic process | 10 of 486 in the list | 61 of 2520 in all the Protein | 0.7669755 |
| Biological process | GO:0044711 | single-organism biosynthetic process | 32 of 486 in the list | 183 of 2520 in all the Protein | 0.767311 |
| Biological process | GO:0006733 | oxidoreduction coenzyme metabolic process | 4 of 486 in the list | 26 of 2520 in all the Protein | 0.7682095 |
| Biological process | GO:0019684 | photosynthesis, light reaction | 4 of 486 in the list | 26 of 2520 in all the Protein | 0.7682095 |
| Biological process | GO:0072524 | pyridine-containing compound metabolic process | 4 of 486 in the list | 26 of 2520 in all the Protein | 0.7682095 |
| Biological process | GO:0006508 | proteolysis | 29 of 486 in the list | 167 of 2520 in all the Protein | 0.7717042 |
| Biological process | GO:0006793 | phosphorus metabolic process | 71 of 486 in the list | 394 of 2520 in all the Protein | 0.7759146 |
| Biological process | GO:2000112 | regulation of cellular macromolecule biosynthetic process | 8 of 486 in the list | 50 of 2520 in all the Protein | 0.7766682 |
| Biological process | GO:0044707 | single-multicellular organism process | 28 of 486 in the list | 162 of 2520 in all the Protein | 0.7772438 |
| Biological process | GO:0044248 | cellular catabolic process | 34 of 486 in the list | 195 of 2520 in all the Protein | 0.7792007 |
| Biological process | GO:0080090 | regulation of primary metabolic process | 13 of 486 in the list | 79 of 2520 in all the Protein | 0.782908 |
| Biological process | GO:0006213 | pyrimidine nucleoside metabolic process | 2 of 486 in the list | 14 of 2520 in all the Protein | 0.7843786 |
| Biological process | GO:0006779 | porphyrin-containing compound biosynthetic process | 2 of 486 in the list | 14 of 2520 in all the Protein | 0.7843786 |
| Biological process | GO:0009555 | pollen development | 2 of 486 in the list | 14 of 2520 in all the Protein | 0.7843786 |
| Biological process | GO:0009642 | response to light intensity | 2 of 486 in the list | 14 of 2520 in all the Protein | 0.7843786 |
| Biological process | GO:0033014 | tetrapyrrole biosynthetic process | 2 of 486 in the list | 14 of 2520 in all the Protein | 0.7843786 |
| Biological process | GO:0046131 | pyrimidine ribonucleoside metabolic process | 2 of 486 in the list | 14 of 2520 in all the Protein | 0.7843786 |
| Biological process | GO:0046132 | pyrimidine ribonucleoside biosynthetic process | 2 of 486 in the list | 14 of 2520 in all the Protein | 0.7843786 |
| Biological process | GO:0046134 | pyrimidine nucleoside biosynthetic process | 2 of 486 in the list | 14 of 2520 in all the Protein | 0.7843786 |
| Biological process | GO:0046165 | alcohol biosynthetic process | 2 of 486 in the list | 14 of 2520 in all the Protein | 0.7843786 |
| Biological process | GO:1901617 | organic hydroxy compound biosynthetic process | 2 of 486 in the list | 14 of 2520 in all the Protein | 0.7843786 |
| Biological process | GO:0006796 | phosphate-containing compound metabolic process | 70 of 486 in the list | 390 of 2520 in all the Protein | 0.7864259 |
| Biological process | GO:0032501 | multicellular organismal process | 28 of 486 in the list | 163 of 2520 in all the Protein | 0.7885728 |
| Biological process | GO:0032989 | cellular component morphogenesis | 6 of 486 in the list | 39 of 2520 in all the Protein | 0.7920278 |
| Biological process | GO:0046700 | heterocycle catabolic process | 15 of 486 in the list | 91 of 2520 in all the Protein | 0.7930759 |
| Biological process | GO:0016310 | phosphorylation | 29 of 486 in the list | 169 of 2520 in all the Protein | 0.7940207 |
| Biological process | GO:0009069 | serine family amino acid metabolic process | 4 of 486 in the list | 27 of 2520 in all the Protein | 0.7943242 |
| Biological process | GO:0072528 | pyrimidine-containing compound biosynthetic process | 4 of 486 in the list | 27 of 2520 in all the Protein | 0.7943242 |
| Biological process | GO:1901605 | alpha-amino acid metabolic process | 18 of 486 in the list | 108 of 2520 in all the Protein | 0.7945958 |
| Biological process | GO:0010556 | regulationof macromolecule biosyntheticprocess | 8 of 486 in the list | 51 of 2520 in all the Protein | 0.7958174 |
| Biological process | GO:0009617 | response to bacterium | 9 of 486 in the list | 57 of 2520 in all the Protein | 0.7987115 |
| Biological process | GO:0009889 | regulation of biosynthetic process | 9 of 486 in the list | 57 of 2520 in all the Protein | 0.7987115 |
| Biological process | GO:0031326 | regulation of cellular biosynthetic process | 9 of 486 in the list | 57 of 2520 in all the Protein | 0.7987115 |
| Biological process | GO:0016070 | RNA metabolic process | 19 of 486 in the list | 114 of 2520 in all the Protein | 0.7996731 |
| Biological process | GO:0006739 | NADP metabolic process | 3 of 486 in the list | 21 of 2520 in all the Protein | 0.8009908 |
| Biological process | GO:0008654 | phospholipid biosynthetic process | 3 of 486 in the list | 21 of 2520 in all the Protein | 0.8009908 |
| Biological process | GO:0051641 | cellular localization | 24 of 486 in the list | 142 of 2520 in all the Protein | 0.8010389 |
| Biological process | GO:0055086 | nucleobase-containing small molecule metabolic process | 35 of 486 in the list | 203 of 2520 in all the Protein | 0.8047309 |
| Biological process | GO:0044270 | cellular nitrogen compound catabolic process | 15 of 486 in the list | 92 of 2520 in all the Protein | 0.8070448 |
| Biological process | GO:0051649 | establishment of localization in cell | 23 of 486 in the list | 137 of 2520 in all the Protein | 0.8074402 |
| Biological process | GO:0051704 | multi-organism process | 18 of 486 in the list | 109 of 2520 in all the Protein | 0.8074795 |
| Biological process | GO:0032502 | developmental process | 32 of 486 in the list | 187 of 2520 in all the Protein | 0.8093478 |
| Biological process | GO:0033036 | macromolecule localization | 26 of 486 in the list | 154 of 2520 in all the Protein | 0.8109269 |
| Biological process | GO:0048583 | regulation of response to stimulus | 5 of 486 in the list | 34 of 2520 in all the Protein | 0.8138994 |
| Biological process | GO:0060560 | developmental growth involved in morphogenesis | 5 of 486 in the list | 34 of 2520 in all the Protein | 0.8138994 |
| Biological process | GO:0044283 | small molecule biosynthetic process | 29 of 486 in the list | 171 of 2520 in all the Protein | 0.8148286 |
| Biological process | GO:0035556 | intracellular signal transduction | 9 of 486 in the list | 58 of 2520 in all the Protein | 0.8155927 |
| Biological process | GO:0044255 | cellular lipid metabolic process | 17 of 486 in the list | 104 of 2520 in all the Protein | 0.8156083 |
| Biological process | GO:0007275 | multicellular organismal development | 27 of 486 in the list | 160 of 2520 in all the Protein | 0.8156453 |
| Biological process | GO:0016567 | protein ubiquitination | 2 of 486 in the list | 15 of 2520 in all the Protein | 0.8165524 |
| Biological process | GO:0019319 | hexose biosynthetic process | 2 of 486 in the list | 15 of 2520 in all the Protein | 0.8165524 |
| Biological process | GO:0050794 | regulation of cellular process | 42 of 486 in the list | 243 of 2520 in all the Protein | 0.8200198 |
| Biological process | GO:0046686 | response to cadmium ion | 24 of 486 in the list | 144 of 2520 in all the Protein | 0.8229584 |
| Biological process | GO:0009057 | macromolecule catabolic process | 13 of 486 in the list | 82 of 2520 in all the Protein | 0.8265687 |
| Biological process | GO:0006081 | cellular aldehyde metabolic process | 3 of 486 in the list | 22 of 2520 in all the Protein | 0.8267303 |
| Biological process | GO:0006753 | nucleoside phosphate metabolic process | 32 of 486 in the list | 189 of 2520 in all the Protein | 0.8282972 |
| Biological process | GO:0009117 | nucleotide metabolic process | 32 of 486 in the list | 189 of 2520 in all the Protein | 0.8282972 |
| Biological process | GO:0048522 | positive regulation of cellular process | 4 of 486 in the list | 29 of 2520 in all the Protein | 0.8394319 |
| Biological process | GO:0072527 | pyrimidine-containing compound metabolic process | 4 of 486 in the list | 29 of 2520 in all the Protein | 0.8394319 |
| Biological process | GO:0010043 | response to zinc ion | 2 of 486 in the list | 16 of 2520 in all the Protein | 0.8443639 |
| Biological process | GO:0010605 | negative regulation of macromolecule metabolic process | 2 of 486 in the list | 16 of 2520 in all the Protein | 0.8443639 |
| Biological process | GO:0048580 | regulation of post-embryonic development | 2 of 486 in the list | 16 of 2520 in all the Protein | 0.8443639 |
| Biological process | GO:0034660 | ncRNA metabolic process | 8 of 486 in the list | 54 of 2520 in all the Protein | 0.8458849 |
| Biological process | GO:0046148 | pigment biosynthetic process | 3 of 486 in the list | 23 of 2520 in all the Protein | 0.8495692 |
| Biological process | GO:0000902 | cell morphogenesis | 5 of 486 in the list | 36 of 2520 in all the Protein | 0.8521683 |
| Biological process | GO:0007264 | small GTPase mediated signal transduction | 5 of 486 in the list | 36 of 2520 in all the Protein | 0.8521683 |
| Biological process | GO:0048589 | developmental growth | 5 of 486 in the list | 36 of 2520 in all the Protein | 0.8521683 |
| Biological process | GO:0019637 | organophosphate metabolic process | 39 of 486 in the list | 232 of 2520 in all the Protein | 0.8629162 |
| Biological process | GO:0006563 | L-serine metabolic process | 2 of 486 in the list | 17 of 2520 in all the Protein | 0.8682953 |
| Biological process | GO:0006778 | porphyrin-containing compound metabolic process | 2 of 486 in the list | 17 of 2520 in all the Protein | 0.8682953 |
| Biological process | GO:0009620 | response to fungus | 2 of 486 in the list | 17 of 2520 in all the Protein | 0.8682953 |
| Biological process | GO:0032879 | regulation of localization | 2 of 486 in the list | 17 of 2520 in all the Protein | 0.8682953 |
| Biological process | GO:0033013 | tetrapyrrole metabolic process | 2 of 486 in the list | 17 of 2520 in all the Protein | 0.8682953 |
| Biological process | GO:0006355 | regulation of transcription, DNA-dependent | 5 of 486 in the list | 37 of 2520 in all the Protein | 0.8687131 |
| Biological process | GO:0000413 | protein peptidyl-prolyl isomerization | 3 of 486 in the list | 24 of 2520 in all the Protein | 0.8697447 |
| Biological process | GO:2001141 | regulation of RNA biosynthetic process | 5 of 486 in the list | 38 of 2520 in all the Protein | 0.8836698 |
| Biological process | GO:0051707 | response to other organism | 13 of 486 in the list | 87 of 2520 in all the Protein | 0.8842461 |
| Biological process | GO:0010038 | response to metal ion | 24 of 486 in the list | 151 of 2520 in all the Protein | 0.8860168 |
| Biological process | GO:0018208 | peptidyl-proline modification | 3 of 486 in the list | 25 of 2520 in all the Protein | 0.8874953 |
| Biological process | GO:0048523 | negative regulation of cellular process | 3 of 486 in the list | 25 of 2520 in all the Protein | 0.8874953 |
| Biological process | GO:0040007 | growth | 7 of 486 in the list | 51 of 2520 in all the Protein | 0.8880757 |
| Biological process | GO:1901606 | alpha-amino acid catabolic process | 2 of 486 in the list | 18 of 2520 in all the Protein | 0.8888057 |
| Biological process | GO:0019222 | regulation of metabolic process | 18 of 486 in the list | 117 of 2520 in all the Protein | 0.8902846 |
| Biological process | GO:0050790 | regulation of catalytic activity | 6 of 486 in the list | 45 of 2520 in all the Protein | 0.8917318 |
| Biological process | GO:0006629 | lipid metabolic process | 22 of 486 in the list | 141 of 2520 in all the Protein | 0.8968723 |
| Biological process | GO:0006520 | cellular amino acid metabolic process | 29 of 486 in the list | 181 of 2520 in all the Protein | 0.8969177 |
| Biological process | GO:0051186 | cofactor metabolic process | 11 of 486 in the list | 78 of 2520 in all the Protein | 0.911613 |
| Biological process | GO:1901564 | organonitrogen compound metabolic process | 69 of 486 in the list | 406 of 2520 in all the Protein | 0.9121894 |
| Biological process | GO:0008104 | protein localization | 21 of 486 in the list | 138 of 2520 in all the Protein | 0.9157295 |
| Biological process | GO:0006605 | protein targeting | 3 of 486 in the list | 27 of 2520 in all the Protein | 0.9166456 |
| Biological process | GO:0042440 | pigment metabolic process | 3 of 486 in the list | 27 of 2520 in all the Protein | 0.9166456 |
| Biological process | GO:0050793 | regulation of developmental process | 3 of 486 in the list | 27 of 2520 in all the Protein | 0.9166456 |
| Biological process | GO:0051252 | regulation of RNA metabolic process | 5 of 486 in the list | 41 of 2520 in all the Protein | 0.920094 |
| Biological process | GO:0046364 | monosaccharide biosynthetic process | 2 of 486 in the list | 20 of 2520 in all the Protein | 0.9212325 |
| Biological process | GO:0072594 | establishment of protein localization to organelle | 2 of 486 in the list | 20 of 2520 in all the Protein | 0.9212325 |
| Biological process | GO:0065009 | regulation of molecular function | 6 of 486 in the list | 48 of 2520 in all the Protein | 0.9240404 |
| Biological process | GO:0048513 | organ development | 8 of 486 in the list | 61 of 2520 in all the Protein | 0.9251608 |
| Biological process | GO:0050789 | regulation of biological process | 50 of 486 in the list | 305 of 2520 in all the Protein | 0.9274955 |
| Biological process | GO:0034613 | cellular protein localization | 14 of 486 in the list | 99 of 2520 in all the Protein | 0.9314638 |
| Biological process | GO:0046907 | intracellular transport | 18 of 486 in the list | 123 of 2520 in all the Protein | 0.931476 |
| Biological process | GO:0070727 | cellular macromolecule localization | 14 of 486 in the list | 100 of 2520 in all the Protein | 0.9373976 |
| Biological process | GO:0048519 | negative regulation of biological process | 4 of 486 in the list | 36 of 2520 in all the Protein | 0.9374322 |
| Biological process | GO:0016049 | cell growth | 5 of 486 in the list | 43 of 2520 in all the Protein | 0.9384016 |
| Biological process | GO:0016482 | cytoplasmic transport | 5 of 486 in the list | 43 of 2520 in all the Protein | 0.9384016 |
| Biological process | GO:0016054 | organic acid catabolic process | 3 of 486 in the list | 29 of 2520 in all the Protein | 0.9387551 |
| Biological process | GO:0044282 | small molecule catabolic process | 3 of 486 in the list | 29 of 2520 in all the Protein | 0.9387551 |
| Biological process | GO:0044712 | single-organism catabolic process | 3 of 486 in the list | 29 of 2520 in all the Protein | 0.9387551 |
| Biological process | GO:0046395 | carboxylic acid catabolic process | 3 of 486 in the list | 29 of 2520 in all the Protein | 0.9387551 |
| Biological process | GO:0065007 | biological regulation | 52 of 486 in the list | 320 of 2520 in all the Protein | 0.9414326 |
| Biological process | GO:2000026 | regulation of multicellular organismal development | 2 of 486 in the list | 22 of 2520 in all the Protein | 0.9446055 |
| Biological process | GO:0048869 | cellular developmental process | 8 of 486 in the list | 64 of 2520 in all the Protein | 0.9464635 |
| Biological process | GO:0006413 | translational initiation | 3 of 486 in the list | 30 of 2520 in all the Protein | 0.9476512 |
| Biological process | GO:0044710 | single-organism metabolic process | 159 of 486 in the list | 903 of 2520 in all the Protein | 0.9508921 |
| Biological process | GO:0051239 | regulation of multicellular organismal process | 2 of 486 in the list | 23 of 2520 in all the Protein | 0.9536566 |
| Biological process | GO:0006457 | protein folding | 10 of 486 in the list | 80 of 2520 in all the Protein | 0.9620407 |
| Biological process | GO:0015031 | protein transport | 19 of 486 in the list | 136 of 2520 in all the Protein | 0.9620916 |
| Biological process | GO:0045184 | establishment of protein localization | 19 of 486 in the list | 136 of 2520 in all the Protein | 0.9620916 |
| Biological process | GO:0019752 | carboxylic acid metabolic process | 42 of 486 in the list | 271 of 2520 in all the Protein | 0.9628809 |
| Biological process | GO:0042742 | defense response to bacterium | 4 of 486 in the list | 40 of 2520 in all the Protein | 0.965036 |
| Biological process | GO:0043436 | oxoacid metabolic process | 42 of 486 in the list | 272 of 2520 in all the Protein | 0.9652484 |
| Biological process | GO:0006082 | organic acid metabolic process | 42 of 486 in the list | 273 of 2520 in all the Protein | 0.9674855 |
| Biological process | GO:0033365 | protein localization to organelle | 2 of 486 in the list | 25 of 2520 in all the Protein | 0.9677014 |
| Biological process | GO:0015979 | photosynthesis | 5 of 486 in the list | 48 of 2520 in all the Protein | 0.9688253 |
| Biological process | GO:0019219 | regulation of nucleobase-containing compound metabolic process | 5 of 486 in the list | 49 of 2520 in all the Protein | 0.9729242 |
| Biological process | GO:0048193 | Golgi vesicle transport | 2 of 486 in the list | 26 of 2520 in all the Protein | 0.9730887 |
| Biological process | GO:0006886 | intracellular protein transport | 12 of 486 in the list | 97 of 2520 in all the Protein | 0.9757301 |
| Biological process | GO:0051171 | regulation of nitrogen compound metabolic process | 5 of 486 in the list | 52 of 2520 in all the Protein | 0.9824137 |
| Biological process | GO:0008610 | lipid biosynthetic process | 9 of 486 in the list | 82 of 2520 in all the Protein | 0.9861836 |
| Biological process | GO:0016192 | vesicle-mediated transport | 9 of 486 in the list | 83 of 2520 in all the Protein | 0.9878063 |
| Biological process | GO:0044281 | small molecule metabolic process | 81 of 486 in the list | 509 of 2520 in all the Protein | 0.9879366 |
| Biological process | GO:0006418 | tRNA aminoacylation for protein translation | 2 of 486 in the list | 31 of 2520 in all the Protein | 0.9893714 |
| Biological process | GO:0008299 | isoprenoid biosynthetic process | 2 of 486 in the list | 31 of 2520 in all the Protein | 0.9893714 |
| Biological process | GO:0043038 | amino acid activation | 2 of 486 in the list | 31 of 2520 in all the Protein | 0.9893714 |
| Biological process | GO:0043039 | tRNA aminoacylation | 2 of 486 in the list | 31 of 2520 in all the Protein | 0.9893714 |
| Biological process | GO:0006720 | isoprenoid metabolic process | 2 of 486 in the list | 32 of 2520 in all the Protein | 0.9911993 |
| Biological process | GO:0022622 | root system development | 2 of 486 in the list | 32 of 2520 in all the Protein | 0.9911993 |
| Biological process | GO:0048364 | root development | 2 of 486 in the list | 32 of 2520 in all the Protein | 0.9911993 |
| Biological process | GO:0030154 | cell differentiation | 3 of 486 in the list | 42 of 2520 in all the Protein | 0.9929111 |
| Biological process | GO:0006399 | tRNA metabolic process | 2 of 486 in the list | 35 of 2520 in all the Protein | 0.9950288 |

**Supplementary Table S2B.** Supplementary data of GO terms involved in twenty types.

| Types | Gene Ontology ID | Gene Ontology term | Numbers of GO terms in the list | Numbers of GO terms in all the protein | P value |
| --- | --- | --- | --- | --- | --- |
| calcium | GO:0006816 | calcium ion transport | 3 of 486 in the list | 7 of 2520 in all the Protein | 0.135689 |
| calcium | GO:0005509 | calcium ion binding | 10 of 535 in the list | 59 of 2767 in all the Protein | 0.730784 |
| stress | GO:0009651 | response to salt stress | 24 of 486 in the list | 89 of 2520 in all the Protein | 0.045506 |
| stress | GO:0006950 | response to stress | 74 of 486 in the list | 332 of 2520 in all the Protein | 0.080202 |
| stress | GO:0006970 | response to osmotic stress | 27 of 486 in the list | 95 of 2520 in all the Protein | 0.018183 |
| stress | GO:0034976 | response to endoplasmic reticulum stress | 3 of 486 in the list | 10 of 2520 in all the Protein | 0.30058 |
| stress | GO:0006979 | response to oxidative stress | 10 of 486 in the list | 59 of 2520 in all the Protein | 0.72788 |
| stress | GO:0033554 | cellular response to stress | 15 of 486 in the list | 51 of 2520 in all the Protein | 0.052288 |
| stress | GO:0034599 | cellular response to oxidative stress | 3 of 486 in the list | 9 of 2520 in all the Protein | 0.242869 |
| stimulus | GO:0009628 | response to abiotic stimulus | 54 of 486 in the list | 234 of 2520 in all the Protein | 0.074673 |
| stimulus | GO:0009415 | response to water stimulus | 11 of 486 in the list | 39 of 2520 in all the Protein | 0.114208 |
| stimulus | GO:0051716 | cellular response to stimulus | 36 of 486 in the list | 158 of 2520 in all the Protein | 0.147733 |
| stimulus | GO:0070887 | cellular response to chemical stimulus | 12 of 486 in the list | 47 of 2520 in all the Protein | 0.179677 |
| stimulus | GO:0009733 | response to auxin stimulus | 6 of 486 in the list | 22 of 2520 in all the Protein | 0.238368 |
| stimulus | GO:0071365 | cellular response to auxin stimulus | 3 of 486 in the list | 9 of 2520 in all the Protein | 0.242869 |
| stimulus | GO:0009751 | response to salicylic acid stimulus | 2 of 486 in the list | 5 of 2520 in all the Protein | 0.248068 |
| stimulus | GO:0050896 | response to stimulus | 116 of 486 in the list | 574 of 2520 in all the Protein | 0.280193 |
| stimulus | GO:0009266 | response to temperature stimulus | 19 of 486 in the list | 90 of 2520 in all the Protein | 0.369158 |
| stimulus | GO:0009744 | response to sucrose stimulus | 2 of 486 in the list | 7 of 2520 in all the Protein | 0.403627 |
| stimulus | GO:0034285 | response to disaccharide stimulus | 2 of 486 in the list | 7 of 2520 in all the Protein | 0.403627 |
| stimulus | GO:0006974 | response to DNA damage stimulus | 4 of 486 in the list | 17 of 2520 in all the Protein | 0.421179 |
| stimulus | GO:0009737 | response to abscisic acid stimulus | 9 of 486 in the list | 42 of 2520 in all the Protein | 0.422335 |
| stimulus | GO:0009719 | response to endogenous stimulus | 18 of 486 in the list | 88 of 2520 in all the Protein | 0.432114 |
| stimulus | GO:0009416 | response to light stimulus | 13 of 486 in the list | 63 of 2520 in all the Protein | 0.442617 |
| stimulus | GO:0009605 | response to external stimulus | 7 of 486 in the list | 33 of 2520 in all the Protein | 0.458163 |
| stimulus | GO:0009725 | response to hormone stimulus | 17 of 486 in the list | 86 of 2520 in all the Protein | 0.498824 |
| stimulus | GO:0009991 | response to extracellular stimulus | 3 of 486 in the list | 14 of 2520 in all the Protein | 0.52519 |
| stimulus | GO:0031668 | cellular response to extracellular stimulus | 3 of 486 in the list | 14 of 2520 in all the Protein | 0.52519 |
| stimulus | GO:0071496 | cellular response to external stimulus | 3 of 486 in the list | 14 of 2520 in all the Protein | 0.52519 |
| stimulus | GO:0009723 | response to ethylene stimulus | 2 of 486 in the list | 9 of 2520 in all the Protein | 0.542271 |
| stimulus | GO:0032870 | cellular response to hormone stimulus | 5 of 486 in the list | 26 of 2520 in all the Protein | 0.582077 |
| stimulus | GO:0071495 | cellular response to endogenous stimulus | 5 of 486 in the list | 26 of 2520 in all the Protein | 0.582077 |
| stimulus | GO:0042221 | response to chemical stimulus | 54 of 486 in the list | 288 of 2520 in all the Protein | 0.622787 |
| stimulus | GO:0009607 | response to biotic stimulus | 16 of 486 in the list | 92 of 2520 in all the Protein | 0.721369 |
| stimulus | GO:0009743 | response to carbohydrate stimulus | 2 of 486 in the list | 13 of 2520 in all the Protein | 0.747352 |
| stimulus | GO:0048583 | regulation of response to stimulus | 5 of 486 in the list | 34 of 2520 in all the Protein | 0.813899 |
| signal transduction | GO:0007165 | signal transduction | 20 of 486 in the list | 107 of 2520 in all the Protein | 0.603456 |
| signal transduction | GO:0009966 | regulation of signal transduction | 4 of 486 in the list | 24 of 2520 in all the Protein | 0.708341 |
| signal transduction | GO:0035556 | intracellular signal transduction | 9 of 486 in the list | 58 of 2520 in all the Protein | 0.815593 |
| signal transduction | GO:0007264 | small GTPase mediated signal transduction | 5 of 486 in the list | 36 of 2520 in all the Protein | 0.852168 |
| polymerization | GO:0051258 | protein polymerization | 4 of 486 in the list | 12 of 2520 in all the Protein | 0.1863501 |
| transport | GO:0000275 | mitochondrial proton-transporting ATP synthase complex, catalytic core F(1) | 2 of 377 in the list | 2 of 1895 in all the Protein | 0.03949481 |
| transport | GO:0005753 | mitochondrial proton-transporting ATP synthase complex | 3 of 377 in the list | 7 of 1895 in all the Protein | 0.1458561 |
| transport | GO:0045261 | proton-transporting ATP synthase complex, catalytic core F(1) | 2 of 377 in the list | 8 of 1895 in all the Protein | 0.4938863 |
| transport | GO:0033178 | proton-transporting two-sector ATPase complex, catalytic domain | 3 of 377 in the list | 15 of 1895 in all the Protein | 0.5987913 |
| transport | GO:0045259 | proton-transporting ATP synthase complex | 3 of 377 in the list | 15 of 1895 in all the Protein | 0.5987913 |
| transport | GO:0016469 | proton-transporting two-sector ATPase complex | 5 of 377 in the list | 26 of 1895 in all the Protein | 0.6127877 |
| transport | GO:0033177 | proton-transporting two-sector ATPase complex, proton-transporting domain | 2 of 377 in the list | 11 of 1895 in all the Protein | 0.675529 |
| transport | GO:0015291 | secondary active transmembrane transporter activity | 7 of 535 in the list | 14 of 2767 in all the Protein | 0.009437382 |
| transport | GO:0022804 | active transmembrane transporter activity | 19 of 535 in the list | 69 of 2767 in all the Protein | 0.0599296 |
| transport | GO:0005342 | organic acid transmembrane transporter activity | 2 of 535 in the list | 3 of 2767 in all the Protein | 0.09759258 |
| transport | GO:0008514 | organic anion transmembrane transporter activity | 4 of 535 in the list | 10 of 2767 in all the Protein | 0.1090723 |
| transport | GO:0008509 | anion transmembrane transporter activity | 8 of 535 in the list | 27 of 2767 in all the Protein | 0.1335146 |
| transport | GO:0015416 | organic phosphonate transmembrane-transporting ATPase activity | 2 of 535 in the list | 4 of 2767 in all the Protein | 0.1705577 |
| transport | GO:0015604 | organic phosphonate transmembrane transporter activity | 2 of 535 in the list | 4 of 2767 in all the Protein | 0.1705577 |
| transport | GO:0015605 | organophosphate ester transmembrane transporter activity | 2 of 535 in the list | 4 of 2767 in all the Protein | 0.1705577 |
| transport | GO:0015075 | ion transmembrane transporter activity | 20 of 535 in the list | 85 of 2767 in all the Protein | 0.1941559 |
| transport | GO:0022857 | transmembrane transporter activity | 27 of 535 in the list | 123 of 2767 in all the Protein | 0.2586051 |
| transport | GO:0046961 | proton-transporting ATPase activity, rotational mechanism | 4 of 535 in the list | 15 of 2767 in all the Protein | 0.3268099 |
| transport | GO:0015144 | carbohydrate transmembrane transporter activity | 2 of 535 in the list | 6 of 2767 in all the Protein | 0.3282864 |
| transport | GO:0016703 | oxidoreductase activity, acting on single donors with incorporation of molecular oxygen, incorporation of one atom of oxygen (internal monooxygenases or internal mixed function oxidases) | 2 of 535 in the list | 6 of 2767 in all the Protein | 0.3282864 |
| transport | GO:0051119 | sugar transmembrane transporter activity | 2 of 535 in the list | 6 of 2767 in all the Protein | 0.3282864 |
| transport | GO:1901476 | carbohydrate transporter activity | 2 of 535 in the list | 6 of 2767 in all the Protein | 0.3282864 |
| transport | GO:0022891 | substrate-specific transmembrane transporter activity | 22 of 535 in the list | 103 of 2767 in all the Protein | 0.3361816 |
| transport | GO:0005215 | transporter activity | 38 of 535 in the list | 186 of 2767 in all the Protein | 0.3779595 |
| transport | GO:0015399 | primary active transmembrane transporter activity | 12 of 535 in the list | 56 of 2767 in all the Protein | 0.3968644 |
| transport | GO:0015405 | P-P-bond-hydrolysis-driven transmembrane transporter activity | 12 of 535 in the list | 56 of 2767 in all the Protein | 0.3968644 |
| transport | GO:0015238 | drug transmembrane transporter activity | 2 of 535 in the list | 7 of 2767 in all the Protein | 0.404988 |
| transport | GO:0090484 | drug transporter activity | 2 of 535 in the list | 7 of 2767 in all the Protein | 0.404988 |
| transport | GO:0015078 | hydrogen ion transmembrane transporter activity | 7 of 535 in the list | 32 of 2767 in all the Protein | 0.4265323 |
| transport | GO:0015077 | monovalent inorganic cation transmembrane transporter activity | 7 of 535 in the list | 34 of 2767 in all the Protein | 0.4950914 |
| transport | GO:0019829 | cation-transporting ATPase activity | 6 of 535 in the list | 29 of 2767 in all the Protein | 0.5008024 |
| transport | GO:0046933 | proton-transporting ATP synthase activity, rotational mechanism | 3 of 535 in the list | 14 of 2767 in all the Protein | 0.5270548 |
| transport | GO:0008324 | cation transmembrane transporter activity | 11 of 535 in the list | 56 of 2767 in all the Protein | 0.5311396 |
| transport | GO:0043225 | anion transmembrane-transporting ATPase activity | 2 of 535 in the list | 9 of 2767 in all the Protein | 0.5437699 |
| transport | GO:0022890 | inorganic cation transmembrane transporter activity | 9 of 535 in the list | 46 of 2767 in all the Protein | 0.5441505 |
| transport | GO:0022892 | substrate-specific transporter activity | 25 of 535 in the list | 131 of 2767 in all the Protein | 0.5664402 |
| transport | GO:0008565 | protein transporter activity | 3 of 535 in the list | 29 of 2767 in all the Protein | 0.9394165 |
| transport | GO:0006820 | anion transport | 7 of 486 in the list | 17 of 2520 in all the Protein | 0.03091516 |
| transport | GO:0050657 | nucleic acid transport | 2 of 486 in the list | 2 of 2520 in all the Protein | 0.03713208 |
| transport | GO:0050658 | RNA transport | 2 of 486 in the list | 2 of 2520 in all the Protein | 0.03713208 |
| transport | GO:0080170 | hydrogen peroxide transmembrane transport | 2 of 486 in the list | 2 of 2520 in all the Protein | 0.03713208 |
| transport | GO:0044765 | single-organism transport | 54 of 486 in the list | 226 of 2520 in all the Protein | 0.04228507 |
| transport | GO:0071705 | nitrogen compound transport | 10 of 486 in the list | 31 of 2520 in all the Protein | 0.05946341 |
| transport | GO:0006811 | ion transport | 21 of 486 in the list | 79 of 2520 in all the Protein | 0.06744802 |
| transport | GO:0015931 | nucleobase-containing compound transport | 4 of 486 in the list | 9 of 2520 in all the Protein | 0.07614114 |
| transport | GO:0006826 | iron ion transport | 3 of 486 in the list | 6 of 2520 in all the Protein | 0.09005839 |
| transport | GO:0006816 | calcium ion transport | 3 of 486 in the list | 7 of 2520 in all the Protein | 0.1356889 |
| transport | GO:0015849 | organic acid transport | 3 of 486 in the list | 7 of 2520 in all the Protein | 0.1356889 |
| transport | GO:0046942 | carboxylic acid transport | 3 of 486 in the list | 7 of 2520 in all the Protein | 0.1356889 |
| transport | GO:0008643 | carbohydrate transport | 4 of 486 in the list | 11 of 2520 in all the Protein | 0.1452293 |
| transport | GO:0015748 | organophosphate ester transport | 3 of 486 in the list | 8 of 2520 in all the Protein | 0.1872933 |
| transport | GO:0055085 | transmembrane transport | 23 of 486 in the list | 101 of 2520 in all the Protein | 0.2155162 |
| transport | GO:0006833 | water transport | 5 of 486 in the list | 17 of 2520 in all the Protein | 0.2173337 |
| transport | GO:0042044 | fluid transport | 5 of 486 in the list | 17 of 2520 in all the Protein | 0.2173337 |
| transport | GO:0015711 | organic anion transport | 3 of 486 in the list | 9 of 2520 in all the Protein | 0.2428693 |
| transport | GO:0070838 | divalent metal ion transport | 3 of 486 in the list | 9 of 2520 in all the Protein | 0.2428693 |
| transport | GO:0072511 | divalent inorganic cation transport | 3 of 486 in the list | 9 of 2520 in all the Protein | 0.2428693 |
| transport | GO:0006812 | cation transport | 16 of 486 in the list | 69 of 2520 in all the Protein | 0.2436822 |
| transport | GO:0015865 | purine nucleotide transport | 2 of 486 in the list | 5 of 2520 in all the Protein | 0.2480681 |
| transport | GO:0000041 | transition metal ion transport | 3 of 486 in the list | 10 of 2520 in all the Protein | 0.3005796 |
| transport | GO:0006810 | transport | 74 of 486 in the list | 363 of 2520 in all the Protein | 0.3050312 |
| transport | GO:0030001 | metal ion transport | 7 of 486 in the list | 29 of 2520 in all the Protein | 0.3200637 |
| transport | GO:0006839 | mitochondrial transport | 2 of 486 in the list | 6 of 2520 in all the Protein | 0.3270729 |
| transport | GO:0006862 | nucleotide transport | 2 of 486 in the list | 6 of 2520 in all the Protein | 0.3270729 |
| transport | GO:0015698 | inorganic anion transport | 2 of 486 in the list | 6 of 2520 in all the Protein | 0.3270729 |
| transport | GO:0006869 | lipid transport | 3 of 486 in the list | 11 of 2520 in all the Protein | 0.358836 |
| transport | GO:0006913 | nucleocytoplasmic transport | 3 of 486 in the list | 12 of 2520 in all the Protein | 0.4163313 |
| transport | GO:0051169 | nuclear transport | 3 of 486 in the list | 12 of 2520 in all the Protein | 0.4163313 |
| transport | GO:0042886 | amide transport | 3 of 486 in the list | 13 of 2520 in all the Protein | 0.4720385 |
| transport | GO:0022900 | electron transport chain | 6 of 486 in the list | 30 of 2520 in all the Protein | 0.5340506 |
| transport | GO:0006857 | oligopeptide transport | 2 of 486 in the list | 9 of 2520 in all the Protein | 0.5422714 |
| transport | GO:0034220 | ion transmembrane transport | 7 of 486 in the list | 37 of 2520 in all the Protein | 0.5894786 |
| transport | GO:0015833 | peptide transport | 2 of 486 in the list | 10 of 2520 in all the Protein | 0.6026944 |
| transport | GO:0015985 | energy coupled proton transport, down electrochemical gradient | 4 of 486 in the list | 22 of 2520 in all the Protein | 0.6379739 |
| transport | GO:0015986 | ATP synthesis coupled proton transport | 4 of 486 in the list | 22 of 2520 in all the Protein | 0.6379739 |
| transport | GO:0042777 | plasma membrane ATP synthesis coupled proton transport | 2 of 486 in the list | 11 of 2520 in all the Protein | 0.6568851 |
| transport | GO:0071702 | organic substance transport | 34 of 486 in the list | 185 of 2520 in all the Protein | 0.658191 |
| transport | GO:0015672 | monovalent inorganic cation transport | 6 of 486 in the list | 35 of 2520 in all the Protein | 0.6937138 |
| transport | GO:0006818 | hydrogen transport | 5 of 486 in the list | 30 of 2520 in all the Protein | 0.7142511 |
| transport | GO:0015992 | proton transport | 5 of 486 in the list | 30 of 2520 in all the Protein | 0.7142511 |
| transport | GO:0046907 | intracellular transport | 18 of 486 in the list | 123 of 2520 in all the Protein | 0.931476 |
| transport | GO:0016482 | cytoplasmic transport | 5 of 486 in the list | 43 of 2520 in all the Protein | 0.9384016 |
| transport | GO:0015031 | protein transport | 19 of 486 in the list | 136 of 2520 in all the Protein | 0.9620916 |
| transport | GO:0048193 | Golgi vesicle transport | 2 of 486 in the list | 26 of 2520 in all the Protein | 0.9730887 |
| transport | GO:0006886 | intracellular protein transport | 12 of 486 in the list | 97 of 2520 in all the Protein | 0.9757301 |
| transport | GO:0016192 | vesicle-mediated transport | 9 of 486 in the list | 83 of 2520 in all the Protein | 0.9878063 |
| assembly | GO:0006334 | nucleosome assembly | 19 of 486 in the list | 38 of 2520 in all the Protein | 1.72E-05 |
| assembly | GO:0065004 | protein-DNA complex assembly | 19 of 486 in the list | 38 of 2520 in all the Protein | 1.72E-05 |
| assembly | GO:0031497 | chromatin assembly | 19 of 486 in the list | 39 of 2520 in all the Protein | 2.76E-05 |
| assembly | GO:0006333 | chromatin assembly or disassembly | 19 of 486 in the list | 40 of 2520 in all the Protein | 4.33E-05 |
| assembly | GO:0034622 | cellular macromolecular complex assembly | 27 of 486 in the list | 71 of 2520 in all the Protein | 0.000148839 |
| assembly | GO:0065003 | macromolecular complex assembly | 28 of 486 in the list | 75 of 2520 in all the Protein | 0.000163127 |
| assembly | GO:0022607 | cellular component assembly | 28 of 486 in the list | 87 of 2520 in all the Protein | 0.002486031 |
| assembly | GO:0022618 | ribonucleoprotein complex assembly | 2 of 486 in the list | 2 of 2520 in all the Protein | 0.03713208 |
| assembly | GO:0042255 | ribosome assembly | 2 of 486 in the list | 2 of 2520 in all the Protein | 0.03713208 |
| assembly | GO:0042256 | mature ribosome assembly | 2 of 486 in the list | 2 of 2520 in all the Protein | 0.03713208 |
| assembly | GO:0070925 | organelle assembly | 2 of 486 in the list | 2 of 2520 in all the Protein | 0.03713208 |
| assembly | GO:0006461 | protein complex assembly | 7 of 486 in the list | 35 of 2520 in all the Protein | 0.5255241 |
| assembly | GO:0043623 | cellular protein complex assembly | 6 of 486 in the list | 31 of 2520 in all the Protein | 0.5688604 |
| peroxisome | GO:0005777 | peroxisome | 7 of 377 in the list | 44 of 1895 in all the Protein | 0.8027616 |
| peroxisome | GO:0006625 | protein targeting to peroxisome | 7 of 377 in the list | 44 of 1895 in all the Protein | 0.8027616 |
| peroxisome | GO:0007031 | peroxisome organization | 2 of 486 in the list | 8 of 2520 in all the Protein | 0.475738 |
| endoplasmic reticulum | GO:0044432 | endoplasmic reticulum part | 6 of 377 in the list | 15 of 1895 in all the Protein | 0.05895529 |
| endoplasmic reticulum | GO:0005789 | endoplasmic reticulum membrane | 5 of 377 in the list | 12 of 1895 in all the Protein | 0.07053212 |
| endoplasmic reticulum | GO:0042175 | nuclear outer membrane-endoplasmic reticulum membrane network | 5 of 377 in the list | 13 of 1895 in all the Protein | 0.09658718 |
| endoplasmic reticulum | GO:0031227 | intrinsic to endoplasmic reticulum membrane | 2 of 377 in the list | 4 of 1895 in all the Protein | 0.1790178 |
| endoplasmic reticulum | GO:0005783 | endoplasmic reticulum | 15 of 377 in the list | 93 of 1895 in all the Protein | 0.8578041 |
| endoplasmic reticulum | GO:0034976 | response to endoplasmic reticulum stress | 3 of 486 in the list | 10 of 2520 in all the Protein | 0.3005796 |
| Golgi | GO:0005794 | Golgi apparatus | 17 of 377 in the list | 89 of 1895 in all the Protein | 0.6199335 |
| Golgi | GO:0000139 | Golgi membrane | 2 of 377 in the list | 19 of 1895 in all the Protein | 0.9165626 |
| Golgi | GO:0044431 | Golgi apparatus part | 2 of 377 in the list | 29 of 1895 in all the Protein | 0.9872977 |
| Golgi | GO:0007030 | Golgi organization | 6 of 486 in the list | 11 of 2520 in all the Protein | 0.009559143 |
| Golgi | GO:0048193 | Golgi vesicle transport | 2 of 486 in the list | 26 of 2520 in all the Protein | 0.9730887 |
| plasma membrane | GO:0042777 | plasma membrane ATP synthesis coupled proton transport | 2 of 486 in the list | 11 of 2520 in all the Protein | 0.6568851 |
| plasma membrane | GO:0046658 | anchored to plasma membrane | 3 of 377 in the list | 12 of 1895 in all the Protein | 0.4380888 |
| plasma membrane | GO:0005886 | plasma membrane | 83 of 377 in the list | 420 of 1895 in all the Protein | 0.5551847 |
| plasma membrane | GO:0031226 | intrinsic to plasma membrane | 3 of 377 in the list | 16 of 1895 in all the Protein | 0.6452145 |
| plasma membrane | GO:0044459 | plasma membrane part | 5 of 377 in the list | 31 of 1895 in all the Protein | 0.769086 |
| cell wall | GO:0009827 | plant-type cell wall modification | 5 of 486 in the list | 16 of 2520 in all the Protein | 0.180175 |
| cell wall | GO:0005618 | cell wall | 32 of 377 in the list | 157 of 1895 in all the Protein | 0.4709325 |
| cell wall | GO:0042545 | cell wall modification | 5 of 486 in the list | 16 of 2520 in all the Protein | 0.180175 |
| cell wall | GO:0009664 | plant-type cell wall organization | 5 of 486 in the list | 17 of 2520 in all the Protein | 0.217334 |
| cell wall | GO:0009505 | plant-type cell wall | 10 of 377 in the list | 53 of 1895 in all the Protein | 0.6309556 |
| cell wall | GO:0044036 | cell wall macromolecule metabolic process | 4 of 486 in the list | 14 of 2520 in all the Protein | 0.277149 |
| cell wall | GO:0010383 | cell wall polysaccharide metabolic process | 2 of 486 in the list | 7 of 2520 in all the Protein | 0.403627 |
| cell wall | GO:0071555 | cell wall organization | 6 of 486 in the list | 25 of 2520 in all the Protein | 0.34815 |
| cell wall | GO:0071669 | plant-type cell wall organization or biogenesis | 5 of 486 in the list | 28 of 2520 in all the Protein | 0.652131 |
| cell wall | GO:0071554 | cell wall organization or biogenesis | 10 of 486 in the list | 47 of 2520 in all the Protein | 0.4213371 |
| cytoskeleton | GO:0015629 | actin cytoskeleton | 3 of 377 in the list | 9 of 1895 in all the Protein | 0.2587321 |
| cytoskeleton | GO:0015630 | microtubule cytoskeleton | 5 of 377 in the list | 28 of 1895 in all the Protein | 0.681901 |
| cytoskeleton | GO:0005856 | cytoskeleton | 7 of 377 in the list | 39 of 1895 in all the Protein | 0.684145 |
| cytoskeleton | GO:0030036 | actin cytoskeleton organization | 3 of 486 in the list | 12 of 2520 in all the Protein | 0.4163313 |
| cytoskeleton | GO:0007010 | cytoskeleton organization | 5 of 486 in the list | 26 of 2520 in all the Protein | 0.5820774 |
| amino acid | GO:0072341 | modified amino acid binding | 2 of 535 in the list | 3 of 2767 in all the Protein | 0.097593 |
| amino acid | GO:0016881 | acid-amino acid ligase activity | 8 of 535 in the list | 30 of 2767 in all the Protein | 0.209505 |
| amino acid | GO:0016597 | amino acid binding | 3 of 535 in the list | 9 of 2767 in all the Protein | 0.244171 |
| amino acid | GO:0070011 | peptidase activity, acting on L-amino acid peptides | 26 of 535 in the list | 136 of 2767 in all the Protein | 0.562372 |
| sucrose | GO:0009744 | response to sucrose stimulus | 2 of 486 in the list | 7 of 2520 in all the Protein | 0.403627 |
| sucrose | GO:0005985 | sucrose metabolic process | 2 of 486 in the list | 8 of 2520 in all the Protein | 0.475738 |
| glucose | GO:0006006 | glucose metabolic process | 19 of 486 in the list | 77 of 2520 in all the Protein | 0.1426899 |
| glucose | GO:0006007 | glucose catabolic process | 16 of 486 in the list | 69 of 2520 in all the Protein | 0.2436822 |
| sugar | GO:0005351 | sugar:hydrogen symporter activity | 2 of 535 in the list | 4 of 2767 in all the Protein | 0.1705577 |
| sugar | GO:0005402 | cation:sugar symporter activity | 2 of 535 in the list | 4 of 2767 in all the Protein | 0.1705577 |
| sugar | GO:0051119 | sugar transmembrane transporter activity | 2 of 535 in the list | 6 of 2767 in all the Protein | 0.3282864 |
| lignin | GO:0009808 | lignin metabolic process | 3 of 486 in the list | 13 of 2520 in all the Protein | 0.472039 |
| lignin | GO:0009809 | lignin biosynthetic process | 2 of 486 in the list | 9 of 2520 in all the Protein | 0.542271 |
| cellulose | GO:0030244 | cellulose biosynthetic process | 4 of 486 in the list | 14 of 2520 in all the Protein | 0.277149 |
| cellulose | GO:0030243 | cellulose metabolic process | 4 of 486 in the list | 16 of 2520 in all the Protein | 0.373216 |
| cellulose | GO:0016760 | cellulose synthase (UDP-forming) activity | 4 of 486 in the list | 14 of 2520 in all the Protein | 0.277149 |
| hemicellulose | GO:0010410 | hemicellulose metabolic process | 2 of 486 in the list | 6 of 2520 in all the Protein | 0.327073 |

**Supplementary Table S2C.** Supplementary data of 314 DEPs description.

| No. | Protein_ID | Description | Mass | Protein Coverage | Mean_Ratio |
| --- | --- | --- | --- | --- | --- |
| 1 | Unigene1242_All | 374 1849 PREDICTED: synaptotagmin-5 [Vitis vinifera] &gt;gi\|731421033\|ref\|XP_010661593.1\| PREDICTED: synaptotagmin-5 [Vitis vinifera] &gt;gi\|731421036\|ref\|XP_010661595.1\| PREDICTED: synaptotagmin-5 [Vitis vinifera] | 53718.86659 | 0.089 | 1.26 |
| 2 | Unigene30381_All | 147 1649 minus strand PREDICTED: pyruvate kinase, cytosolic isozyme [Cucumis sativus] &gt;gi\|700198858\|gb\|KGN54016.1\| hypothetical protein Csa_4G268000 [Cucumis sativus] | 54978.3925 | 0.2 | 1.22 |
| 3 | Unigene27190_All | 35 334 PREDICTED: pathogenesis-related protein PR-4-like isoform X1 [Populus euphratica] | 11086.75976 | 0.16 | 3.06 |
| 4 | Unigene7144_All | 43 420 Acyl Carrier Protein [Salvia miltiorrhiza] | 14049.12031 | 0.048 | 1.27 |
| 5 | CL10524.Contig1_All | 104 1711 minus strand PREDICTED: 4-coumarate--CoA ligase-like 7 [Prunus mume] | 59032.11349 | 0.039 | 1.26 |
| 6 | CL10341.Contig1_All | 156 1136 minus strand PREDICTED: PGR5-like protein 1B, chloroplastic [Vitis vinifera] &gt;gi\|731379717\|ref\|XP_010661470.1\| PREDICTED: PGR5-like protein 1B, chloroplastic [Vitis vinifera] | 37068.91099 | 0.107 | 1.24 |
| 7 | Unigene30220_All | 193 687 PREDICTED: uncharacterized protein LOC105632516 [Jatropha curcas] &gt;gi\|643732493\|gb\|KDP39589.1\| hypothetical protein JCGZ_02609 [Jatropha curcas] | 18750.77905 | 0.085 | 1.28 |
| 8 | Unigene10300_All | 187 996 pyrroline-5-carboxylate reductase [Jatropha curcas] &gt;gi\|643713364\|gb\|KDP26232.1\| hypothetical protein JCGZ_22478 [Jatropha curcas] &gt;gi\|814560404\|gb\|AKE33029.1\| pyrroline-5-carboxylate reductase [Jatropha curcas] | 28147.92431 | 0.041 | 1.23 |
| 9 | CL5181.Contig1_All | 57 935 PREDICTED: probable 3-hydroxyacyl-CoA dehydrogenase B0272.3 [Fragaria vesca subsp. vesca] | 31628.44366 | 0.191 | 1.24 |
| 10 | CL2078.Contig2_All | 61 408 minus strand hypothetical protein CICLE_v10013132mg [Citrus clementina] &gt;gi\|568854599\|ref\|XP_006480909.1\| PREDICTED: putative lipid-transfer protein DIR1 [Citrus sinensis] &gt;gi\|557531287\|gb\|ESR42470.1\| hypothetical protein CICLE_v10013132mg [Citrus clementina] | 12713.34137 | 0.198 | 1.24 |
| 11 | Unigene7832_All | 147 593 minus strand mago nashi [Taiwania cryptomerioides] | 17579.83448 | 0.04 | 1.46 |
| 12 | Unigene26703_All | 198 947 PREDICTED: reticulon-like protein B2 [Populus euphratica] | 28349.98893 | 0.284 | 1.41 |
| 13 | Unigene20115_All | 59 556 hypothetical protein PRUPE_ppa011540mg [Prunus persica] &gt;gi\|462412119\|gb\|EMJ17168.1\| hypothetical protein PRUPE_ppa011540mg [Prunus persica] | 18637.74455 | 0.482 | 1.25 |
| 14 | Unigene26598_All | 415 1434 minus strand PREDICTED: serine/threonine-protein kinase SAPK2 [Vitis vinifera] &gt;gi\|297741826\|emb\|CBI33139.3\| unnamed protein product [Vitis vinifera] | 38679.33115 | 0.047 | 1.29 |
| 15 | CL6183.Contig4_All | 29 976 minus strand PREDICTED: D-galacturonate reductase-like [Eucalyptus grandis] &gt;gi\|629121436\|gb\|KCW85926.1\| hypothetical protein EUGRSUZ_B02637 [Eucalyptus grandis] | 35177.38985 | 0.367 | 1.26 |
| 16 | Unigene13567_All | 277 1302 DnaJ homolog subfamily B member 11 [Morus notabilis] &gt;gi\|587870033\|gb\|EXB59329.1\| DnaJ homolog subfamily B member 11 [Morus notabilis] | 38892.75902 | 0.102 | 2.24 |
| 17 | CL8837.Contig1_All | 257 709 minus strand PREDICTED: universal stress protein A-like protein [Sesamum indicum] | 16646.71274 | 0.106 | 1.31 |
| 18 | Unigene283_All | 86 1645 minus strand PREDICTED: ATP-dependent 6-phosphofructokinase 4, chloroplastic isoform X1 [Vitis vinifera] &gt;gi\|297745021\|emb\|CBI38613.3\| unnamed protein product [Vitis vinifera] | 58265.54899 | 0.088 | 1.27 |
| 19 | CL12664.Contig1_All | 2 181 minus strand PREDICTED: histone H2A-like [Elaeis guineensis] | 6450.639055 | 0.233 | 1.46 |
| 20 | Unigene20369_All | 131 1057 PREDICTED: NADP-dependent D-sorbitol-6-phosphate dehydrogenase [Ricinus communis] &gt;gi\|223548017\|gb\|EEF49509.1\| aldo-keto reductase, putative [Ricinus communis] | 35207.03743 | 0.052 | 1.22 |
| 21 | Unigene528_All | 252 593 minus strand non-specific lipid transfer protein [Vitis vinifera] | 11782.77226 | 0.351 | 1.24 |
| 22 | Unigene29378_All | 175 1782 minus strand PREDICTED: calnexin homolog [Vitis vinifera] &gt;gi\|296085554\|emb\|CBI29286.3\| unnamed protein product [Vitis vinifera] | 61472.85679 | 0.095 | 1.39 |
| 23 | Unigene30455_All | 83 1063 PREDICTED: NADH-cytochrome b5 reductase-like protein [Vitis vinifera] &gt;gi\|297738925\|emb\|CBI28170.3\| unnamed protein product [Vitis vinifera] | 36139.61766 | 0.183 | 1.37 |
| 24 | Unigene30354_All | 130 1305 minus strand PREDICTED: ADP,ATP carrier protein, mitochondrial [Vitis vinifera] | 42756.96096 | 0.281 | 1.87 |
| 25 | Unigene16995_All | 163 1539 PREDICTED: hydroquinone glucosyltransferase [Jatropha curcas] &gt;gi\|643716885\|gb\|KDP28511.1\| hypothetical protein JCGZ_14282 [Jatropha curcas] | 50455.36189 | 0.078 | 1.25 |
| 26 | Unigene13628_All | 66 1031 minus strand PREDICTED: probable 6-phosphogluconolactonase 4, chloroplastic [Nicotiana tomentosiformis] | 35676.41928 | 0.214 | 1.48 |
| 27 | CL8022.Contig1_All | 343 2145 minus strand PREDICTED: CTP synthase-like [Jatropha curcas] &gt;gi\|643733503\|gb\|KDP40417.1\| hypothetical protein JCGZ_03767 [Jatropha curcas] | 66088.96892 | 0.015 | 1.4 |
| 28 | CL3837.Contig2_All | 206 1054 minus strand PREDICTED: aquaporin PIP2-1 [Gossypium raimondii] &gt;gi\|728831219\|gb\|KHG10662.1\| Aquaporin PIP2-1 -like protein [Gossypium arboreum] &gt;gi\|763769390\|gb\|KJB36605.1\| hypothetical protein B456_006G166800 [Gossypium raimondii] &gt;gi\|763769392\|gb\|KJB36607.1\| hypothetical protein B456_006G166800 [Gossypium raimondii] | 30413.61016 | 0.127 | 1.26 |
| 29 | CL12515.Contig3_All | 50 775 minus strand PREDICTED: probable mannitol dehydrogenase isoform X2 [Vitis vinifera] &gt;gi\|359497143\|ref\|XP_002266559.2\| PREDICTED: probable mannitol dehydrogenase isoform X1 [Vitis vinifera] | 26448.07123 | 0.211 | 1.21 |
| 30 | Unigene16830_All | 272 892 minus strand PREDICTED: ras-related protein Rab7 isoform X1 [Ricinus communis] &gt;gi\|223544944\|gb\|EEF46459.1\| protein with unknown function [Ricinus communis] | 23532.57294 | 0.275 | 1.35 |
| 31 | CL7291.Contig2_All | 236 1612 minus strand PREDICTED: histidinol dehydrogenase, chloroplastic isoform X1 [Vitis vinifera] | 50130.52913 | 0.046 | 1.35 |
| 32 | CL4498.Contig2_All | 2 505 minus strand PREDICTED: ubiquitin-conjugating enzyme E2-23 kDa [Vitis vinifera] &gt;gi\|731421598\|ref\|XP_010661801.1\| PREDICTED: ubiquitin-conjugating enzyme E2-23 kDa [Vitis vinifera] &gt;gi\|147789691\|emb\|CAN74060.1\| hypothetical protein VITISV_024680 [Vitis vinifera] &gt;gi\|297745317\|emb\|CBI40397.3\| unnamed protein product [Vitis vinifera] | 18991.87151 | 0.113 | 1.26 |
| 33 | CL7176.Contig2_All | 93 419 minus strand PREDICTED: 60S ribosomal protein L13-1-like [Populus euphratica] &gt;gi\|743829454\|ref\|XP_011023521.1\| PREDICTED: 60S ribosomal protein L13-1-like [Populus euphratica] &gt;gi\|743938210\|ref\|XP_011013522.1\| PREDICTED: 60S ribosomal protein L13-1-like [Populus euphratica] &gt;gi\|743938212\|ref\|XP_011013523.1\| PREDICTED: 60S ribosomal protein L13-1-like [Populus euphratica] | 12529.97538 | 0.505 | 1.36 |
| 34 | Unigene33388_All | 212 2833 PREDICTED: importin subunit beta-1 [Vitis vinifera] &gt;gi\|297735635\|emb\|CBI18129.3\| unnamed protein product [Vitis vinifera] | 97206.74716 | 0.024 | 1.28 |
| 35 | CL125.Contig1_All | 72 2459 PREDICTED: sucrose synthase [Vitis vinifera] &gt;gi\|731406211\|ref\|XP_010656083.1\| PREDICTED: sucrose synthase [Vitis vinifera] &gt;gi\|731406213\|ref\|XP_010656084.1\| PREDICTED: sucrose synthase [Vitis vinifera] &gt;gi\|297738510\|emb\|CBI27755.3\| unnamed protein product [Vitis vinifera] | 91596.99882 | 0.094 | 1.5 |
| 36 | Unigene29242_All | 56 493 minus strand unknown [Lotus japonicus] | 15604.79316 | 0.418 | 1.63 |
| 37 | Unigene13636_All | 27 962 minus strand unnamed protein product [Vitis vinifera] | 35552.38073 | 0.301 | 1.38 |
| 38 | CL12478.Contig5_All | 184 630 hypothetical protein EUTSA_v10019117mg [Eutrema salsugineum] &gt;gi\|557087266\|gb\|ESQ28118.1\| hypothetical protein EUTSA_v10019117mg [Eutrema salsugineum] | 16635.32344 | 0.49 | 1.44 |
| 39 | Unigene967_All | 269 499 minus strand PREDICTED: copper transport protein CCH-like isoform X2 [Nicotiana tomentosiformis] | 8305.276175 | 0.494 | 1.25 |
| 40 | CL384.Contig6_All | 104 1189 PREDICTED: probable mannitol dehydrogenase [Vitis vinifera] &gt;gi\|297741856\|emb\|CBI33216.3\| unnamed protein product [Vitis vinifera] | 39745.07101 | 0.511 | 1.45 |
| 41 | CL6175.Contig1_All | 169 528 PREDICTED: 60S ribosomal protein L34 [Eucalyptus grandis] &gt;gi\|629122355\|gb\|KCW86845.1\| hypothetical protein EUGRSUZ_B03442 [Eucalyptus grandis] | 13730.85169 | 0.383 | 1.42 |
| 42 | Unigene9708_All | 483 1841 minus strand PREDICTED: dual specificity protein kinase shkB [Vitis vinifera] &gt;gi\|297740576\|emb\|CBI30758.3\| unnamed protein product [Vitis vinifera] | 51863.76964 | 0.022 | 1.48 |
| 43 | Unigene23520_All | 378 1997 PREDICTED: 4-coumarate--CoA ligase-like 7 isoform X1 [Jatropha curcas] &gt;gi\|643737999\|gb\|KDP43987.1\| hypothetical protein JCGZ_05454 [Jatropha curcas] | 59031.02905 | 0.106 | 1.22 |
| 44 | Unigene6818_All | 167 619 PREDICTED: NADH dehydrogenase [ubiquinone] iron-sulfur protein 4, mitochondrial-like isoform X1 [Nelumbo nucifera] | 16930.63409 | 0.106 | 1.58 |
| 45 | Unigene10069_All | 54 554 minus strand hypothetical protein GLYMA_19G236500 [Glycine max] | 19099.59907 | 0.246 | 1.31 |
| 46 | Unigene26629_All | 160 1512 minus strand uncharacterized protein LOC100806482 [Glycine max] &gt;gi\|255636463\|gb\|ACU18570.1\| unknown [Glycine max] &gt;gi\|734406014\|gb\|KHN33783.1\| Glyceraldehyde-3-phosphate dehydrogenase B, chloroplastic [Glycine soja] &gt;gi\|947112592\|gb\|KRH60894.1\| hypothetical protein GLYMA_04G015900 [Glycine max] &gt;gi\|947112593\|gb\|KRH60895.1\| hypothetical protein GLYMA_04G015900 [Glycine max] &gt;gi\|947112594\|gb\|KRH60896.1\| hypothetical protein GLYMA_04G015900 [Glycine max] | 48494.02185 | 0.31 | 1.25 |
| 47 | Unigene23446_All | 73 717 PREDICTED: histone H1-like [Nelumbo nucifera] | 22645.57892 | 0.367 | 1.41 |
| 48 | Unigene7120_All | 225 1055 minus strand PREDICTED: NADH--cytochrome b5 reductase 1 [Vitis vinifera] &gt;gi\|296085882\|emb\|CBI31206.3\| unnamed protein product [Vitis vinifera] | 31066.8397 | 0.303 | 1.26 |
| 49 | CL12250.Contig2_All | 41 472 core histone H2A/H2B/H3/H4 [Medicago truncatula] &gt;gi\|657389225\|gb\|KEH30941.1\| core histone H2A/H2B/H3/H4 [Medicago truncatula] | 15748.6393 | 0.84 | 1.48 |
| 50 | Unigene39_All | 64 510 PREDICTED: probable histone H2B.1 [Malus domestica] &gt;gi\|658018994\|ref\|XP_008344843.1\| PREDICTED: probable histone H2B.1 [Malus domestica] &gt;gi\|658028562\|ref\|XP_008349713.1\| PREDICTED: probable histone H2B.1 [Malus domestica] &gt;gi\|694354102\|ref\|XP_009358338.1\| PREDICTED: probable histone H2B.1 [Pyrus x bretschneideri] &gt;gi\|694354144\|ref\|XP_009358349.1\| PREDICTED: probable histone H2B.1 [Pyrus x bretschneideri] &gt;gi\|694420970\|ref\|XP_009338362.1\| PREDICTED: probable histone H2B.1 [Pyrus x bretschneideri] | 16238.99504 | 0.846 | 1.63 |
| 51 | Unigene30372_All | 158 1246 PREDICTED: probable cysteine proteinase A494 [Jatropha curcas] &gt;gi\|643735102\|gb\|KDP41743.1\| hypothetical protein JCGZ_26761 [Jatropha curcas] | 40413.90516 | 0.135 | 1.43 |
| 52 | Unigene29230_All | 147 443 minus strand histone H2A [Zea mays] | 10786.85077 | 0.374 | 1.8 |
| 53 | Unigene13590_All | 156 1496 PREDICTED: NEDD8-activating enzyme E1 catalytic subunit [Vitis vinifera] &gt;gi\|297736997\|emb\|CBI26198.3\| unnamed protein product [Vitis vinifera] | 49989.45188 | 0.069 | 1.39 |
| 54 | CL8474.Contig1_All | 120 896 minus strand PREDICTED: 60S ribosomal protein L8 [Vitis vinifera] | 28245.90923 | 0.49 | 1.63 |
| 55 | Unigene23152_All | 728 1309 hypothetical protein CICLE_v10012248mg [Citrus clementina] &gt;gi\|568854922\|ref\|XP_006481065.1\| PREDICTED: mitochondrial succinate-fumarate transporter 1 [Citrus sinensis] &gt;gi\|557531489\|gb\|ESR42672.1\| hypothetical protein CICLE_v10012248mg [Citrus clementina] | 21626.20424 | 0.036 | 2.04 |
| 56 | CL11572.Contig2_All | 127 1590 PREDICTED: protein DETOXIFICATION 35 [Ricinus communis] &gt;gi\|223547574\|gb\|EEF49069.1\| multidrug resistance pump, putative [Ricinus communis] | 53370.18908 | 0.02 | 1.52 |
| 57 | Unigene13498_All | 98 1171 minus strand Papain family cysteine protease [Theobroma cacao] &gt;gi\|508723000\|gb\|EOY14897.1\| Papain family cysteine protease [Theobroma cacao] | 39653.46268 | 0.07 | 1.53 |
| 58 | Unigene32_All | 74 502 minus strand PREDICTED: histone H2B-like [Gossypium raimondii] &gt;gi\|763767995\|gb\|KJB35210.1\| hypothetical protein B456_006G104400 [Gossypium raimondii] | 15966.91936 | 0.692 | 1.25 |
| 59 | CL5154.Contig1_All | 7 324 minus strand PREDICTED: cinnamoyl-CoA reductase 1 isoform X2 [Vitis vinifera] | 12126.41104 | 0.123 | 1.68 |
| 60 | Unigene6634_All | 178 1326 PREDICTED: ADP,ATP carrier protein 3, mitochondrial [Prunus mume] | 41633.50546 | 0.287 | 1.4 |
| 61 | Unigene23477_All | 130 555 PREDICTED: histone H2B.3-like [Nelumbo nucifera] | 15691.7097 | 0.782 | 2.02 |
| 62 | CL5012.Contig1_All | 179 964 PREDICTED: uncharacterized protein At3g49720 [Vitis vinifera] &gt;gi\|731384077\|ref\|XP_010647997.1\| PREDICTED: uncharacterized protein At3g49720 [Vitis vinifera] &gt;gi\|297736364\|emb\|CBI25087.3\| unnamed protein product [Vitis vinifera] | 28977.18516 | 0.065 | 1.24 |
| 63 | CL9787.Contig2_All | 125 787 minus strand unknown [Picea sitchensis] &gt;gi\|224286250\|gb\|ACN40834.1\| unknown [Picea sitchensis] | 25473.62235 | 0.29 | 1.23 |
| 64 | CL2804.Contig2_All | 61 279 PREDICTED: ATP synthase subunit epsilon, mitochondrial [Vitis vinifera] | 8069.968862 | 0.356 | 1.36 |
| 65 | Unigene6703_All | 87 284 BnaAnng32380D [Brassica napus] | 7175.972138 | 0.136 | 1.34 |
| 66 | CL3417.Contig3_All | 186 1238 beta 1-3 glucanase [Vitis hybrid cultivar] | 38963.11976 | 0.071 | 1.24 |
| 67 | CL4702.Contig1_All | 193 1476 PREDICTED: pyruvate dehydrogenase E1 component subunit alpha-3, chloroplastic [Populus euphratica] | 47376.91838 | 0.028 | 1.33 |
| 68 | Unigene13285_All | 42 539 minus strand glutathione peroxidase [Ziziphus jujuba] | 18877.37132 | 0.09 | 1.21 |
| 69 | Unigene35030_All | 178 672 PREDICTED: V-type proton ATPase 16 kDa proteolipid subunit [Jatropha curcas] &gt;gi\|802708037\|ref\|XP_012084431.1\| PREDICTED: V-type proton ATPase 16 kDa proteolipid subunit [Jatropha curcas] &gt;gi\|643715691\|gb\|KDP27632.1\| hypothetical protein JCGZ_19637 [Jatropha curcas] &gt;gi\|643723204\|gb\|KDP32809.1\| hypothetical protein JCGZ_12101 [Jatropha curcas] | 16769.80486 | 0.109 | 1.36 |
| 70 | Unigene26615_All | 103 1272 PREDICTED: S-adenosylmethionine synthase 5 [Vitis vinifera] &gt;gi\|223635289\|sp\|A7Q0V4.1\|METK5_VITVI RecName: Full=S-adenosylmethionine synthase 5; Short=AdoMet synthase 5; AltName: Full=Methionine adenosyltransferase 5; Short=MAT 5 | 43319.79931 | 0.338 | 1.46 |
| 71 | CL8474.Contig2_All | 134 910 minus strand PREDICTED: 60S ribosomal protein L8 [Vitis vinifera] | 28263.86565 | 0.49 | 1.72 |
| 72 | Unigene797_All | 4 774 HAD superfamily, subfamily IIIB acid phosphatase [Theobroma cacao] &gt;gi\|508702449\|gb\|EOX94345.1\| HAD superfamily, subfamily IIIB acid phosphatase [Theobroma cacao] | 30015.42564 | 0.066 | 1.25 |
| 73 | Unigene30257_All | 111 566 minus strand unknown [Populus trichocarpa] | 16449.36407 | 0.099 | 1.32 |
| 74 | Unigene72967_All | 3 263 minus strand NADH dehydrogenase subunit 7 (mitochondrion) [Cannabis sativa] | 10063.16779 | 0.08 | 1.42 |
| 75 | Unigene16556_All | 41 481 serine/threonine kinase-like [Vitis vinifera] &gt;gi\|28629126\|gb\|AAO49473.1\| putative serine/threonine kinase [Vitis vinifera] &gt;gi\|296082851\|emb\|CBI22152.3\| unnamed protein product [Vitis vinifera] | 16876.51595 | 0.204 | 1.21 |
| 76 | Unigene23885_All | 223 666 minus strand plasma membrane intrinsic protein PIP2.2 [Populus trichocarpa x Populus deltoides] | 16143.21606 | 0.162 | 1.47 |
| 77 | Unigene13353_All | 50 535 unnamed protein product [Coffea canephora] | 18536.30463 | 0.438 | 1.52 |
| 78 | Unigene3894_All | 199 1554 minus strand PREDICTED: serine carboxypeptidase-like 27 [Populus euphratica] | 51180.4572 | 0.111 | 1.31 |
| 79 | Unigene10064_All | 79 531 minus strand unknown [Lotus japonicus] | 17074.36862 | 0.126 | 1.43 |
| 80 | Unigene30412_All | 494 2197 PREDICTED: calcium-dependent protein kinase 26 isoform X1 [Vitis vinifera] | 64283.84967 | 0.033 | 1.36 |
| 81 | CL1405.Contig2_All | 414 2474 minus strand PREDICTED: fimbrin-1 [Vitis vinifera] | 77174.96042 | 0.012 | 1.31 |
| 82 | CL10427.Contig2_All | 90 836 PREDICTED: L-ascorbate peroxidase, cytosolic [Eucalyptus grandis] &gt;gi\|702272326\|ref\|XP_010043679.1\| PREDICTED: L-ascorbate peroxidase, cytosolic [Eucalyptus grandis] &gt;gi\|629121183\|gb\|KCW85673.1\| hypothetical protein EUGRSUZ_B02456 [Eucalyptus grandis] &gt;gi\|629121184\|gb\|KCW85674.1\| hypothetical protein EUGRSUZ_B02456 [Eucalyptus grandis] | 27657.97773 | 0.43 | 1.21 |
| 83 | Unigene16912_All | 183 1655 minus strand vacuolar processing enzyme a [Populus tomentosa] | 54682.81009 | 0.029 | 1.28 |
| 84 | Unigene16784_All | 158 412 minus strand hypothetical protein OsJ_25365 [Oryza sativa Japonica Group] | 9678.171156 | 0.129 | 1.35 |
| 85 | Unigene9863_All | 307 741 PREDICTED: transmembrane and coiled-coil domain-containing protein 1 [Vitis vinifera] &gt;gi\|297739810\|emb\|CBI29992.3\| unnamed protein product [Vitis vinifera] | 16056.75104 | 0.069 | 1.56 |
| 86 | CL2313.Contig1_All | 121 1017 minus strand PREDICTED: histone H1-like [Pyrus x bretschneideri] | 30867.21268 | 0.401 | 1.22 |
| 87 | Unigene29869_All | 252 1637 PREDICTED: serine carboxypeptidase II-2 [Fragaria vesca subsp. vesca] | 51939.86656 | 0.108 | 1.22 |
| 88 | Unigene16554_All | 80 1441 minus strand PREDICTED: serine carboxypeptidase-like 7 [Ricinus communis] | 51270.00041 | 0.115 | 1.21 |
| 89 | Unigene26224_All | 111 1544 hypothetical protein POPTR_0009s08030g [Populus trichocarpa] &gt;gi\|222850378\|gb\|EEE87925.1\| hypothetical protein POPTR_0009s08030g [Populus trichocarpa] | 53043.88663 | 0.019 | 1.45 |
| 90 | Unigene21551_All | 121 561 PREDICTED: ubiquitin-conjugating enzyme E2 28-like [Sesamum indicum] &gt;gi\|747059322\|ref\|XP_011076035.1\| PREDICTED: ubiquitin-conjugating enzyme E2 28-like [Sesamum indicum] | 16649.40323 | 0.129 | 1.55 |
| 91 | Unigene23765_All | 221 826 Ras-related protein RABD2c [Morus notabilis] &gt;gi\|587914287\|gb\|EXC02066.1\| Ras-related protein RABD2c [Morus notabilis] | 22747.28615 | 0.322 | 1.28 |
| 92 | CL941.Contig3_All | 20 487 hypothetical protein Ccrd_026729 [Cynara cardunculus var. scolymus] &gt;gi\|976915786\|gb\|KVI01607.1\| Histone core [Cynara cardunculus var. scolymus] | 16758.33692 | 0.353 | 1.51 |
| 93 | Unigene30477_All | 179 826 minus strand PREDICTED: ras-related protein RABE1c-like [Nelumbo nucifera] | 24134.36712 | 0.338 | 1.61 |
| 94 | Unigene1358_All | 142 1005 minus strand PREDICTED: L-ascorbate peroxidase 3, peroxisomal [Ricinus communis] &gt;gi\|223529615\|gb\|EEF31563.1\| L-ascorbate peroxidase 1, cytosolic, putative [Ricinus communis] | 31996.54405 | 0.149 | 1.22 |
| 95 | CL6952.Contig3_All | 65 1108 PLC-like phosphodiesterases superfamily protein isoform 1 [Theobroma cacao] &gt;gi\|508699548\|gb\|EOX91444.1\| PLC-like phosphodiesterases superfamily protein isoform 1 [Theobroma cacao] | 39162.47729 | 0.092 | 1.28 |
| 96 | Unigene6616_All | 39 1130 minus strand predicted protein [Ostreococcus lucimarinus CCE9901] &gt;gi\|144581827\|gb\|ABO99882.1\| predicted protein [Ostreococcus lucimarinus CCE9901] | 39202.94481 | 0.071 | 1.87 |
| 97 | CL3599.Contig1_All | 327 2117 Auxin efflux facilitator isoform 1 [Theobroma cacao] &gt;gi\|508774091\|gb\|EOY21347.1\| Auxin efflux facilitator isoform 1 [Theobroma cacao] | 65224.27647 | 0.039 | 1.35 |
| 98 | CL2281.Contig2_All | 246 2516 minus strand PREDICTED: subtilisin-like protease [Vitis vinifera] | 81248.52811 | 0.229 | 1.29 |
| 99 | Unigene20594_All | 22 624 minus strand PREDICTED: abscisic acid receptor PYL4-like [Populus euphratica] &gt;gi\|743781773\|ref\|XP_011003478.1\| PREDICTED: abscisic acid receptor PYL4-like [Populus euphratica] | 22561.26329 | 0.03 | 2.93 |
| 100 | Unigene315_All | 3 269 Alcohol dehydrogenase class-P -like protein [Gossypium arboreum] | 10199.20764 | 0.607 | 1.25 |
| 101 | CL2167.Contig5_All | 9 1499 PREDICTED: lysosomal Pro-X carboxypeptidase-like [Vitis vinifera] | 57057.26126 | 0.022 | 1.57 |
| 102 | Unigene34539_All | 68 1150 unknown [Picea sitchensis] | 40012.56567 | 0.089 | 1.37 |
| 103 | Unigene33724_All | 115 1251 minus strand hypothetical protein POPTR_0343s00200g [Populus trichocarpa] &gt;gi\|550309463\|gb\|ERP47008.1\| hypothetical protein POPTR_0343s00200g [Populus trichocarpa] | 40853.90094 | 0.296 | 1.33 |
| 104 | Unigene35_All | 77 520 hypothetical protein PRUPE_ppa012937mg [Prunus persica] &gt;gi\|595830537\|ref\|XP_007206095.1\| hypothetical protein PRUPE_ppa012937mg [Prunus persica] &gt;gi\|462401736\|gb\|EMJ07293.1\| hypothetical protein PRUPE_ppa012937mg [Prunus persica] &gt;gi\|462401737\|gb\|EMJ07294.1\| hypothetical protein PRUPE_ppa012937mg [Prunus persica] | 16211.98415 | 0.926 | 2.1 |
| 105 | CL7605.Contig1_All | 47 871 Vacuolar protein sorting 45 [Theobroma cacao] &gt;gi\|508724731\|gb\|EOY16628.1\| Vacuolar protein sorting 45 [Theobroma cacao] | 31377.1453 | 0.047 | 1.52 |
| 106 | CL1864.Contig1_All | 250 1713 minus strand PREDICTED: ATP-dependent 6-phosphofructokinase 3-like [Nelumbo nucifera] | 54061.50862 | 0.33 | 1.25 |
| 107 | CL3341.Contig2_All | 513 872 Inner membrane protein ALBINO3, chloroplastic [Triticum urartu] | 12990.09209 | 0.083 | 1.5 |
| 108 | Unigene9508_All | 20 1120 PREDICTED: DNA-(apurinic or apyrimidinic site) lyase [Prunus mume] | 42215.34677 | 0.019 | 1.49 |
| 109 | Unigene16567_All | 740 2098 minus strand PREDICTED: protein NRT1/ PTR FAMILY 7.3 [Vitis vinifera] | 51606.02367 | 0.015 | 1.32 |
| 110 | Unigene30017_All | 134 505 minus strand PREDICTED: signal recognition particle 14 kDa protein-like [Sesamum indicum] | 14138.59838 | 0.145 | 1.22 |
| 111 | Unigene12043_All | 168 401 minus strand PREDICTED: aspartic proteinase nepenthesin-2-like [Camelina sativa] | 8587.588218 | 0.077 | 1.33 |
| 112 | Unigene6830_All | 116 451 minus strand PREDICTED: cytochrome c [Gossypium raimondii] &gt;gi\|763792673\|gb\|KJB59669.1\| hypothetical protein B456_009G266800 [Gossypium raimondii] | 12335.18094 | 0.286 | 1.39 |
| 113 | Unigene24835_All | 6 431 unknown [Picea sitchensis] &gt;gi\|148909086\|gb\|ABR17645.1\| unknown [Picea sitchensis] | 15347.0837 | 0.07 | 1.27 |
| 114 | CL10702.Contig2_All | 248 1612 PREDICTED: dihydroorotate dehydrogenase (quinone), mitochondrial [Nelumbo nucifera] | 48638.40528 | 0.026 | 1.32 |
| 115 | Unigene53630_All | 1 324 PREDICTED: triose phosphate/phosphate translocator, chloroplastic-like [Fragaria vesca subsp. vesca] | 12065.49212 | 0.046 | 1.32 |
| 116 | Unigene1046_All | 154 609 minus strand hypothetical protein POPTR_0005s24120g [Populus trichocarpa] &gt;gi\|118481495\|gb\|ABK92690.1\| unknown [Populus trichocarpa] &gt;gi\|118482312\|gb\|ABK93082.1\| unknown [Populus trichocarpa] &gt;gi\|222857081\|gb\|EEE94628.1\| hypothetical protein POPTR_0005s24120g [Populus trichocarpa] | 17165.27267 | 0.132 | 1.47 |
| 117 | CL12410.Contig11_All | 82 543 PREDICTED: probable histone H2B.1 [Vitis vinifera] &gt;gi\|147858650\|emb\|CAN78863.1\| hypothetical protein VITISV_032908 [Vitis vinifera] | 16554.22342 | 0.727 | 1.56 |
| 118 | Unigene6884_All | 88 1965 minus strand PREDICTED: ATP-dependent DNA helicase 2 subunit KU70 isoform X1 [Vitis vinifera] &gt;gi\|296089629\|emb\|CBI39448.3\| unnamed protein product [Vitis vinifera] | 70563.78672 | 0.014 | 1.38 |
| 119 | CL2213.Contig3_All | 242 1378 minus strand PREDICTED: alcohol dehydrogenase [Populus euphratica] | 41889.97953 | 0.24 | 1.34 |
| 120 | CL12325.Contig1_All | 230 1318 minus strand unnamed protein product [Vitis vinifera] | 41110.37732 | 0.044 | 1.41 |
| 121 | Unigene11080_All | 95 772 unknown [Picea sitchensis] &gt;gi\|116783111\|gb\|ABK22797.1\| unknown [Picea sitchensis] &gt;gi\|116786762\|gb\|ABK24227.1\| unknown [Picea sitchensis] &gt;gi\|224285131\|gb\|ACN40293.1\| unknown [Picea sitchensis] &gt;gi\|224285269\|gb\|ACN40360.1\| unknown [Picea sitchensis] | 25986.7081 | 0.04 | 1.32 |
| 122 | CL1864.Contig6_All | 204 1625 minus strand hypothetical protein PRUPE_ppa004610mg [Prunus persica] &gt;gi\|645226639\|ref\|XP_008220132.1\| PREDICTED: 6-phosphofructokinase 6 [Prunus mume] &gt;gi\|462420318\|gb\|EMJ24581.1\| hypothetical protein PRUPE_ppa004610mg [Prunus persica] | 52480.51731 | 0.173 | 1.53 |
| 123 | Unigene9877_All | 208 876 minus strand PREDICTED: vacuolar protein sorting-associated protein 2 homolog 1 [Sesamum indicum] | 25170.96784 | 0.045 | 1.45 |
| 124 | Unigene26892_All | 560 1354 PREDICTED: dolichyl-diphosphooligosaccharide--protein glycosyltransferase 48 kDa subunit [Nelumbo nucifera] | 29990.36005 | 0.268 | 1.43 |
| 125 | Unigene28012_All | 123 533 minus strand hypothetical protein SETIT_018284mg [Setaria italica] | 15564.63966 | 0.35 | 1.57 |
| 126 | CL12313.Contig1_All | 45 521 acyl-CoA thioesterase, putative [Ricinus communis] | 17300.04669 | 0.044 | 1.41 |
| 127 | Unigene47_All | 50 496 PREDICTED: probable histone H2B.1 [Vitis vinifera] &gt;gi\|147858650\|emb\|CAN78863.1\| hypothetical protein VITISV_032908 [Vitis vinifera] | 16146.93378 | 0.671 | 1.41 |
| 128 | CL5309.Contig2_All | 40 1752 PREDICTED: laccase-2-like [Populus euphratica] | 63534.56623 | 0.033 | 1.25 |
| 129 | Unigene26479_All | 84 533 minus strand 40S ribosomal protein S14-3 [Monoraphidium neglectum] &gt;gi\|761968579\|gb\|KIY99771.1\| 40S ribosomal protein S14-3 [Monoraphidium neglectum] | 15903.45073 | 0.113 | 1.28 |
| 130 | CL11092.Contig1_All | 154 552 minus strand RecName: Full=Probable histone H2A.3 &gt;gi\|158513647\|sp\|A2YVE5.1\|H2A3_ORYSI RecName: Full=Probable histone H2A.3 &gt;gi\|29837243\|dbj\|BAC75621.1\| putative histone H2A [Oryza sativa Japonica Group] &gt;gi\|38175493\|dbj\|BAD01189.1\| putative histone H2A [Oryza sativa Japonica Group] &gt;gi\|125561608\|gb\|EAZ07056.1\| hypothetical protein OsI_29303 [Oryza sativa Indica Group] &gt;gi\|125603477\|gb\|EAZ42802.1\| hypothetical protein OsJ_27388 [Oryza sativa Japonica Group] &gt;gi\|937930605\|dbj\|BAT05521.1\| Os08g0427700 [Oryza sativa Japonica Group] | 13867.57593 | 0.113 | 1.39 |
| 131 | Unigene29244_All | 56 487 predicted protein [Hordeum vulgare subsp. vulgare] | 15445.66723 | 0.354 | 2.11 |
| 132 | CL3846.Contig4_All | 133 1206 minus strand hypothetical protein CISIN_1g018326mg [Citrus sinensis] | 38850.85027 | 0.788 | 1.37 |
| 133 | Unigene19720_All | 14 1360 minus strand PREDICTED: protein trichome birefringence-like 3 [Vitis vinifera] &gt;gi\|296083173\|emb\|CBI22809.3\| unnamed protein product [Vitis vinifera] | 51866.9373 | 0.024 | 2.83 |
| 134 | Unigene26451_All | 259 1533 PREDICTED: vacuole membrane protein KMS1 [Vitis vinifera] &gt;gi\|297737027\|emb\|CBI26228.3\| unnamed protein product [Vitis vinifera] | 47685.19984 | 0.019 | 1.24 |
| 135 | CL5354.Contig2_All | 3 290 minus strand predicted protein [Hordeum vulgare subsp. vulgare] | 10949.0653 | 0.062 | 1.54 |
| 136 | Unigene84429_All | 37 477 minus strand PREDICTED: probable histone H2B.1 [Malus domestica] &gt;gi\|658018994\|ref\|XP_008344843.1\| PREDICTED: probable histone H2B.1 [Malus domestica] &gt;gi\|658028562\|ref\|XP_008349713.1\| PREDICTED: probable histone H2B.1 [Malus domestica] &gt;gi\|694354102\|ref\|XP_009358338.1\| PREDICTED: probable histone H2B.1 [Pyrus x bretschneideri] &gt;gi\|694354144\|ref\|XP_009358349.1\| PREDICTED: probable histone H2B.1 [Pyrus x bretschneideri] &gt;gi\|694420970\|ref\|XP_009338362.1\| PREDICTED: probable histone H2B.1 [Pyrus x bretschneideri] | 16150.979 | 0.823 | 1.81 |
| 137 | CL806.Contig1_All | 599 1543 S-adenosylmethionine carrier 1 [Theobroma cacao] &gt;gi\|508700421\|gb\|EOX92317.1\| S-adenosylmethionine carrier 1 [Theobroma cacao] | 33696.12678 | 0.079 | 1.63 |
| 138 | Unigene60277_All | 2 187 predicted protein [Physcomitrella patens] &gt;gi\|162688029\|gb\|EDQ74408.1\| predicted protein [Physcomitrella patens] | 6908.743899 | 0.129 | 1.27 |
| 139 | CL10784.Contig1_All | 145 549 PREDICTED: uncharacterized protein LOC100241624 [Vitis vinifera] | 15185.20014 | 0.17 | 1.65 |
| 140 | Unigene19401_All | 981 1517 clp protease proteolytic subunit (chloroplast) [Paeonia obovata] &gt;gi\|573015339\|gb\|AHF71919.1\| clpP-like protease (chloroplast) [Paeonia sp. Sd0052] &gt;gi\|604723705\|gb\|AHV83421.1\| clp protease proteolytic subunit (chloroplast) [Paeonia obovata] | 20180.3156 | 0.078 | 1.29 |
| 141 | CL9780.Contig3_All | 97 888 minus strand PREDICTED: chlorophyll a-b binding protein 151, chloroplastic [Nelumbo nucifera] | 28555.41775 | 0.386 | 1.44 |
| 142 | Unigene23301_All | 114 806 putative 2C-methyl-D-erythritol 2,4-cyclodiphosphate synthase [Hevea brasiliensis] | 25032.21505 | 0.074 | 1.54 |
| 143 | Unigene19817_All | 299 988 PREDICTED: peptidyl-prolyl cis-trans isomerase CYP21-4 [Vitis vinifera] &gt;gi\|296083251\|emb\|CBI22887.3\| unnamed protein product [Vitis vinifera] | 25746.55795 | 0.117 | 1.38 |
| 144 | Unigene1074_All | 76 462 unnamed protein product [Vitis vinifera] | 14351.93543 | 0.419 | 1.23 |
| 145 | Unigene33812_All | 217 1578 minus strand PREDICTED: probable glucuronoxylan glucuronosyltransferase F8H [Nelumbo nucifera] | 52336.73299 | 0.018 | 2 |
| 146 | Unigene902_All | 142 852 minus strand PREDICTED: aquaporin SIP1-2-like [Malus domestica] &gt;gi\|658043154\|ref\|XP_008357208.1\| PREDICTED: aquaporin SIP1-2-like [Malus domestica] | 25246.39077 | 0.139 | 1.23 |
| 147 | CL9577.Contig2_All | 3 212 minus strand class 4 pathogenesis-related family protein [Populus trichocarpa] &gt;gi\|118487978\|gb\|ABK95810.1\| unknown [Populus trichocarpa] &gt;gi\|222857453\|gb\|EEE95000.1\| class 4 pathogenesis-related family protein [Populus trichocarpa] | 7457.626281 | 0.186 | 1.28 |
| 148 | Unigene290_All | 109 4473 minus strand PREDICTED: pleiotropic drug resistance protein 1-like [Nelumbo nucifera] | 165114.4987 | 0.025 | 1.4 |
| 149 | Unigene27199_All | 67 414 non-specific lipid transfer protein [Vitis vinifera] | 12040.94145 | 0.284 | 2.26 |
| 150 | Unigene27060_All | 198 1013 minus strand Photosystem II 22 kDa family protein [Populus trichocarpa] &gt;gi\|118488707\|gb\|ABK96164.1\| unknown [Populus trichocarpa] &gt;gi\|222842713\|gb\|EEE80260.1\| Photosystem II 22 kDa family protein [Populus trichocarpa] | 28731.36637 | 0.158 | 1.23 |
| 151 | Unigene87205_All | 2 253 PREDICTED: putative pentatricopeptide repeat-containing protein At5g08490 [Camelina sativa] | 9483.512534 | 0.119 | 1.53 |
| 152 | Unigene9554_All | 72 1541 PREDICTED: dnaJ homolog 1, mitochondrial [Vitis vinifera] &gt;gi\|297743480\|emb\|CBI36347.3\| unnamed protein product [Vitis vinifera] | 53716.2318 | 0.024 | 1.33 |
| 153 | Unigene10075_All | 79 507 minus strand PREDICTED: 40S ribosomal protein S19-3 [Populus euphratica] | 16024.48367 | 0.392 | 1.26 |
| 154 | CL12478.Contig6_All | 103 549 hypothetical protein EUTSA_v10019117mg [Eutrema salsugineum] &gt;gi\|557087266\|gb\|ESQ28118.1\| hypothetical protein EUTSA_v10019117mg [Eutrema salsugineum] | 16611.3122 | 0.356 | 1.25 |
| 155 | CL2933.Contig2_All | 53 2047 PREDICTED: probable cadmium/zinc-transporting ATPase HMA1, chloroplastic isoform X1 [Vitis vinifera] &gt;gi\|296087394\|emb\|CBI33768.3\| unnamed protein product [Vitis vinifera] | 71779.23444 | 0.014 | 1.22 |
| 156 | Unigene26547_All | 175 1053 minus strand PREDICTED: probable xyloglucan endotransglucosylase/hydrolase protein 6 [Gossypium raimondii] &gt;gi\|763750704\|gb\|KJB18092.1\| hypothetical protein B456_003G033600 [Gossypium raimondii] | 34069.69845 | 0.218 | 1.22 |
| 157 | Unigene30223_All | 193 588 PREDICTED: thioredoxin H2-like [Fragaria vesca subsp. vesca] | 14809.43901 | 0.091 | 1.26 |
| 158 | Unigene26634_All | 72 479 PREDICTED: 40S ribosomal protein S17-4-like [Eucalyptus grandis] | 15351.24888 | 0.279 | 1.25 |
| 159 | Unigene10175_All | 399 1958 PREDICTED: calcium-dependent protein kinase 29 [Sesamum indicum] | 59039.89507 | 0.087 | 1.23 |
| 160 | CL10469.Contig6_All | 543 2741 hypothetical protein CICLE_v10030804mg [Citrus clementina] &gt;gi\|568859295\|ref\|XP_006483176.1\| PREDICTED: cullin-3A isoform X1 [Citrus sinensis] &gt;gi\|557540867\|gb\|ESR51911.1\| hypothetical protein CICLE_v10030804mg [Citrus clementina] | 85619.74854 | 0.035 | 1.23 |
| 161 | CL12114.Contig3_All | 230 1021 hypothetical protein CISIN_1g020966mg [Citrus sinensis] | 28492.32374 | 0.034 | 1.21 |
| 162 | Unigene13684_All | 154 942 PREDICTED: ATP-dependent Clp protease proteolytic subunit 2, mitochondrial [Ricinus communis] &gt;gi\|223536980\|gb\|EEF38617.1\| ATP-dependent Clp protease proteolytic subunit, putative [Ricinus communis] | 29113.8151 | 0.106 | 1.33 |
| 163 | Unigene13555_All | 291 1586 minus strand PREDICTED: putative uncharacterized protein DDB_G0281733 [Prunus mume] | 48927.40162 | 0.079 | 1.3 |
| 164 | Unigene10030_All | 102 590 PREDICTED: 60S ribosomal protein L24 [Jatropha curcas] &gt;gi\|802769218\|ref\|XP_012090295.1\| PREDICTED: 60S ribosomal protein L24 [Jatropha curcas] &gt;gi\|643706180\|gb\|KDP22312.1\| hypothetical protein JCGZ_26143 [Jatropha curcas] | 18574.4398 | 0.344 | 1.68 |
| 165 | CL4679.Contig2_All | 189 935 minus strand PREDICTED: aquaporin TIP1-3 [Nicotiana tomentosiformis] | 25824.43893 | 0.08 | 1.33 |
| 166 | Unigene23530_All | 127 3246 sucrose-phosphate synthase 1 [Vitis vinifera] &gt;gi\|58825798\|gb\|AAW82754.1\| sucrose-phosphate synthase 1 [Vitis vinifera] | 117752.8939 | 0.064 | 1.29 |
| 167 | CL1192.Contig1_All | 129 1025 minus strand PREDICTED: (+)-neomenthol dehydrogenase-like isoform X1 [Vitis vinifera] | 32518.78004 | 0.224 | 1.23 |
| 168 | CL3248.Contig1_All | 444 1397 minus strand PREDICTED: peroxisome biogenesis protein 7 [Cucumis sativus] &gt;gi\|700194105\|gb\|KGN49309.1\| hypothetical protein Csa_6G519610 [Cucumis sativus] | 36023.43931 | 0.035 | 1.25 |
| 169 | Unigene23909_All | 1 1974 minus strand ATPase 11, plasma membrane-type [Morus notabilis] &gt;gi\|587947943\|gb\|EXC34215.1\| ATPase 11, plasma membrane-type [Morus notabilis] | 73224.58413 | 0.213 | 1.37 |
| 170 | CL1102.Contig11_All | 268 1281 minus strand PREDICTED: GDSL esterase/lipase 4-like [Eucalyptus grandis] &gt;gi\|629090162\|gb\|KCW56415.1\| hypothetical protein EUGRSUZ_I02141 [Eucalyptus grandis] | 37658.74007 | 0.038 | 1.35 |
| 171 | CL12478.Contig1_All | 119 562 hypothetical protein EUTSA_v10019117mg [Eutrema salsugineum] &gt;gi\|557087266\|gb\|ESQ28118.1\| hypothetical protein EUTSA_v10019117mg [Eutrema salsugineum] | 16527.39237 | 0.466 | 1.21 |
| 172 | Unigene7110_All | 190 1119 PREDICTED: very-long-chain enoyl-CoA reductase [Ricinus communis] &gt;gi\|223529574\|gb\|EEF31524.1\| Synaptic glycoprotein SC2, putative [Ricinus communis] | 36513.99995 | 0.032 | 1.22 |
| 173 | CL2719.Contig1_All | 174 1424 minus strand DNAJ [Theobroma cacao] &gt;gi\|508727397\|gb\|EOY19294.1\| DNAJ [Theobroma cacao] | 47051.07598 | 0.319 | 1.3 |
| 174 | Unigene1170_All | 225 1016 PREDICTED: reticulon-like protein B5 [Nicotiana sylvestris] | 29921.75702 | 0.072 | 1.46 |
| 175 | Unigene33449_All | 127 729 ATPase, F1 complex, delta/epsilon subunit [Theobroma cacao] &gt;gi\|508776863\|gb\|EOY24119.1\| ATPase, F1 complex, delta/epsilon subunit [Theobroma cacao] | 21580.37523 | 0.174 | 1.23 |
| 176 | CL4943.Contig1_All | 156 1502 PREDICTED: uncharacterized protein LOC100248242 [Vitis vinifera] | 48993.16774 | 0.022 | 1.23 |
| 177 | Unigene1179_All | 293 1351 minus strand pyridine nucleotide-disulfide oxidoreductase family protein [Populus trichocarpa] &gt;gi\|222847037\|gb\|EEE84584.1\| pyridine nucleotide-disulfide oxidoreductase family protein [Populus trichocarpa] | 38910.76272 | 0.028 | 1.36 |
| 178 | CL4758.Contig2_All | 12 1685 Putative 2-3 biphosphoglycerate independant phosphoglycerate mutase [Vitis vinifera] | 61580.27602 | 0.075 | 1.21 |
| 179 | Unigene65210_All | 59 1006 minus strand hypothetical protein CHLNCDRAFT_59607 [Chlorella variabilis] &gt;gi\|307110053\|gb\|EFN58290.1\| hypothetical protein CHLNCDRAFT_59607 [Chlorella variabilis] | 34557.01838 | 0.022 | 0.55 |
| 180 | Unigene26939_All | 321 2132 minus strand ethylene insensitive 3-like 2 protein [Paeonia lactiflora] | 68983.26292 | 0.018 | 0.56 |
| 181 | Unigene8126_All | 2 724 minus strand PREDICTED: probable boron transporter 2 [Sesamum indicum] | 27384.01499 | 0.025 | 0.56 |
| 182 | Unigene13624_All | 201 1142 minus strand PREDICTED: pectinesterase 31 [Vitis vinifera] &gt;gi\|296082952\|emb\|CBI22253.3\| unnamed protein product [Vitis vinifera] | 36150.55233 | 0.041 | 0.57 |
| 183 | Unigene64515_All | 1 240 minus strand PREDICTED: LOW QUALITY PROTEIN: proteasome subunit alpha type-3 [Ricinus communis] | 8939.6537 | 0.075 | 0.58 |
| 184 | Unigene33216_All | 124 1620 hexose transport protein [Actinidia chinensis] | 54428.90376 | 0.024 | 0.6 |
| 185 | Unigene10793_All | 23 484 minus strand unnamed protein product [Vitis vinifera] | 17849.65497 | 0.266 | 0.6 |
| 186 | Unigene13203_All | 120 662 PREDICTED: uncharacterized protein LOC104595729 [Nelumbo nucifera] &gt;gi\|719996814\|ref\|XP_010254893.1\| PREDICTED: uncharacterized protein LOC104595729 [Nelumbo nucifera] | 20179.39275 | 0.077 | 0.61 |
| 187 | CL12515.Contig5_All | 197 1273 PREDICTED: probable mannitol dehydrogenase [Sesamum indicum] | 39671.09245 | 0.081 | 0.61 |
| 188 | CL11000.Contig2_All | 63 1541 PREDICTED: U-box domain-containing protein 11 [Vitis vinifera] | 54285.59599 | 0.01 | 0.65 |
| 189 | Unigene33649_All | 219 2000 minus strand PREDICTED: probable methyltransferase PMT7 isoform X1 [Ricinus communis] &gt;gi\|223526426\|gb\|EEF28705.1\| ATP binding protein, putative [Ricinus communis] | 69436.15367 | 0.015 | 0.65 |
| 190 | Unigene23068_All | 667 1599 PREDICTED: probable aquaporin NIP5-1 [Jatropha curcas] &gt;gi\|643711764\|gb\|KDP25192.1\| hypothetical protein JCGZ_20348 [Jatropha curcas] | 32570.9911 | 0.032 | 0.65 |
| 191 | Unigene9864_All | 81 725 PREDICTED: transmembrane emp24 domain-containing protein p24beta3 [Vitis vinifera] &gt;gi\|297737114\|emb\|CBI26315.3\| unnamed protein product [Vitis vinifera] | 24817.46913 | 0.051 | 0.66 |
| 192 | CL5359.Contig3_All | 2 628 minus strand hypothetical protein VITISV_012280 [Vitis vinifera] | 23174.07066 | 0.292 | 0.66 |
| 193 | CL3832.Contig1_All | 1000 2622 PREDICTED: scarecrow-like protein 21 [Vitis vinifera] &gt;gi\|731433127\|ref\|XP_010644523.1\| PREDICTED: scarecrow-like protein 21 [Vitis vinifera] | 60699.81211 | 0.018 | 0.67 |
| 194 | CL5085.Contig3_All | 150 1763 minus strand PREDICTED: mediator of RNA polymerase II transcription subunit 25 isoform X1 [Jatropha curcas] | 57078.85717 | 0.026 | 0.67 |
| 195 | Unigene23009_All | 544 2682 PREDICTED: probable boron transporter 2 [Vitis vinifera] &gt;gi\|297744033\|emb\|CBI37003.3\| unnamed protein product [Vitis vinifera] | 80079.93687 | 0.013 | 0.68 |
| 196 | Unigene32955_All | 26 1504 PREDICTED: hexokinase-2, chloroplastic [Ricinus communis] &gt;gi\|223551227\|gb\|EEF52713.1\| hexokinase, putative [Ricinus communis] | 53882.35961 | 0.057 | 0.68 |
| 197 | Unigene30998_All | 138 2087 PREDICTED: heat shock cognate 70 kDa protein 2-like [Sesamum indicum] | 71775.21816 | 0.388 | 0.68 |
| 198 | Unigene12444_All | 154 1491 tubulin beta-1 chain [Medicago truncatula] &gt;gi\|355481332\|gb\|AES62535.1\| tubulin beta-1 chain [Medicago truncatula] | 50896.09277 | 0.442 | 0.68 |
| 199 | Unigene33723_All | 227 1258 WD40 protein [Paeonia suffruticosa] | 39024.27275 | 0.044 | 0.69 |
| 200 | CL1121.Contig4_All | 3543 6173 minus strand PREDICTED: alpha-xylosidase 1 [Vitis vinifera] | 98683.19115 | 0.089 | 0.69 |
| 201 | CL6016.Contig1_All | 280 2298 PREDICTED: probable metal-nicotianamine transporter YSL6 [Vitis vinifera] &gt;gi\|296082320\|emb\|CBI21325.3\| unnamed protein product [Vitis vinifera] | 74463.12511 | 0.012 | 0.7 |
| 202 | CL2560.Contig1_All | 41 946 PREDICTED: telomere repeat-binding factor 1 [Jatropha curcas] &gt;gi\|643738591\|gb\|KDP44512.1\| hypothetical protein JCGZ_16345 [Jatropha curcas] | 33054.4115 | 0.023 | 0.7 |
| 203 | CL3224.Contig4_All | 2 529 hypothetical protein PRUPE_ppa006082mg [Prunus persica] &gt;gi\|462404896\|gb\|EMJ10360.1\| hypothetical protein PRUPE_ppa006082mg [Prunus persica] | 20162.53871 | 0.074 | 0.7 |
| 204 | CL5602.Contig1_All | 204 707 PREDICTED: ubiquitin-conjugating enzyme E2 13 [Vitis vinifera] &gt;gi\|297740393\|emb\|CBI30575.3\| unnamed protein product [Vitis vinifera] | 18950.46923 | 0.089 | 0.7 |
| 205 | CL384.Contig1_All | 143 1228 PREDICTED: probable mannitol dehydrogenase [Vitis vinifera] &gt;gi\|297741856\|emb\|CBI33216.3\| unnamed protein product [Vitis vinifera] | 39685.02002 | 0.569 | 0.7 |
| 206 | Unigene33895_All | 173 1921 hypothetical protein PRUPE_ppa003339mg [Prunus persica] &gt;gi\|462419231\|gb\|EMJ23494.1\| hypothetical protein PRUPE_ppa003339mg [Prunus persica] | 65296.30172 | 0.017 | 0.71 |
| 207 | Unigene86242_All | 3 302 hypothetical protein VITISV_025837 [Vitis vinifera] | 11614.02481 | 0.15 | 0.71 |
| 208 | Unigene17074_All | 182 1192 Homocysteine S-methyltransferase 3 [Theobroma cacao] &gt;gi\|508713857\|gb\|EOY05754.1\| Homocysteine S-methyltransferase 3 [Theobroma cacao] | 37430.54052 | 0.024 | 0.72 |
| 209 | CL8907.Contig1_All | 360 1028 PREDICTED: uncharacterized protein LOC100255501 [Vitis vinifera] &gt;gi\|296087051\|emb\|CBI33378.3\| unnamed protein product [Vitis vinifera] | 24372.68851 | 0.036 | 0.72 |
| 210 | Unigene24674_All | 47 787 unknown [Picea sitchensis] | 25689.9083 | 0.065 | 0.72 |
| 211 | Unigene26671_All | 221 682 PREDICTED: ubiquitin-conjugating enzyme E2-17 kDa [Cucumis melo] | 17327.73442 | 0.123 | 0.72 |
| 212 | CL8500.Contig1_All | 92 2503 minus strand Exocyst complex component sec15B isoform 1 [Theobroma cacao] &gt;gi\|590719131\|ref\|XP_007050969.1\| Exocyst complex component sec15B isoform 1 [Theobroma cacao] &gt;gi\|508703229\|gb\|EOX95125.1\| Exocyst complex component sec15B isoform 1 [Theobroma cacao] &gt;gi\|508703230\|gb\|EOX95126.1\| Exocyst complex component sec15B isoform 1 [Theobroma cacao] | 91504.62567 | 0.012 | 0.73 |
| 213 | CL5406.Contig1_All | 279 1355 minus strand PREDICTED: malate dehydrogenase, glyoxysomal isoform X1 [Vitis vinifera] | 38297.03706 | 0.237 | 0.73 |
| 214 | Unigene30566_All | 289 3435 minus strand PREDICTED: calcium-transporting ATPase, endoplasmic reticulum-type [Vitis vinifera] &gt;gi\|731422082\|ref\|XP_010661979.1\| PREDICTED: calcium-transporting ATPase, endoplasmic reticulum-type [Vitis vinifera] &gt;gi\|731422084\|ref\|XP_010661980.1\| PREDICTED: calcium-transporting ATPase, endoplasmic reticulum-type [Vitis vinifera] | 117742.1359 | 0.019 | 0.74 |
| 215 | Unigene26477_All | 244 2094 minus strand PREDICTED: BTB/POZ domain-containing protein At5g48800 [Jatropha curcas] | 70460.58257 | 0.023 | 0.74 |
| 216 | Unigene2850_All | 7 279 PREDICTED: eukaryotic translation initiation factor 3 subunit I-like isoform X2 [Nicotiana sylvestris] | 10284.84552 | 0.088 | 0.74 |
| 217 | Unigene30768_All | 47 970 minus strand hypothetical protein SORBIDRAFT_02g039270 [Sorghum bicolor] &gt;gi\|241926559\|gb\|EER99703.1\| hypothetical protein SORBI_002G375100 [Sorghum bicolor] | 33455.92163 | 0.039 | 0.75 |
| 218 | Unigene13500_All | 332 1273 PREDICTED: nicotinamide adenine dinucleotide transporter 1, chloroplastic [Vitis vinifera] &gt;gi\|297743935\|emb\|CBI36905.3\| unnamed protein product [Vitis vinifera] | 34482.82129 | 0.045 | 0.75 |
| 219 | Unigene11020_All | 128 2569 minus strand PREDICTED: phospholipase D alpha 1 [Amborella trichopoda] &gt;gi\|548844118\|gb\|ERN03744.1\| hypothetical protein AMTR_s00078p00051300 [Amborella trichopoda] | 92884.66867 | 0.049 | 0.75 |
| 220 | Unigene17102_All | 241 1194 minus strand hypothetical protein B456_007G233400 [Gossypium raimondii] | 33658.87931 | 0.063 | 0.75 |
| 221 | CL1552.Contig4_All | 347 1822 PREDICTED: probable serine/threonine-protein kinase At5g41260 [Vitis vinifera] &gt;gi\|297745973\|emb\|CBI16029.3\| unnamed protein product [Vitis vinifera] | 55556.29496 | 0.065 | 0.75 |
| 222 | Unigene20433_All | 539 1147 PREDICTED: uncharacterized protein LOC100267343 [Vitis vinifera] &gt;gi\|297746307\|emb\|CBI16363.3\| unnamed protein product [Vitis vinifera] | 23475.23106 | 0.069 | 0.75 |
| 223 | Unigene23562_All | 364 1302 Peroxidase superfamily protein [Theobroma cacao] &gt;gi\|508700137\|gb\|EOX92033.1\| Peroxidase superfamily protein [Theobroma cacao] | 35138.59933 | 0.086 | 0.75 |
| 224 | Unigene9273_All | 3 404 hypothetical protein SOVF_052440 [Spinacia oleracea] | 14093.28269 | 0.157 | 0.75 |
| 225 | CL8209.Contig1_All | 501 1574 hypothetical protein LR48_Vigan02g151400 [Vigna angularis] &gt;gi\|965608744\|dbj\|BAT95235.1\| hypothetical protein VIGAN_08191700 [Vigna angularis var. angularis] | 39242.70343 | 0.162 | 0.75 |
| 226 | CL11021.Contig3_All | 118 1188 PREDICTED: UDP-arabinopyranose mutase 1 [Vitis vinifera] | 41156.77351 | 0.162 | 0.75 |
| 227 | CL3343.Contig3_All | 561 3875 minus strand PREDICTED: uncharacterized protein LOC100262596 isoform X1 [Vitis vinifera] | 123641.3342 | 0.007 | 0.76 |
| 228 | Unigene16946_All | 394 2280 minus strand PREDICTED: xyloglucan galactosyltransferase KATAMARI1-like [Pyrus x bretschneideri] | 71761.55469 | 0.014 | 0.76 |
| 229 | Unigene13452_All | 16 3126 minus strand hypothetical protein EUGRSUZ_C00246 [Eucalyptus grandis] | 118620.5938 | 0.014 | 0.76 |
| 230 | Unigene20303_All | 211 2478 minus strand PREDICTED: 6-phosphofructo-2-kinase/fructose-2,6-bisphosphatase [Vitis vinifera] &gt;gi\|297745867\|emb\|CBI15923.3\| unnamed protein product [Vitis vinifera] | 84156.7809 | 0.016 | 0.76 |
| 231 | Unigene30298_All | 274 1365 PREDICTED: probable protein phosphatase 2C 34 [Citrus sinensis] &gt;gi\|568836229\|ref\|XP_006472148.1\| PREDICTED: probable protein phosphatase 2C 34 [Citrus sinensis] | 41028.92628 | 0.022 | 0.76 |
| 232 | CL4425.Contig2_All | 390 1607 alpha-amylase [Hevea brasiliensis] | 46419.77491 | 0.032 | 0.76 |
| 233 | CL7087.Contig2_All | 264 1589 PREDICTED: glucan endo-1,3-beta-glucosidase 2 [Ricinus communis] &gt;gi\|223528432\|gb\|EEF30466.1\| Glucan endo-1,3-beta-glucosidase precursor, putative [Ricinus communis] | 47761.80363 | 0.036 | 0.76 |
| 234 | Unigene13707_All | 156 1142 minus strand PREDICTED: ubiquitin carboxyl-terminal hydrolase isozyme L5 [Vigna radiata var. radiata] | 37739.90166 | 0.046 | 0.76 |
| 235 | CL7441.Contig2_All | 286 1503 minus strand PREDICTED: CMP-sialic acid transporter 2 [Solanum pennellii] &gt;gi\|970052105\|ref\|XP_015087650.1\| PREDICTED: CMP-sialic acid transporter 2 [Solanum pennellii] | 44897.04785 | 0.062 | 0.76 |
| 236 | CL4209.Contig1_All | 535 2631 PREDICTED: serine/threonine-protein kinase BLUS1 isoform X1 [Citrus sinensis] &gt;gi\|568832176\|ref\|XP_006470318.1\| PREDICTED: serine/threonine-protein kinase BLUS1 isoform X1 [Citrus sinensis] &gt;gi\|568832178\|ref\|XP_006470319.1\| PREDICTED: serine/threonine-protein kinase BLUS1 isoform X1 [Citrus sinensis] &gt;gi\|568832180\|ref\|XP_006470320.1\| PREDICTED: serine/threonine-protein kinase BLUS1 isoform X1 [Citrus sinensis] &gt;gi\|641847255\|gb\|KDO66135.1\| hypothetical protein CISIN_1g005455mg [Citrus sinensis] &gt;gi\|641847256\|gb\|KDO66136.1\| hypothetical protein CISIN_1g005455mg [Citrus sinensis] &gt;gi\|641847257\|gb\|KDO66137.1\| hypothetical protein CISIN_1g005455mg [Citrus sinensis] &gt;gi\|641847258\|gb\|KDO66138.1\| hypothetical protein CISIN_1g005455mg [Citrus sinensis] &gt;gi\|641847259\|gb\|KDO66139.1\| hypothetical protein CISIN_1g005455mg [Citrus sinensis] &gt;gi\|641847260\|gb\|KDO66140.1\| hypothetical protein CISIN_1g005455mg [Citrus sinensis] | 78375.38277 | 0.064 | 0.76 |
| 237 | CL7778.Contig3_All | 85 1083 Alpha-galactosidase 2 [Theobroma cacao] &gt;gi\|508707082\|gb\|EOX98978.1\| Alpha-galactosidase 2 [Theobroma cacao] | 36814.8657 | 0.027 | 0.77 |
| 238 | Unigene23612_All | 101 1690 minus strand hypothetical protein PRUPE_ppa004132mg [Prunus persica] &gt;gi\|462418750\|gb\|EMJ23013.1\| hypothetical protein PRUPE_ppa004132mg [Prunus persica] | 58638.09147 | 0.032 | 0.77 |
| 239 | CL2492.Contig11_All | 486 3044 minus strand PREDICTED: phospholipase D beta 1-like [Vitis vinifera] &gt;gi\|731379694\|ref\|XP_010661435.1\| PREDICTED: phospholipase D beta 1-like [Vitis vinifera] | 96775.6803 | 0.036 | 0.77 |
| 240 | CL10612.Contig2_All | 167 778 minus strand PREDICTED: charged multivesicular body protein 1b [Vitis vinifera] &gt;gi\|147855645\|emb\|CAN79153.1\| hypothetical protein VITISV_016190 [Vitis vinifera] | 22657.45088 | 0.049 | 0.77 |
| 241 | CL48.Contig2_All | 203 1057 PREDICTED: uncharacterized protein LOC100255748 [Vitis vinifera] | 32489.78446 | 0.049 | 0.77 |
| 242 | CL2636.Contig5_All | 166 1665 hypothetical protein CICLE_v10004812mg [Citrus clementina] &gt;gi\|557522283\|gb\|ESR33650.1\| hypothetical protein CICLE_v10004812mg [Citrus clementina] | 54076.25063 | 0.056 | 0.77 |
| 243 | Unigene6619_All | 10 918 minus strand PREDICTED: auxilin-related protein 2-like isoform X4 [Populus euphratica] | 36598.18618 | 0.059 | 0.77 |
| 244 | Unigene22821_All | 388 1137 PREDICTED: glycine-rich protein 2-like isoform X1 [Phoenix dactylifera] | 24285.91678 | 0.088 | 0.77 |
| 245 | Unigene23033_All | 93 1802 minus strand PREDICTED: protease Do-like 10, mitochondrial [Populus euphratica] | 63822.71323 | 0.012 | 0.78 |
| 246 | Unigene26039_All | 308 1705 hypothetical protein PRUPE_ppa004814mg [Prunus persica] &gt;gi\|462419282\|gb\|EMJ23545.1\| hypothetical protein PRUPE_ppa004814mg [Prunus persica] | 50908.92634 | 0.017 | 0.78 |
| 247 | Unigene13946_All | 196 1659 minus strand PREDICTED: serine carboxypeptidase-like [Amborella trichopoda] &gt;gi\|548854979\|gb\|ERN12871.1\| hypothetical protein AMTR_s00050p00101210 [Amborella trichopoda] | 54986.86963 | 0.029 | 0.78 |
| 248 | CL2101.Contig2_All | 37 1641 PREDICTED: thioredoxin reductase NTRC [Vitis vinifera] &gt;gi\|731399842\|ref\|XP_010653765.1\| PREDICTED: thioredoxin reductase NTRC [Vitis vinifera] &gt;gi\|731399844\|ref\|XP_010653766.1\| PREDICTED: thioredoxin reductase NTRC [Vitis vinifera] | 59045.9787 | 0.034 | 0.78 |
| 249 | CL392.Contig7_All | 337 3285 PREDICTED: vacuolar protein sorting-associated protein 18 homolog [Vitis vinifera] &gt;gi\|296084966\|emb\|CBI28381.3\| unnamed protein product [Vitis vinifera] | 112308.5936 | 0.049 | 0.78 |
| 250 | Unigene35061_All | 3 443 minus strand ATPase subunit 4 [Citrullus lanatus] &gt;gi\|259156762\|gb\|ACV96624.1\| ATPase subunit 4 [Citrullus lanatus] | 17039.16104 | 0.109 | 0.78 |
| 251 | Unigene27602_All | 63 1538 catalase [Pinus sylvestris] | 57377.33854 | 0.11 | 0.78 |
| 252 | CL6839.Contig2_All | 237 1199 PREDICTED: 60S acidic ribosomal protein P0 [Vitis vinifera] | 34408.01426 | 0.215 | 0.78 |
| 253 | CL3480.Contig1_All | 238 1104 PREDICTED: alpha-soluble NSF attachment protein-like [Sesamum indicum] | 33031.46332 | 0.246 | 0.78 |
| 254 | CL3857.Contig1_All | 105 2357 PREDICTED: peroxisome biogenesis protein 5 isoform X1 [Vitis vinifera] &gt;gi\|296090052\|emb\|CBI39871.3\| unnamed protein product [Vitis vinifera] | 84442.91577 | 0.017 | 0.79 |
| 255 | CL8262.Contig1_All | 237 3515 minus strand PREDICTED: ubiquitin carboxyl-terminal hydrolase 26 [Vitis vinifera] | 124737.4285 | 0.018 | 0.79 |
| 256 | Unigene9381_All | 291 2321 minus strand PREDICTED: root phototropism protein 3 [Vitis vinifera] &gt;gi\|731434728\|ref\|XP_010645167.1\| PREDICTED: root phototropism protein 3 [Vitis vinifera] | 76749.67971 | 0.018 | 0.79 |
| 257 | Unigene30383_All | 229 1797 PREDICTED: oxalate--CoA ligase-like [Vitis vinifera] | 56678.32781 | 0.034 | 0.79 |
| 258 | Unigene1911_All | 63 764 class I chitinase [Dimocarpus longan] &gt;gi\|301323233\|gb\|ADK70388.1\| class I chitinase [Dimocarpus longan] | 26368.74852 | 0.034 | 0.79 |
| 259 | Unigene12830_All | 231 989 minus strand hypothetical protein POPTR_0017s08440g [Populus trichocarpa] &gt;gi\|550319776\|gb\|ERP50867.1\| hypothetical protein POPTR_0017s08440g [Populus trichocarpa] | 30048.11907 | 0.036 | 0.79 |
| 260 | Unigene31473_All | 116 841 minus strand putative histone H1 [Cryptomeria japonica] | 25340.75305 | 0.058 | 0.79 |
| 261 | CL10263.Contig2_All | 3 278 PREDICTED: acyl carrier protein 2, mitochondrial [Citrus sinensis] | 10604.29481 | 0.065 | 0.79 |
| 262 | Unigene13427_All | 109 465 minus strand unnamed protein product [Coffea canephora] | 13191.25945 | 0.168 | 0.79 |
| 263 | CL5916.Contig1_All | 84 500 PREDICTED: auxin-repressed 12.5 kDa protein-like isoform X2 [Camelina sativa] | 14522.25076 | 0.194 | 0.79 |
| 264 | Unigene10232_All | 75 839 minus strand hypothetical protein PRUPE_ppa010307mg [Prunus persica] &gt;gi\|462407760\|gb\|EMJ13094.1\| hypothetical protein PRUPE_ppa010307mg [Prunus persica] | 28377.90322 | 0.318 | 0.79 |
| 265 | Unigene6929_All | 131 1105 minus strand PREDICTED: cytochrome c1-2, heme protein, mitochondrial-like [Nicotiana tomentosiformis] | 36091.12735 | 0.329 | 0.79 |
| 266 | Unigene19960_All | 162 788 minus strand PREDICTED: membrane steroid-binding protein 1 [Ricinus communis] &gt;gi\|223549923\|gb\|EEF51410.1\| steroid binding protein, putative [Ricinus communis] | 22640.28 | 0.335 | 0.79 |
| 267 | Unigene30501_All | 110 730 PREDICTED: 60S ribosomal protein L13-1 [Sesamum indicum] | 23886.25439 | 0.459 | 0.79 |
| 268 | Unigene13220_All | 134 766 minus strand PREDICTED: transmembrane emp24 domain-containing protein p24delta9 [Vitis vinifera] | 24198.20732 | 0.033 | 0.8 |
| 269 | CL2338.Contig1_All | 3 548 PREDICTED: uncharacterized protein LOC104586371 [Nelumbo nucifera] | 20172.06353 | 0.06 | 0.8 |
| 270 | CL11021.Contig2_All | 152 1234 PREDICTED: alpha-1,4-glucan-protein synthase [UDP-forming] 2 [Vitis vinifera] | 41544.87831 | 0.133 | 0.8 |
| 271 | Unigene1034_All | 92 439 PREDICTED: NADH dehydrogenase [ubiquinone] 1 beta subcomplex subunit 9 [Populus euphratica] | 13488.88038 | 0.138 | 0.8 |
| 272 | CL6826.Contig4_All | 201 3038 minus strand unknown [Picea sitchensis] | 104435.6091 | 0.151 | 0.8 |
| 273 | CL12048.Contig2_All | 74 970 minus strand unnamed protein product [Vitis vinifera] | 33607.54858 | 0.151 | 0.8 |
| 274 | Unigene20392_All | 221 1021 PREDICTED: GEM-like protein 5 [Vitis vinifera] &gt;gi\|297738289\|emb\|CBI27490.3\| unnamed protein product [Vitis vinifera] | 29578.57756 | 0.251 | 0.8 |
| 275 | CL9208.Contig2_All | 142 885 PREDICTED: proteasome subunit alpha type-4 [Citrus sinensis] &gt;gi\|641831137\|gb\|KDO50206.1\| hypothetical protein CISIN_1g025640mg [Citrus sinensis] | 27394.74837 | 0.302 | 0.8 |
| 276 | CL61.Contig2_All | 79 831 BnaC03g69920D [Brassica napus] | 27790.8962 | 0.319 | 0.8 |
| 277 | Unigene12448_All | 128 1459 minus strand PREDICTED: tubulin beta chain-like [Malus domestica] &gt;gi\|658048166\|ref\|XP_008359774.1\| PREDICTED: tubulin beta chain [Malus domestica] &gt;gi\|694355740\|ref\|XP_009358821.1\| PREDICTED: tubulin beta chain-like [Pyrus x bretschneideri] | 50582.99706 | 0.405 | 0.8 |
| 278 | Unigene33138_All | 72 3476 PREDICTED: peroxisome biogenesis protein 1 isoform X1 [Vitis vinifera] | 125593.6872 | 0.019 | 0.81 |
| 279 | Unigene17030_All | 149 2101 minus strand PREDICTED: exocyst complex component EXO70A1 [Vitis vinifera] | 74146.20997 | 0.025 | 0.81 |
| 280 | CL4742.Contig1_All | 183 1022 hypothetical protein CISIN_1g044553mg [Citrus sinensis] | 31719.98819 | 0.036 | 0.81 |
| 281 | CL1262.Contig3_All | 455 1855 PREDICTED: serine carboxypeptidase-like 42 [Eucalyptus grandis] &gt;gi\|629103758\|gb\|KCW69227.1\| hypothetical protein EUGRSUZ_F02740 [Eucalyptus grandis] | 52794.34859 | 0.049 | 0.81 |
| 282 | CL1537.Contig1_All | 180 659 PREDICTED: universal stress protein A-like protein [Gossypium raimondii] &gt;gi\|763755946\|gb\|KJB23277.1\| hypothetical protein B456_004G089400 [Gossypium raimondii] | 17396.02957 | 0.087 | 0.81 |
| 283 | Unigene30464_All | 178 1002 minus strand PREDICTED: uncharacterized protein At1g03900 [Vitis vinifera] &gt;gi\|147765951\|emb\|CAN67814.1\| hypothetical protein VITISV_002501 [Vitis vinifera] &gt;gi\|297744166\|emb\|CBI37136.3\| unnamed protein product [Vitis vinifera] | 29974.05698 | 0.145 | 0.81 |
| 284 | CL10580.Contig1_All | 174 1487 minus strand PREDICTED: uncharacterized protein LOC100253140 [Vitis vinifera] | 48228.71105 | 0.167 | 0.81 |
| 285 | CL5406.Contig4_All | 363 1430 PREDICTED: malate dehydrogenase, glyoxysomal [Vitis vinifera] &gt;gi\|147774268\|emb\|CAN65552.1\| hypothetical protein VITISV_033330 [Vitis vinifera] &gt;gi\|297736349\|emb\|CBI25072.3\| unnamed protein product [Vitis vinifera] | 37853.8415 | 0.199 | 0.81 |
| 286 | CL9208.Contig1_All | 130 879 Proteasome subunit alpha type 4 family protein [Populus trichocarpa] &gt;gi\|118484506\|gb\|ABK94128.1\| unknown [Populus trichocarpa] &gt;gi\|222853843\|gb\|EEE91390.1\| Proteasome subunit alpha type 4 family protein [Populus trichocarpa] | 27536.72084 | 0.344 | 0.81 |
| 287 | Unigene9647_All | 246 2948 PREDICTED: probable LRR receptor-like serine/threonine-protein kinase At5g48740 [Vitis vinifera] &gt;gi\|297744356\|emb\|CBI37326.3\| unnamed protein product [Vitis vinifera] | 100667.6718 | 0.013 | 0.82 |
| 288 | Unigene27007_All | 91 2361 minus strand hypothetical protein VITISV_034533 [Vitis vinifera] | 82193.34504 | 0.017 | 0.82 |
| 289 | Unigene12722_All | 41 1402 PREDICTED: protease Do-like 8, chloroplastic isoform X1 [Vitis vinifera] &gt;gi\|731419550\|ref\|XP_010661061.1\| PREDICTED: protease Do-like 8, chloroplastic isoform X1 [Vitis vinifera] &gt;gi\|296082900\|emb\|CBI22201.3\| unnamed protein product [Vitis vinifera] | 48550.46501 | 0.022 | 0.82 |
| 290 | CL2591.Contig7_All | 62 1183 Transporter associated with antigen processing protein 2 isoform 5, partial [Theobroma cacao] &gt;gi\|508774857\|gb\|EOY22113.1\| Transporter associated with antigen processing protein 2 isoform 5, partial [Theobroma cacao] | 39898.57611 | 0.029 | 0.82 |
| 291 | Unigene29808_All | 149 1237 minus strand PREDICTED: uncharacterized protein LOC105180227 [Sesamum indicum] | 40213.63343 | 0.033 | 0.82 |
| 292 | CL7256.Contig1_All | 68 1813 minus strand PREDICTED: glucose-6-phosphate 1-dehydrogenase, chloroplastic isoform X1 [Jatropha curcas] &gt;gi\|643718824\|gb\|KDP29923.1\| hypothetical protein JCGZ_18492 [Jatropha curcas] | 66396.39818 | 0.038 | 0.82 |
| 293 | Unigene10077_All | 116 607 minus strand Adenine nucleotide alpha hydrolases-like superfamily protein [Theobroma cacao] &gt;gi\|508786637\|gb\|EOY33893.1\| Adenine nucleotide alpha hydrolases-like superfamily protein [Theobroma cacao] | 18184.34619 | 0.043 | 0.82 |
| 294 | Unigene20273_All | 348 1523 minus strand PREDICTED: glucose-6-phosphate/phosphate translocator 2, chloroplastic-like [Nelumbo nucifera] &gt;gi\|720064590\|ref\|XP_010276002.1\| PREDICTED: glucose-6-phosphate/phosphate translocator 2, chloroplastic-like [Nelumbo nucifera] &gt;gi\|720064593\|ref\|XP_010276003.1\| PREDICTED: glucose-6-phosphate/phosphate translocator 2, chloroplastic-like [Nelumbo nucifera] | 43135.49495 | 0.059 | 0.82 |
| 295 | CL1949.Contig1_All | 685 3309 PREDICTED: protein transport protein SEC23 [Vitis vinifera] &gt;gi\|731395936\|ref\|XP_010652338.1\| PREDICTED: protein transport protein SEC23 [Vitis vinifera] &gt;gi\|297744089\|emb\|CBI37059.3\| unnamed protein product [Vitis vinifera] | 95928.90573 | 0.059 | 0.82 |
| 296 | CL10985.Contig2_All | 120 1592 minus strand PREDICTED: serine carboxypeptidase-like 40 [Citrus sinensis] | 55776.68736 | 0.063 | 0.82 |
| 297 | CL12200.Contig1_All | 194 1654 minus strand PREDICTED: serine carboxypeptidase-like 45 [Nelumbo nucifera] | 54886.74448 | 0.088 | 0.82 |
| 298 | CL8944.Contig2_All | 122 2281 Non-intrinsic ABC protein 8 isoform 1 [Theobroma cacao] &gt;gi\|508775824\|gb\|EOY23080.1\| Non-intrinsic ABC protein 8 isoform 1 [Theobroma cacao] | 79195.53629 | 0.1 | 0.82 |
| 299 | Unigene33826_All | 268 1005 minus strand Eukaryotic translation initiation factor 6-2 [Glycine soja] | 27019.55554 | 0.146 | 0.82 |
| 300 | Unigene21190_All | 138 533 unknown [Picea sitchensis] | 14216.95656 | 0.174 | 0.82 |
| 301 | Unigene16847_All | 254 1354 PREDICTED: pto-interacting protein 1 isoform X1 [Sesamum indicum] | 39984.32083 | 0.226 | 0.82 |
| 302 | CL9624.Contig1_All | 284 679 minus strand PREDICTED: profilin-1 [Vitis vinifera] | 14389.15366 | 0.235 | 0.82 |
| 303 | Unigene27113_All | 214 873 minus strand Vesicle-associated membrane protein 714 isoform 1 [Theobroma cacao] &gt;gi\|590586666\|ref\|XP_007015768.1\| Vesicle-associated membrane protein 714 isoform 1 [Theobroma cacao] &gt;gi\|590586670\|ref\|XP_007015769.1\| Vesicle-associated membrane protein 714 isoform 1 [Theobroma cacao] &gt;gi\|508786130\|gb\|EOY33386.1\| Vesicle-associated membrane protein 714 isoform 1 [Theobroma cacao] &gt;gi\|508786131\|gb\|EOY33387.1\| Vesicle-associated membrane protein 714 isoform 1 [Theobroma cacao] &gt;gi\|508786132\|gb\|EOY33388.1\| Vesicle-associated membrane protein 714 isoform 1 [Theobroma cacao] | 25082.96015 | 0.255 | 0.82 |
| 304 | Unigene10176_All | 191 1525 minus strand PREDICTED: tubulin beta chain [Elaeis guineensis] | 50773.2797 | 0.445 | 0.82 |
| 305 | CL1757.Contig1_All | 184 3330 PREDICTED: nuclear pore complex protein NUP96 isoform X1 [Vitis vinifera] &gt;gi\|731402999\|ref\|XP_002271967.2\| PREDICTED: nuclear pore complex protein NUP96 isoform X2 [Vitis vinifera] | 120165.6359 | 0.016 | 0.83 |
| 306 | Unigene22992_All | 175 1020 PREDICTED: glycine-rich cell wall structural protein 1.0-like isoform X1 [Populus euphratica] &gt;gi\|743899167\|ref\|XP_011042875.1\| PREDICTED: glycine-rich cell wall structural protein 1.0-like isoform X2 [Populus euphratica] | 29847.9041 | 0.039 | 0.83 |
| 307 | Unigene6418_All | 467 1105 embryo-abundant family protein [Populus trichocarpa] &gt;gi\|222854877\|gb\|EEE92424.1\| embryo-abundant family protein [Populus trichocarpa] | 24982.41215 | 0.061 | 0.83 |
| 308 | Unigene10329_All | 166 1116 polygalacturonase inhibitor [Actinidia deliciosa] | 35680.30221 | 0.069 | 0.83 |
| 309 | CL3799.Contig2_All | 228 1139 PREDICTED: A/G-specific adenine DNA glycosylase isoform X2 [Jatropha curcas] | 33564.6621 | 0.082 | 0.83 |
| 310 | Unigene1153_All | 268 1065 minus strand PREDICTED: syntaxin-71 [Vitis vinifera] &gt;gi\|297741579\|emb\|CBI32711.3\| unnamed protein product [Vitis vinifera] | 29990.49263 | 0.124 | 0.83 |
| 311 | CL10665.Contig1_All | 224 616 PREDICTED: profilin-2 [Gossypium raimondii] &gt;gi\|763787314\|gb\|KJB54310.1\| hypothetical protein B456_009G028400 [Gossypium raimondii] | 14187.00416 | 0.16 | 0.83 |
| 312 | CL4924.Contig1_All | 188 1324 minus strand PREDICTED: TOM1-like protein 2 [Citrus sinensis] &gt;gi\|568851729\|ref\|XP_006479539.1\| PREDICTED: TOM1-like protein 2 [Citrus sinensis] &gt;gi\|568851731\|ref\|XP_006479540.1\| PREDICTED: TOM1-like protein 2 [Citrus sinensis] | 42252.95214 | 0.164 | 0.83 |
| 313 | Unigene29976_All | 178 1200 minus strand Adenosine kinase 2 [Theobroma cacao] &gt;gi\|508726787\|gb\|EOY18684.1\| Adenosine kinase 2 [Theobroma cacao] | 37872.94847 | 0.311 | 0.83 |
| 314 | CL2648.Contig3_All | 116 1447 PREDICTED: tubulin beta chain [Malus domestica] &gt;gi\|694389189\|ref\|XP_009370237.1\| PREDICTED: tubulin beta chain [Pyrus x bretschneideri] &gt;gi\|694389231\|ref\|XP_009370255.1\| PREDICTED: tubulin beta chain [Pyrus x bretschneideri] | 50599.96207 | 0.444 | 0.83 |

**Supplementary Table S3.** Supplementary data of 146 DEPs description.

| No. | Protein_ID | Description | Mass | Protein Coverage | Mean_Ratio |
| --- | --- | --- | --- | --- | --- |
| 1 | Unigene29946_All | 215 2197 minus strand Armadillo repeat only 2 [Theobroma cacao] &gt;gi\|508698949\|gb\|EOX90845.1\| Armadillo repeat only 2 [Theobroma cacao] | 73271.04903 | 0.014 | 0.64 |
| 2 | CL5278.Contig1_All | 99 443 minus strand PREDICTED: macrophage migration inhibitory factor homolog isoform X2 [Jatropha curcas] | 12173.23728 | 0.226 | 1.23 |
| 3 | CL8816.Contig1_All | 126 1064 minus strand PREDICTED: hydroxyphenylpyruvate reductase-like [Eucalyptus grandis] &gt;gi\|629104153\|gb\|KCW69622.1\| hypothetical protein EUGRSUZ_F03036 [Eucalyptus grandis] | 34559.99172 | 0.137 | 1.39 |
| 4 | CL5181.Contig1_All | 57 935 PREDICTED: probable 3-hydroxyacyl-CoA dehydrogenase B0272.3 [Fragaria vesca subsp. vesca] | 31628.44366 | 0.191 | 1.24 |
| 5 | Unigene23730_All | 107 2224 phenylalanine ammonia-lyase [Paeonia lactiflora] | 77461.58519 | 0.319 | 1.23 |
| 6 | CL2698.Contig1_All | 85 2445 minus strand unknown [Picea sitchensis] | 86445.18024 | 0.044 | 0.8 |
| 7 | Unigene26919_All | 82 1695 minus strand hypothetical protein VITISV_039323 [Vitis vinifera] | 59532.69427 | 0.284 | 1.3 |
| 8 | CL10524.Contig1_All | 104 1711 minus strand PREDICTED: 4-coumarate--CoA ligase-like 7 [Prunus mume] | 59032.11349 | 0.039 | 1.26 |
| 9 | Unigene23520_All | 378 1997 PREDICTED: 4-coumarate--CoA ligase-like 7 isoform X1 [Jatropha curcas] &gt;gi\|643737999\|gb\|KDP43987.1\| hypothetical protein JCGZ_05454 [Jatropha curcas] | 59031.02905 | 0.106 | 1.22 |
| 10 | Unigene30383_All | 229 1797 PREDICTED: oxalate--CoA ligase-like [Vitis vinifera] | 56678.32781 | 0.034 | 0.79 |
| 11 | CL5154.Contig1_All | 7 324 minus strand PREDICTED: cinnamoyl-CoA reductase 1 isoform X2 [Vitis vinifera] | 12126.41104 | 0.123 | 1.68 |
| 12 | Unigene2334_All | 73 1044 cinnamoyl-CoA reductase [Cunninghamia lanceolata] | 36119.27441 | 0.068 | 1.39 |
| 13 | Unigene30474_All | 161 1210 minus strand PREDICTED: GDSL esterase/lipase At5g03610 isoform X2 [Vitis vinifera] | 39630.07814 | 0.057 | 1.5 |
| 14 | CL5012.Contig1_All | 179 964 PREDICTED: uncharacterized protein At3g49720 [Vitis vinifera] &gt;gi\|731384077\|ref\|XP_010647997.1\| PREDICTED: uncharacterized protein At3g49720 [Vitis vinifera] &gt;gi\|297736364\|emb\|CBI25087.3\| unnamed protein product [Vitis vinifera] | 28977.18516 | 0.065 | 1.24 |
| 15 | CL861.Contig2_All | 1 1167 Beta-glucosidase 12 [Morus notabilis] &gt;gi\|587838259\|gb\|EXB28968.1\| Beta-glucosidase 12 [Morus notabilis] | 45451.44916 | 0.036 | 0.45 |
| 16 | Unigene22989_All | 50 1021 minus strand hypothetical protein PRUPE_ppa026294mg, partial [Prunus persica] &gt;gi\|462402469\|gb\|EMJ08026.1\| hypothetical protein PRUPE_ppa026294mg, partial [Prunus persica] | 36388.57806 | 0.08 | 0.62 |
| 17 | Unigene10329_All | 166 1116 polygalacturonase inhibitor [Actinidia deliciosa] | 35680.30221 | 0.069 | 0.83 |
| 18 | Unigene29808_All | 149 1237 minus strand PREDICTED: uncharacterized protein LOC105180227 [Sesamum indicum] | 40213.63343 | 0.033 | 0.82 |
| 19 | CL384.Contig6_All | 104 1189 PREDICTED: probable mannitol dehydrogenase [Vitis vinifera] &gt;gi\|297741856\|emb\|CBI33216.3\| unnamed protein product [Vitis vinifera] | 39745.07101 | 0.511 | 1.45 |
| 20 | CL12583.Contig4_All | 206 1288 sinapyl alcohol dehydrogenase-like protein [Populus tremula x Populus tremuloides] | 39574.81366 | 0.399 | 1.31 |
| 21 | CL384.Contig4_All | 424 1500 PREDICTED: probable mannitol dehydrogenase [Vitis vinifera] &gt;gi\|296080872\|emb\|CBI18801.3\| unnamed protein product [Vitis vinifera] | 39361.89483 | 0.54 | 1.27 |
| 22 | CL12515.Contig3_All | 50 775 minus strand PREDICTED: probable mannitol dehydrogenase isoform X2 [Vitis vinifera] &gt;gi\|359497143\|ref\|XP_002266559.2\| PREDICTED: probable mannitol dehydrogenase isoform X1 [Vitis vinifera] | 26448.07123 | 0.211 | 1.21 |
| 23 | CL12515.Contig5_All | 197 1273 PREDICTED: probable mannitol dehydrogenase [Sesamum indicum] | 39671.09245 | 0.081 | 0.61 |
| 24 | CL384.Contig1_All | 143 1228 PREDICTED: probable mannitol dehydrogenase [Vitis vinifera] &gt;gi\|297741856\|emb\|CBI33216.3\| unnamed protein product [Vitis vinifera] | 39685.02002 | 0.569 | 0.7 |
| 25 | CL12114.Contig3_All | 230 1021 hypothetical protein CISIN_1g020966mg [Citrus sinensis] | 28492.32374 | 0.034 | 1.21 |
| 26 | Unigene23562_All | 364 1302 Peroxidase superfamily protein [Theobroma cacao] &gt;gi\|508700137\|gb\|EOX92033.1\| Peroxidase superfamily protein [Theobroma cacao] | 35138.59933 | 0.086 | 0.75 |
| 27 | CL4115.Contig1_All | 46 687 minus strand PREDICTED: 1-Cys peroxiredoxin [Phoenix dactylifera] | 23853.07718 | 0.248 | 0.78 |
| 28 | Unigene30412_All | 494 2197 PREDICTED: calcium-dependent protein kinase 26 isoform X1 [Vitis vinifera] | 64283.84967 | 0.033 | 1.36 |
| 29 | Unigene10175_All | 399 1958 PREDICTED: calcium-dependent protein kinase 29 [Sesamum indicum] | 59039.89507 | 0.087 | 1.23 |
| 30 | CL1735.Contig1_All | 251 715 PREDICTED: calcium-binding allergen Ole e 8-like [Nicotiana tomentosiformis] | 17251.97103 | 0.103 | 1.38 |
| 31 | Unigene16556_All | 41 481 serine/threonine kinase-like [Vitis vinifera] &gt;gi\|28629126\|gb\|AAO49473.1\| putative serine/threonine kinase [Vitis vinifera] &gt;gi\|296082851\|emb\|CBI22152.3\| unnamed protein product [Vitis vinifera] | 16876.51595 | 0.204 | 1.21 |
| 32 | CL6633.Contig1_All | 1037 1585 PREDICTED: calmodulin-like protein 1 [Fragaria vesca subsp. vesca] | 21207.50627 | 0.06 | 0.75 |
| 33 | Unigene6841_All | 3 203 minus strand PREDICTED: calmodulin-like [Eucalyptus grandis] | 7485.796595 | 0.254 | 1.45 |
| 34 | Unigene33798_All | 138 1307 minus strand PREDICTED: probable LRR receptor-like serine/threonine-protein kinase At4g20940 [Vitis vinifera] | 43348.71548 | 0.026 | 1.22 |
| 35 | Unigene26624_All | 157 2481 PREDICTED: leucine-rich repeat extensin-like protein 2 [Gossypium raimondii] | 83600.3259 | 0.019 | 0.78 |
| 36 | Unigene12990_All | 176 2092 minus strand hypothetical protein POPTR_0007s13690g [Populus trichocarpa] &gt;gi\|222853189\|gb\|EEE90736.1\| hypothetical protein POPTR_0007s13690g [Populus trichocarpa] | 69527.55912 | 0.013 | 0.77 |
| 37 | CL12289.Contig7_All | 2 937 minus strand Serine-threonine protein kinase, plant-type, putative [Theobroma cacao] &gt;gi\|508727276\|gb\|EOY19173.1\| Serine-threonine protein kinase, plant-type, putative [Theobroma cacao] | 34929.84719 | 0.038 | 0.77 |
| 38 | CL185.Contig5_All | 190 3042 hypothetical protein POPTR_0011s07140g, partial [Populus trichocarpa] &gt;gi\|550327850\|gb\|EEE98017.2\| hypothetical protein POPTR_0011s07140g, partial [Populus trichocarpa] | 106162.6862 | 0.007 | 0.79 |
| 39 | Unigene9647_All | 246 2948 PREDICTED: probable LRR receptor-like serine/threonine-protein kinase At5g48740 [Vitis vinifera] &gt;gi\|297744356\|emb\|CBI37326.3\| unnamed protein product [Vitis vinifera] | 100667.6718 | 0.013 | 0.82 |
| 40 | CL8209.Contig1_All | 501 1574 hypothetical protein LR48_Vigan02g151400 [Vigna angularis] &gt;gi\|965608744\|dbj\|BAT95235.1\| hypothetical protein VIGAN_08191700 [Vigna angularis var. angularis] | 39242.70343 | 0.162 | 0.75 |
| 41 | Unigene16847_All | 254 1354 PREDICTED: pto-interacting protein 1 isoform X1 [Sesamum indicum] | 39984.32083 | 0.226 | 0.82 |
| 42 | Unigene33691_All | 445 1434 minus strand hypothetical protein PRUPE_ppa007712mg [Prunus persica] &gt;gi\|462405025\|gb\|EMJ10489.1\| hypothetical protein PRUPE_ppa007712mg [Prunus persica] | 36993.17615 | 0.033 | 1.55 |
| 43 | Unigene26517_All | 92 1147 SGT1 [Pelargonium x hortorum] | 39623.20266 | 0.08 | 0.79 |
| 44 | CL983.Contig6_All | 211 2316 minus strand PREDICTED: heat shock protein 83 [Ricinus communis] &gt;gi\|223547557\|gb\|EEF49052.1\| heat shock protein, putative [Ricinus communis] | 80883.04856 | 0.352 | 1.54 |
| 45 | CL3148.Contig1_All | 132 2246 minus strand hypothetical protein AALP_AA8G344900 [Arabis alpina] | 80012.93061 | 0.058 | 1.28 |
| 46 | Unigene30372_All | 158 1246 PREDICTED: probable cysteine proteinase A494 [Jatropha curcas] &gt;gi\|643735102\|gb\|KDP41743.1\| hypothetical protein JCGZ_26761 [Jatropha curcas] | 40413.90516 | 0.135 | 1.43 |
| 47 | Unigene13498_All | 98 1171 minus strand Papain family cysteine protease [Theobroma cacao] &gt;gi\|508723000\|gb\|EOY14897.1\| Papain family cysteine protease [Theobroma cacao] | 39653.46268 | 0.07 | 1.53 |
| 48 | CL2560.Contig1_All | 41 946 PREDICTED: telomere repeat-binding factor 1 [Jatropha curcas] &gt;gi\|643738591\|gb\|KDP44512.1\| hypothetical protein JCGZ_16345 [Jatropha curcas] | 33054.4115 | 0.023 | 0.7 |
| 49 | CL4056.Contig1_All | 253 2232 minus strand Phosphoenolpyruvate carboxylase [Morus notabilis] &gt;gi\|587916195\|gb\|EXC03894.1\| Phosphoenolpyruvate carboxylase [Morus notabilis] | 73700.97709 | 0.086 | 0.82 |
| 50 | CL4702.Contig1_All | 193 1476 PREDICTED: pyruvate dehydrogenase E1 component subunit alpha-3, chloroplastic [Populus euphratica] | 47376.91838 | 0.028 | 1.33 |
| 51 | Unigene1192_All | 144 1178 minus strand PREDICTED: malate dehydrogenase, mitochondrial-like [Nelumbo nucifera] | 36354.15099 | 0.397 | 1.35 |
| 52 | CL5406.Contig1_All | 279 1355 minus strand PREDICTED: malate dehydrogenase, glyoxysomal isoform X1 [Vitis vinifera] | 38297.03706 | 0.237 | 0.73 |
| 53 | CL5406.Contig4_All | 363 1430 PREDICTED: malate dehydrogenase, glyoxysomal [Vitis vinifera] &gt;gi\|147774268\|emb\|CAN65552.1\| hypothetical protein VITISV_033330 [Vitis vinifera] &gt;gi\|297736349\|emb\|CBI25072.3\| unnamed protein product [Vitis vinifera] | 37853.8415 | 0.199 | 0.81 |
| 54 | Unigene47708_All | 1 237 minus strand PREDICTED: fumarate hydratase 2, chloroplastic-like [Camelina sativa] | 8493.448397 | 0.089 | 0.51 |
| 55 | Unigene6945_All | 68 1063 minus strand Succinyl-CoA ligase [ADP-forming] subunit alpha-1, mitochondrial [Gossypium arboreum] | 34860.13786 | 0.298 | 1.26 |
| 56 | Unigene1465_All | 7 1239 minus strand unknown [Picea sitchensis] | 44862.43669 | 0.075 | 3.43 |
| 57 | CL9335.Contig3_All | 120 1520 PREDICTED: dihydrolipoyllysine-residue succinyltransferase component of 2-oxoglutarate dehydrogenase complex 2, mitochondrial-like [Jatropha curcas] &gt;gi\|802588393\|ref\|XP_012070863.1\| PREDICTED: dihydrolipoyllysine-residue succinyltransferase component of 2-oxoglutarate dehydrogenase complex 2, mitochondrial-like [Jatropha curcas] &gt;gi\|643731963\|gb\|KDP39155.1\| hypothetical protein JCGZ_00912 [Jatropha curcas] | 50647.54167 | 0.244 | 1.55 |
| 58 | CL9335.Contig1_All | 128 1528 minus strand PREDICTED: dihydrolipoyllysine-residue succinyltransferase component of 2-oxoglutarate dehydrogenase complex 2, mitochondrial-like [Jatropha curcas] &gt;gi\|802588393\|ref\|XP_012070863.1\| PREDICTED: dihydrolipoyllysine-residue succinyltransferase component of 2-oxoglutarate dehydrogenase complex 2, mitochondrial-like [Jatropha curcas] &gt;gi\|643731963\|gb\|KDP39155.1\| hypothetical protein JCGZ_00912 [Jatropha curcas] | 50660.70022 | 0.221 | 1.34 |
| 59 | CL12225.Contig1_All | 173 1603 PREDICTED: dihydrolipoyllysine-residue succinyltransferase component of 2-oxoglutarate dehydrogenase complex 2, mitochondrial-like [Nelumbo nucifera] | 51919.28339 | 0.266 | 1.22 |
| 60 | CL1219.Contig3_All | 177 1589 minus strand PREDICTED: transcription initiation factor IIF subunit alpha-like [Nelumbo nucifera] &gt;gi\|720045081\|ref\|XP_010270096.1\| PREDICTED: transcription initiation factor IIF subunit alpha-like [Nelumbo nucifera] | 51474.2773 | 0.025 | 1.71 |
| 61 | Unigene416_All | 133 1737 PREDICTED: transcription initiation factor IIF subunit alpha [Vitis vinifera] &gt;gi\|297741818\|emb\|CBI33123.3\| unnamed protein product [Vitis vinifera] | 59361.69389 | 0.028 | 1.31 |
| 62 | CL1509.Contig2_All | 791 2074 minus strand PREDICTED: probable RNA polymerase II transcription factor B subunit 1-1 isoform X1 [Vitis vinifera] &gt;gi\|296090002\|emb\|CBI39821.3\| unnamed protein product [Vitis vinifera] | 48336.60751 | 0.023 | 0.73 |
| 63 | CL3832.Contig1_All | 1000 2622 PREDICTED: scarecrow-like protein 21 [Vitis vinifera] &gt;gi\|731433127\|ref\|XP_010644523.1\| PREDICTED: scarecrow-like protein 21 [Vitis vinifera] | 60699.81211 | 0.018 | 0.67 |
| 64 | Unigene20594_All | 22 624 minus strand PREDICTED: abscisic acid receptor PYL4-like [Populus euphratica] &gt;gi\|743781773\|ref\|XP_011003478.1\| PREDICTED: abscisic acid receptor PYL4-like [Populus euphratica] | 22561.26329 | 0.03 | 2.93 |
| 65 | Unigene26598_All | 415 1434 minus strand PREDICTED: serine/threonine-protein kinase SAPK2 [Vitis vinifera] &gt;gi\|297741826\|emb\|CBI33139.3\| unnamed protein product [Vitis vinifera] | 38679.33115 | 0.047 | 1.29 |
| 66 | Unigene10093_All | 476 1543 PREDICTED: serine/threonine-protein kinase SRK2A isoform X1 [Nelumbo nucifera] | 41251.62244 | 0.121 | 0.81 |
| 67 | Unigene26939_All | 321 2132 minus strand ethylene insensitive 3-like 2 protein [Paeonia lactiflora] | 68983.26292 | 0.018 | 0.56 |
| 68 | CL1552.Contig4_All | 347 1822 PREDICTED: probable serine/threonine-protein kinase At5g41260 [Vitis vinifera] &gt;gi\|297745973\|emb\|CBI16029.3\| unnamed protein product [Vitis vinifera] | 55556.29496 | 0.065 | 0.75 |
| 69 | CL3683.Contig2_All | 318 2063 unnamed protein product [Vitis vinifera] | 65867.5283 | 0.043 | 0.75 |
| 70 | Unigene6818_All | 167 619 PREDICTED: NADH dehydrogenase [ubiquinone] iron-sulfur protein 4, mitochondrial-like isoform X1 [Nelumbo nucifera] | 16930.63409 | 0.106 | 1.58 |
| 71 | Unigene72967_All | 3 263 minus strand NADH dehydrogenase subunit 7 (mitochondrion) [Cannabis sativa] | 10063.16779 | 0.08 | 1.42 |
| 72 | Unigene7144_All | 43 420 Acyl Carrier Protein [Salvia miltiorrhiza] | 14049.12031 | 0.048 | 1.27 |
| 73 | CL10263.Contig2_All | 3 278 PREDICTED: acyl carrier protein 2, mitochondrial [Citrus sinensis] | 10604.29481 | 0.065 | 0.79 |
| 74 | Unigene6860_All | 109 537 PREDICTED: NADH dehydrogenase [ubiquinone] 1 alpha subcomplex subunit 13-B [Brachypodium distachyon] &gt;gi\|944087824\|gb\|KQK23176.1\| hypothetical protein BRADI_1g71700 [Brachypodium distachyon] | 16228.37376 | 0.189 | 0.75 |
| 75 | Unigene1034_All | 92 439 PREDICTED: NADH dehydrogenase [ubiquinone] 1 beta subcomplex subunit 9 [Populus euphratica] | 13488.88038 | 0.138 | 0.8 |
| 76 | Unigene13616_All | 135 974 PREDICTED: succinate dehydrogenase [ubiquinone] iron-sulfur subunit 2, mitochondrial [Ricinus communis] &gt;gi\|223533696\|gb\|EEF35431.1\| succinate dehydrogenase, putative [Ricinus communis] | 32270.11464 | 0.179 | 1.37 |
| 77 | Unigene6929_All | 131 1105 minus strand PREDICTED: cytochrome c1-2, heme protein, mitochondrial-like [Nicotiana tomentosiformis] | 36091.12735 | 0.329 | 0.79 |
| 78 | CL10045.Contig1_All | 138 737 minus strand hypothetical protein CICLE_v10002573mg [Citrus clementina] &gt;gi\|557536055\|gb\|ESR47173.1\| hypothetical protein CICLE_v10002573mg [Citrus clementina] | 22272.50185 | 0.34 | 2.65 |
| 79 | Unigene11080_All | 95 772 unknown [Picea sitchensis] &gt;gi\|116783111\|gb\|ABK22797.1\| unknown [Picea sitchensis] &gt;gi\|116786762\|gb\|ABK24227.1\| unknown [Picea sitchensis] &gt;gi\|224285131\|gb\|ACN40293.1\| unknown [Picea sitchensis] &gt;gi\|224285269\|gb\|ACN40360.1\| unknown [Picea sitchensis] | 25986.7081 | 0.04 | 1.32 |
| 80 | CL2804.Contig2_All | 61 279 PREDICTED: ATP synthase subunit epsilon, mitochondrial [Vitis vinifera] | 8069.968862 | 0.356 | 1.36 |
| 81 | Unigene35030_All | 178 672 PREDICTED: V-type proton ATPase 16 kDa proteolipid subunit [Jatropha curcas] &gt;gi\|802708037\|ref\|XP_012084431.1\| PREDICTED: V-type proton ATPase 16 kDa proteolipid subunit [Jatropha curcas] &gt;gi\|643715691\|gb\|KDP27632.1\| hypothetical protein JCGZ_19637 [Jatropha curcas] &gt;gi\|643723204\|gb\|KDP32809.1\| hypothetical protein JCGZ_12101 [Jatropha curcas] | 16769.80486 | 0.109 | 1.36 |
| 82 | Unigene33449_All | 127 729 ATPase, F1 complex, delta/epsilon subunit [Theobroma cacao] &gt;gi\|508776863\|gb\|EOY24119.1\| ATPase, F1 complex, delta/epsilon subunit [Theobroma cacao] | 21580.37523 | 0.174 | 1.23 |
| 83 | Unigene23323_All | 67 396 PREDICTED: V-type proton ATPase subunit G [Vitis vinifera] &gt;gi\|731399299\|ref\|XP_010653570.1\| PREDICTED: V-type proton ATPase subunit G [Vitis vinifera] &gt;gi\|297741597\|emb\|CBI32729.3\| unnamed protein product [Vitis vinifera] | 12188.33838 | 0.2 | 1.25 |
| 84 | Unigene23909_All | 1 1974 minus strand ATPase 11, plasma membrane-type [Morus notabilis] &gt;gi\|587947943\|gb\|EXC34215.1\| ATPase 11, plasma membrane-type [Morus notabilis] | 73224.58413 | 0.213 | 1.37 |
| 85 | CL6826.Contig4_All | 201 3038 minus strand unknown [Picea sitchensis] | 104435.6091 | 0.151 | 0.8 |
| 86 | CL3857.Contig1_All | 105 2357 PREDICTED: peroxisome biogenesis protein 5 isoform X1 [Vitis vinifera] &gt;gi\|296090052\|emb\|CBI39871.3\| unnamed protein product [Vitis vinifera] | 84442.91577 | 0.017 | 0.79 |
| 87 | CL3248.Contig1_All | 444 1397 minus strand PREDICTED: peroxisome biogenesis protein 7 [Cucumis sativus] &gt;gi\|700194105\|gb\|KGN49309.1\| hypothetical protein Csa_6G519610 [Cucumis sativus] | 36023.43931 | 0.035 | 1.25 |
| 88 | Unigene33138_All | 72 3476 PREDICTED: peroxisome biogenesis protein 1 isoform X1 [Vitis vinifera] | 125593.6872 | 0.019 | 0.81 |
| 89 | Unigene27007_All | 91 2361 minus strand hypothetical protein VITISV_034533 [Vitis vinifera] | 82193.34504 | 0.017 | 0.82 |
| 90 | CL5825.Contig6_All | 346 2415 minus strand PREDICTED: long chain acyl-CoA synthetase 6, peroxisomal [Vitis vinifera] &gt;gi\|297737021\|emb\|CBI26222.3\| unnamed protein product [Vitis vinifera] | 76648.88763 | 0.057 | 0.72 |
| 91 | Unigene15754_All | 73 2058 PREDICTED: long chain acyl-CoA synthetase 1 [Vitis vinifera] &gt;gi\|731395765\|ref\|XP_010652282.1\| PREDICTED: long chain acyl-CoA synthetase 1 [Vitis vinifera] &gt;gi\|297744008\|emb\|CBI36978.3\| unnamed protein product [Vitis vinifera] | 75608.56082 | 0.017 | 0.79 |
| 92 | Unigene27602_All | 63 1538 catalase [Pinus sylvestris] | 57377.33854 | 0.11 | 0.78 |
| 93 | CL10819.Contig2_All | 114 788 PREDICTED: peroxiredoxin-2E-1, chloroplastic [Nelumbo nucifera] | 24261.6139 | 0.084 | 1.3 |
| 94 | CL2744.Contig1_All | 206 1150 minus strand PREDICTED: bifunctional epoxide hydrolase 2 isoform X1 [Vitis vinifera] | 35579.19417 | 0.333 | 1.22 |
| 95 | CL2744.Contig3_All | 89 1039 minus strand PREDICTED: bifunctional epoxide hydrolase 2 [Vitis vinifera] &gt;gi\|297739118\|emb\|CBI28769.3\| unnamed protein product [Vitis vinifera] | 35619.33195 | 0.265 | 1.24 |
| 96 | CL5351.Contig2_All | 196 1200 minus strand cytosolic glyceraldehyde-3-phosphate dehydrogenase [Carthamus tinctorius] | 36680.82202 | 0.755 | 1.31 |
| 97 | CL2097.Contig9_All | 1 246 RecName: Full=Glyceraldehyde-3-phosphate dehydrogenase 2; Short=GAPDH 2 &gt;gi\|15722242\|emb\|CAC37404.1\| glyceraldehyde-3-phosphate dehydrogenase [Mucor racemosus] &gt;gi\|195610190\|gb\|ACG26925.1\| glyceraldehyde-3-phosphate dehydrogenase, cytosolic [Zea mays] | 8355.45541 | 0.098 | 0.67 |
| 98 | CL2097.Contig6_All | 4 246 minus strand glyceraldehyde-3-phosphate dehydrogenase [Medicago truncatula] &gt;gi\|657381425\|gb\|KEH25421.1\| glyceraldehyde-3-phosphate dehydrogenase [Medicago truncatula] | 8454.455766 | 0.136 | 1.24 |
| 100 | Unigene74214_All | 1 258 pyruvate dehydrogenase E1 beta subunit [Picea mariana] | 10065.27869 | 0.093 | 1.21 |
| 101 | CL9015.Contig1_All | 199 1632 PREDICTED: dihydrolipoyllysine-residue acetyltransferase component 5 of pyruvate dehydrogenase complex, chloroplastic [Vitis vinifera] | 50034.11953 | 0.107 | 0.76 |
| 102 | Unigene32955_All | 26 1504 PREDICTED: hexokinase-2, chloroplastic [Ricinus communis] &gt;gi\|223551227\|gb\|EEF52713.1\| hexokinase, putative [Ricinus communis] | 53882.35961 | 0.057 | 0.68 |
| 103 | CL1864.Contig1_All | 250 1713 minus strand PREDICTED: ATP-dependent 6-phosphofructokinase 3-like [Nelumbo nucifera] | 54061.50862 | 0.33 | 1.25 |
| 104 | Unigene283_All | 86 1645 minus strand PREDICTED: ATP-dependent 6-phosphofructokinase 4, chloroplastic isoform X1 [Vitis vinifera] &gt;gi\|297745021\|emb\|CBI38613.3\| unnamed protein product [Vitis vinifera] | 58265.54899 | 0.088 | 1.27 |
| 105 | CL1864.Contig6_All | 204 1625 minus strand hypothetical protein PRUPE_ppa004610mg [Prunus persica] &gt;gi\|645226639\|ref\|XP_008220132.1\| PREDICTED: 6-phosphofructokinase 6 [Prunus mume] &gt;gi\|462420318\|gb\|EMJ24581.1\| hypothetical protein PRUPE_ppa004610mg [Prunus persica] | 52480.51731 | 0.173 | 1.53 |
| 106 | Unigene30381_All | 147 1649 minus strand PREDICTED: pyruvate kinase, cytosolic isozyme [Cucumis sativus] &gt;gi\|700198858\|gb\|KGN54016.1\| hypothetical protein Csa_4G268000 [Cucumis sativus] | 54978.3925 | 0.2 | 1.22 |
| 107 | CL3846.Contig4_All | 133 1206 minus strand hypothetical protein CISIN_1g018326mg [Citrus sinensis] | 38850.85027 | 0.788 | 1.37 |
| 108 | Unigene34006_All | 92 1117 minus strand PREDICTED: aldose 1-epimerase-like [Eucalyptus grandis] &gt;gi\|629119619\|gb\|KCW84109.1\| hypothetical protein EUGRSUZ_B00995 [Eucalyptus grandis] | 37332.88982 | 0.193 | 1.24 |
| 109 | CL2281.Contig2_All | 246 2516 minus strand PREDICTED: subtilisin-like protease [Vitis vinifera] | 81248.52811 | 0.229 | 1.29 |
| 110 | Unigene20395_All | 140 2308 minus strand hypothetical protein PRUPE_ppa002029mg [Prunus persica] &gt;gi\|462406624\|gb\|EMJ12088.1\| hypothetical protein PRUPE_ppa002029mg [Prunus persica] | 79666.1472 | 0.03 | 1.79 |
| 111 | CL4758.Contig2_All | 12 1685 Putative 2-3 biphosphoglycerate independant phosphoglycerate mutase [Vitis vinifera] | 61580.27602 | 0.075 | 1.21 |
| 112 | CL2213.Contig3_All | 242 1378 minus strand PREDICTED: alcohol dehydrogenase [Populus euphratica] | 41889.97953 | 0.24 | 1.34 |
| 113 | Unigene315_All | 3 269 Alcohol dehydrogenase class-P -like protein [Gossypium arboreum] | 10199.20764 | 0.607 | 1.25 |
| 114 | Unigene1907_All | 23 1648 minus strand unknown [Picea sitchensis] | 59436.23916 | 0.046 | 1.91 |
| 115 | CL11560.Contig2_All | 163 1770 minus strand PREDICTED: aldehyde dehydrogenase family 2 member B7, mitochondrial-like [Jatropha curcas] &gt;gi\|643713900\|gb\|KDP26565.1\| hypothetical protein JCGZ_17723 [Jatropha curcas] | 58730.24665 | 0.304 | 1.31 |
| 116 | CL7661.Contig5_All | 359 2251 minus strand hypothetical protein CICLE_v10023278mg [Citrus clementina] &gt;gi\|557543641\|gb\|ESR54619.1\| hypothetical protein CICLE_v10023278mg [Citrus clementina] | 70674.02276 | 0.052 | 1.24 |
| 117 | CL2326.Contig2_All | 339 2477 PREDICTED: beta-galactosidase-like [Jatropha curcas] &gt;gi\|643724628\|gb\|KDP33829.1\| hypothetical protein JCGZ_07400 [Jatropha curcas] | 80229.06382 | 0.043 | 0.76 |
| 118 | CL7778.Contig3_All | 85 1083 Alpha-galactosidase 2 [Theobroma cacao] &gt;gi\|508707082\|gb\|EOX98978.1\| Alpha-galactosidase 2 [Theobroma cacao] | 36814.8657 | 0.027 | 0.77 |
| 119 | Unigene12618_All | 70 2418 PREDICTED: probable galactinol--sucrose galactosyltransferase 2 [Vitis vinifera] | 87159.3054 | 0.018 | 0.82 |
| 120 | Unigene20369_All | 131 1057 PREDICTED: NADP-dependent D-sorbitol-6-phosphate dehydrogenase [Ricinus communis] &gt;gi\|223548017\|gb\|EEF49509.1\| aldo-keto reductase, putative [Ricinus communis] | 35207.03743 | 0.052 | 1.22 |
| 121 | Unigene20303_All | 211 2478 minus strand PREDICTED: 6-phosphofructo-2-kinase/fructose-2,6-bisphosphatase [Vitis vinifera] &gt;gi\|297745867\|emb\|CBI15923.3\| unnamed protein product [Vitis vinifera] | 84156.7809 | 0.016 | 0.76 |
| 122 | Unigene23624_All | 169 1635 minus strand PREDICTED: fructokinase-like 1, chloroplastic [Jatropha curcas] &gt;gi\|643710299\|gb\|KDP24506.1\| hypothetical protein JCGZ_25070 [Jatropha curcas] | 56118.1543 | 0.031 | 0.67 |
| 123 | Unigene10965_All | 2 964 unknown [Picea sitchensis] | 35950.00977 | 0.037 | 1.4 |
| 124 | Unigene13624_All | 201 1142 minus strand PREDICTED: pectinesterase 31 [Vitis vinifera] &gt;gi\|296082952\|emb\|CBI22253.3\| unnamed protein product [Vitis vinifera] | 36150.55233 | 0.041 | 0.57 |
| 125 | Unigene23218_All | 498 2267 PREDICTED: pectinesterase 3 [Vitis vinifera] | 65097.60677 | 0.031 | 0.81 |
| 126 | Unigene17035_All | 177 2468 PREDICTED: putative beta-D-xylosidase [Vitis vinifera] | 83940.56821 | 0.039 | 0.77 |
| 127 | Unigene23639_All | 401 1705 minus strand PREDICTED: UDP-glucuronic acid decarboxylase 1 [Vitis vinifera] | 49226.52503 | 0.051 | 0.74 |
| 128 | CL2108.Contig1_All | 65 1519 flavonol 3-O-glucosyltransferase [Paeonia lactiflora] | 55184.3465 | 0.204 | 1.24 |
| 129 | Unigene23530_All | 127 3246 sucrose-phosphate synthase 1 [Vitis vinifera] &gt;gi\|58825798\|gb\|AAW82754.1\| sucrose-phosphate synthase 1 [Vitis vinifera] | 117752.8939 | 0.064 | 1.29 |
| 130 | CL125.Contig1_All | 72 2459 PREDICTED: sucrose synthase [Vitis vinifera] &gt;gi\|731406211\|ref\|XP_010656083.1\| PREDICTED: sucrose synthase [Vitis vinifera] &gt;gi\|731406213\|ref\|XP_010656084.1\| PREDICTED: sucrose synthase [Vitis vinifera] &gt;gi\|297738510\|emb\|CBI27755.3\| unnamed protein product [Vitis vinifera] | 91596.99882 | 0.094 | 1.5 |
| 131 | Unigene33725_All | 436 2961 minus strand PREDICTED: probable alpha,alpha-trehalose-phosphate synthase [UDP-forming] 9 [Vitis vinifera] | 96564.39208 | 0.025 | 0.82 |
| 132 | CL4425.Contig2_All | 390 1607 alpha-amylase [Hevea brasiliensis] | 46419.77491 | 0.032 | 0.76 |
| 133 | Unigene30486_All | 476 3394 minus strand PREDICTED: 4-alpha-glucanotransferase DPE2 [Prunus mume] &gt;gi\|645278116\|ref\|XP_008244085.1\| PREDICTED: 4-alpha-glucanotransferase DPE2 [Prunus mume] | 113186.5117 | 0.007 | 1.52 |
| 134 | CL4361.Contig2_All | 349 2706 PREDICTED: 1,4-alpha-glucan-branching enzyme 1, chloroplastic/amyloplastic isoform X1 [Vitis vinifera] &gt;gi\|302141663\|emb\|CBI18866.3\| unnamed protein product [Vitis vinifera] | 90073.47032 | 0.051 | 0.79 |
| 135 | Unigene16501_All | 136 1377 hypothetical protein POPTR_0017s04590g [Populus trichocarpa] &gt;gi\|550319385\|gb\|ERP50534.1\| hypothetical protein POPTR_0017s04590g [Populus trichocarpa] | 46300.97414 | 0.027 | 0.82 |
| 136 | Unigene16452_All | 155 1408 minus strand Tyrosine transaminase family protein [Theobroma cacao] &gt;gi\|508707593\|gb\|EOX99489.1\| Tyrosine transaminase family protein [Theobroma cacao] | 46622.02146 | 0.033 | 0.81 |
| 137 | Unigene354_All | 100 1029 Aldolase-type TIM barrel family protein [Theobroma cacao] &gt;gi\|508702691\|gb\|EOX94587.1\| Aldolase-type TIM barrel family protein [Theobroma cacao] | 33111.80381 | 0.068 | 0.78 |
| 138 | CL5962.Contig2_All | 650 1402 minus strand PREDICTED: tryptophan synthase beta chain 1 [Vitis vinifera] &gt;gi\|298204401\|emb\|CBI16881.3\| unnamed protein product [Vitis vinifera] | 27185.49438 | 0.108 | 0.78 |
| 139 | CL11599.Contig1_All | 82 999 PREDICTED: chorismate mutase 3, chloroplastic-like [Nelumbo nucifera] | 35194.95476 | 0.121 | 0.77 |
| 140 | CL1496.Contig1_All | 262 1431 PREDICTED: arogenate dehydratase/prephenate dehydratase 2, chloroplastic-like [Pyrus x bretschneideri] &gt;gi\|694353842\|ref\|XP_009358261.1\| PREDICTED: arogenate dehydratase/prephenate dehydratase 2, chloroplastic-like [Pyrus x bretschneideri] &gt;gi\|694353847\|ref\|XP_009358262.1\| PREDICTED: arogenate dehydratase/prephenate dehydratase 2, chloroplastic-like [Pyrus x bretschneideri] | 43666.47272 | 0.031 | 0.73 |
| 141 | CL5644.Contig3_All | 1571 3421 minus strand PREDICTED: uncharacterized protein LOC101293587 [Fragaria vesca subsp. vesca] | 72266.11116 | 0.01 | 0.71 |
| 142 | CL8944.Contig2_All | 122 2281 Non-intrinsic ABC protein 8 isoform 1 [Theobroma cacao] &gt;gi\|508775824\|gb\|EOY23080.1\| Non-intrinsic ABC protein 8 isoform 1 [Theobroma cacao] | 79195.53629 | 0.1 | 0.82 |
| 143 | CL2591.Contig7_All | 62 1183 Transporter associated with antigen processing protein 2 isoform 5, partial [Theobroma cacao] &gt;gi\|508774857\|gb\|EOY22113.1\| Transporter associated with antigen processing protein 2 isoform 5, partial [Theobroma cacao] | 39898.57611 | 0.029 | 0.82 |
| 144 | Unigene25270_All | 4 525 hypothetical protein VITISV_032357 [Vitis vinifera] | 20278.40633 | 0.029 | 1.35 |
| 145 | Unigene290_All | 109 4473 minus strand PREDICTED: pleiotropic drug resistance protein 1-like [Nelumbo nucifera] | 165114.4987 | 0.025 | 1.4 |
| 146 | Unigene26536_All | 188 4693 minus strand Pleiotropic drug resistance protein 12 [Morus notabilis] &gt;gi\|587885718\|gb\|EXB74575.1\| Pleiotropic drug resistance protein 12 [Morus notabilis] | 170908.789 | 0.029 | 0.82 |

**Supplementary Table S4.** Supplementary data of 43 DEPs description.

| No. | Protein_ID | Description | Mass | Protein Coverage | Mean_Ratio |
| --- | --- | --- | --- | --- | --- |
| 1 | Unigene10175_All | 399 1958 PREDICTED: calcium-dependent protein kinase 29 [Sesamum indicum] | 59039.89507 | 0.087 | 1.23 |
| 2 | Unigene6841_All | 3 203 minus strand PREDICTED: calmodulin-like [Eucalyptus grandis] | 7485.796595 | 0.254 | 1.45 |
| 3 | CL6633.Contig1_All | 1037 1585 PREDICTED: calmodulin-like protein 1 [Fragaria vesca subsp. vesca] | 21207.50627 | 0.06 | 0.75 |
| 4 | CL2560.Contig1_All | 41 946 PREDICTED: telomere repeat-binding factor 1 [Jatropha curcas] &gt;gi\|643738591\|gb\|KDP44512.1\| hypothetical protein JCGZ_16345 [Jatropha curcas] | 33054.4115 | 0.023 | 0.7 |
| 5 | Unigene20594_All | 22 624 minus strand PREDICTED: abscisic acid receptor PYL4-like [Populus euphratica] &gt;gi\|743781773\|ref\|XP_011003478.1\| PREDICTED: abscisic acid receptor PYL4-like [Populus euphratica] | 22561.26329 | 0.03 | 2.93 |
| 6 | CL3832.Contig1_All | 1000 2622 PREDICTED: scarecrow-like protein 21 [Vitis vinifera] &gt;gi\|731433127\|ref\|XP_010644523.1\| PREDICTED: scarecrow-like protein 21 [Vitis vinifera] | 60699.81211 | 0.018 | 0.67 |
| 7 | Unigene10093_All | 476 1543 PREDICTED: serine/threonine-protein kinase SRK2A isoform X1 [Nelumbo nucifera] | 41251.62244 | 0.121 | 0.81 |
| 8 | CL1552.Contig4_All | 347 1822 PREDICTED: probable serine/threonine-protein kinase At5g41260 [Vitis vinifera] &gt;gi\|297745973\|emb\|CBI16029.3\| unnamed protein product [Vitis vinifera] | 55556.29496 | 0.065 | 0.75 |
| 9 | CL5644.Contig3_All | 1571 3421 minus strand PREDICTED: uncharacterized protein LOC101293587 [Fragaria vesca subsp. vesca] | 72266.11116 | 0.01 | 0.71 |
| 10 | CL8944.Contig2_All | 122 2281 Non-intrinsic ABC protein 8 isoform 1 [Theobroma cacao] &gt;gi\|508775824\|gb\|EOY23080.1\| Non-intrinsic ABC protein 8 isoform 1 [Theobroma cacao] | 79195.53629 | 0.1 | 0.82 |
| 11 | CL2591.Contig7_All | 62 1183 Transporter associated with antigen processing protein 2 isoform 5, partial [Theobroma cacao] &gt;gi\|508774857\|gb\|EOY22113.1\| Transporter associated with antigen processing protein 2 isoform 5, partial [Theobroma cacao] | 39898.57611 | 0.029 | 0.82 |
| 12 | Unigene26536_All | 188 4693 minus strand Pleiotropic drug resistance protein 12 [Morus notabilis] &gt;gi\|587885718\|gb\|EXB74575.1\| Pleiotropic drug resistance protein 12 [Morus notabilis] | 170908.789 | 0.029 | 0.82 |
| 13 | CL3857.Contig1_All | 105 2357 PREDICTED: peroxisome biogenesis protein 5 isoform X1 [Vitis vinifera] &gt;gi\|296090052\|emb\|CBI39871.3\| unnamed protein product [Vitis vinifera] | 84442.91577 | 0.017 | 0.79 |
| 14 | Unigene33138_All | 72 3476 PREDICTED: peroxisome biogenesis protein 1 isoform X1 [Vitis vinifera] | 125593.6872 | 0.019 | 0.81 |
| 15 | Unigene27602_All | 63 1538 catalase [Pinus sylvestris] | 57377.33854 | 0.11 | 0.78 |
| 16 | Unigene6818_All | 167 619 PREDICTED: NADH dehydrogenase [ubiquinone] iron-sulfur protein 4, mitochondrial-like isoform X1 [Nelumbo nucifera] | 16930.63409 | 0.106 | 1.58 |
| 17 | Unigene72967_All | 3 263 minus strand NADH dehydrogenase subunit 7 (mitochondrion) [Cannabis sativa] | 10063.16779 | 0.08 | 1.42 |
| 18 | Unigene11080_All | 95 772 unknown [Picea sitchensis] &gt;gi\|116783111\|gb\|ABK22797.1\| unknown [Picea sitchensis] &gt;gi\|116786762\|gb\|ABK24227.1\| unknown [Picea sitchensis] &gt;gi\|224285131\|gb\|ACN40293.1\| unknown [Picea sitchensis] &gt;gi\|224285269\|gb\|ACN40360.1\| unknown [Picea sitchensis] | 25986.7081 | 0.04 | 1.32 |
| 20 | Unigene35030_All | 178 672 PREDICTED: V-type proton ATPase 16 kDa proteolipid subunit [Jatropha curcas] &gt;gi\|802708037\|ref\|XP_012084431.1\| PREDICTED: V-type proton ATPase 16 kDa proteolipid subunit [Jatropha curcas] &gt;gi\|643715691\|gb\|KDP27632.1\| hypothetical protein JCGZ_19637 [Jatropha curcas] &gt;gi\|643723204\|gb\|KDP32809.1\| hypothetical protein JCGZ_12101 [Jatropha curcas] | 16769.80486 | 0.109 | 1.36 |
| 19 | CL2804.Contig2_All | 61 279 PREDICTED: ATP synthase subunit epsilon, mitochondrial [Vitis vinifera] | 8069.968862 | 0.356 | 1.36 |
| 21 | Unigene33449_All | 127 729 ATPase, F1 complex, delta/epsilon subunit [Theobroma cacao] &gt;gi\|508776863\|gb\|EOY24119.1\| ATPase, F1 complex, delta/epsilon subunit [Theobroma cacao] | 21580.37523 | 0.174 | 1.23 |
| 22 | CL10263.Contig2_All | 3 278 PREDICTED: acyl carrier protein 2, mitochondrial [Citrus sinensis] | 10604.29481 | 0.065 | 0.79 |
| 23 | Unigene1034_All | 92 439 PREDICTED: NADH dehydrogenase [ubiquinone] 1 beta subcomplex subunit 9 [Populus euphratica] | 13488.88038 | 0.138 | 0.8 |
| 24 | Unigene6929_All | 131 1105 minus strand PREDICTED: cytochrome c1-2, heme protein, mitochondrial-like [Nicotiana tomentosiformis] | 36091.12735 | 0.329 | 0.79 |
| 25 | CL6826.Contig4_All | 201 3038 minus strand unknown [Picea sitchensis] | 104435.6091 | 0.151 | 0.8 |
| 26 | CL1864.Contig1_All | 250 1713 minus strand PREDICTED: ATP-dependent 6-phosphofructokinase 3-like [Nelumbo nucifera] | 54061.50862 | 0.33 | 1.25 |
| 27 | Unigene30381_All | 147 1649 minus strand PREDICTED: pyruvate kinase, cytosolic isozyme [Cucumis sativus] &gt;gi\|700198858\|gb\|KGN54016.1\| hypothetical protein Csa_4G268000 [Cucumis sativus] | 54978.3925 | 0.2 | 1.22 |
| 28 | CL3846.Contig4_All | 133 1206 minus strand hypothetical protein CISIN_1g018326mg [Citrus sinensis] | 38850.85027 | 0.788 | 1.37 |
| 29 | CL4758.Contig2_All | 12 1685 Putative 2-3 biphosphoglycerate independant phosphoglycerate mutase [Vitis vinifera] | 61580.27602 | 0.075 | 1.21 |
| 30 | CL2213.Contig3_All | 242 1378 minus strand PREDICTED: alcohol dehydrogenase [Populus euphratica] | 41889.97953 | 0.24 | 1.34 |
| 31 | Unigene32955_All | 26 1504 PREDICTED: hexokinase-2, chloroplastic [Ricinus communis] &gt;gi\|223551227\|gb\|EEF52713.1\| hexokinase, putative [Ricinus communis] | 53882.35961 | 0.057 | 0.68 |
| 32 | Unigene23530_All | 127 3246 sucrose-phosphate synthase 1 [Vitis vinifera] &gt;gi\|58825798\|gb\|AAW82754.1\| sucrose-phosphate synthase 1 [Vitis vinifera] | 117752.8939 | 0.064 | 1.29 |
| 33 | Unigene13624_All | 201 1142 minus strand PREDICTED: pectinesterase 31 [Vitis vinifera] &gt;gi\|296082952\|emb\|CBI22253.3\| unnamed protein product [Vitis vinifera] | 36150.55233 | 0.041 | 0.57 |
| 34 | Unigene17035_All | 177 2468 PREDICTED: putative beta-D-xylosidase [Vitis vinifera] | 83940.56821 | 0.039 | 0.77 |
| 35 | CL4425.Contig2_All | 390 1607 alpha-amylase [Hevea brasiliensis] | 46419.77491 | 0.032 | 0.76 |
| 36 | CL5154.Contig1_All | 7 324 minus strand PREDICTED: cinnamoyl-CoA reductase 1 isoform X2 [Vitis vinifera] | 12126.41104 | 0.123 | 1.68 |
| 37 | CL2698.Contig1_All | 85 2445 minus strand unknown [Picea sitchensis] | 86445.18024 | 0.044 | 0.8 |
| 38 | Unigene30383_All | 229 1797 PREDICTED: oxalate--CoA ligase-like [Vitis vinifera] | 56678.32781 | 0.034 | 0.79 |
| 39 | Unigene10329_All | 166 1116 polygalacturonase inhibitor [Actinidia deliciosa] | 35680.30221 | 0.069 | 0.83 |
| 40 | CL384.Contig1_All | 143 1228 PREDICTED: probable mannitol dehydrogenase [Vitis vinifera] &gt;gi\|297741856\|emb\|CBI33216.3\| unnamed protein product [Vitis vinifera] | 39685.02002 | 0.569 | 0.7 |
| 41 | Unigene23562_All | 364 1302 Peroxidase superfamily protein [Theobroma cacao] &gt;gi\|508700137\|gb\|EOX92033.1\| Peroxidase superfamily protein [Theobroma cacao] | 35138.59933 | 0.086 | 0.75 |
| 42 | CL11021.Contig3_All | 118 1188 PREDICTED: UDP-arabinopyranose mutase 1 [Vitis vinifera] | 41156.77351 | 0.162 | 0.75 |
| 43 | Unigene13452_All | 16 3126 minus strand hypothetical protein EUGRSUZ_C00246 [Eucalyptus grandis] | 118620.5938 | 0.014 | 0.76 |

.**Supplementary Table S5.** Supplementary data of gene-specific primers used in qRT-PCR analysis.

| Gene | Forward primer sequence (5*'*-3*'*) | Reverse primer sequence (5*'*-3*'*) |
| --- | --- | --- |
| *CDPK* | CACATCTGCTTCTGGACC | GATTATTGTAGGGGTTGTCG |
| *CAM* | TCACCGAACATACGACGCT | AGAACAGAAGGGGAAGGA |
| *CML* | GTCAGAAACCCCGAGCAGT | GAGAAGAGCGAAGCAAAGC |
| *CIPK* | CTCTGCCTTTGGGTCGTCT | GCGATTCAGCCTTCCTCAC |
| *NAC* | CCAATACCCAGAACCAGC | TATCATTGCCGAGGTGGA |
| *MYB* | CACCCGTCCACCTCTTCTAACT | TTCTGTGCATGACTTGCTACCT |
| *WRKY* | CCGTTGGATTCACAAGTTTAC | CCGATTTGACAATAGAAGCAG |
| *CESA7* | GCAGAACAGGACACCCACA | GATTCCACATTGCTTTACG |
| *PAL* | GTCGTCTTCAGGGCATTC | AGGTTGTGGACCATCAGC |
| *C4H* | TCGGCGGGTATGTCGTAG | CGTGCTGGGTGTTGGAGT |
| *4CL* | GATACGAGCGACGATAGCA | TGGAGAACCAGGAGGATGA |
| *CCR* | TGACAAAGAGCCATCCAAGC | ATCCATTCTTCCAATCCATC |
| *CAD* | TCCATACCCACAATCTCA | GAGGAGCATAGCAGGGAGT |
| *CSE* | GCGCATAGACTTGAAGAAAA | ATTACAGAGGGAAGGGGAGG |
| *COMT* | AAGGACTGCTGCTCCCATAC | TGTTCTTTCTTTTCCACCGT |
| *CCoAOMT* | GTGAGCCTGAGCCAATGAG | CAGTGCTGAGAAGGGAGTA |
| *POD* | ACACTGCGTGAAACTGGT | TCATTGCGAACATACTGC |
| *ABCC2* | ACACTGATGCTTCTGACTTTGCT | GTTGCCACATTATCCGTCTTTAC |
| *ABCG2* | CAAAGCCCAGATTATGCC | CCCCATCCCACTCATTCA |
| *Actin* | GTTGCCCTTGATTACGAG | CAGCTTCCATTCCGATTA |
